# Supplementary material for: Experiences of people taking opioid medication for chronic non-malignant pain: a qualitative evidence synthesis using meta-ethnography
Source: BMJ Open. 2020 Feb 18;10(2):e032988. doi: 10.1136/bmjopen-2019-032988 (PMC7044883; doi:10.1136/bmjopen-2019-032988)
Supplement: Supplementary data [file bmjopen-2019-032988supp001.pdf]

Appendix 1 The eMERGe meta-ethnography reporting guidance

Numbered criteria headings with explanatory reporting criteria	Page
**Phase 1—Selecting meta-ethnography and getting started**	
Introduction<br>1 Rationale and context for the meta-ethnography<br>Describe the gap in research or knowledge to be filled by the meta-ethnography, and the wider context of the meta-ethnography	4
2 Aim(s) of the meta-ethnography<br>Describe the meta-ethnography aim(s)	4
3 Focus of the meta-ethnography<br>Describe the meta-ethnography review question(s) (or objectives)	4
4 Rationale for using meta-ethnography<br>Explain why meta-ethnography was considered the most appropriate qualitative synthesis methodology	4
**Phase 2—Deciding what is relevant**	
Methods<br>5 Search strategy<br>Describe the rationale for the literature search strategy	4/5
6 Search processes<br>Describe how the literature searching was carried out and by whom	4/5
7 Selecting primary studies<br>Describe the process of study screening and selection, and who was involved	5
Findings<br>8 Outcome of study selection<br>Describe the results of study searches and screening	16
**Phase 3—Reading included studies**	
Methods<br>9 Reading and data extraction approach<br>Describe the reading and data extraction method and processes	6
Findings<br>10 Presenting characteristics of included studies<br>Describe characteristics of the included studies	Table1
**Phase 4—Determining how studies are related**	
Methods<br>11 Process for determining how studies are related<br>Describe the methods and processes for determining how the included studies are related: - Which aspects of studies were compared AND- How the studies were compared	6
Findings<br>12 Outcome of relating studies<br>Describe how studies relate to each other	17
**Phase 5—Translating studies into one another**	16
Methods<br>13 Process of translating studies<br>Describe the methods of translation: - Describe steps taken to preserve the context and meaning of the relationships between concepts within and across studies- Describe how the reciprocal and refutational translations were conducted- Describe how potential alternative interpretations or explanations were considered in the translations	16
Findings<br>14 Outcome of translation<br>Describe the interpretive findings of the translation.	17 to 28
**Phase 6—Synthesizing translations**	
Methods<br>15 Synthesis process<br>Describe the methods used to develop overarching concepts (“synthesised translations”)Describe how potential alternative interpretations or explanations were considered in the synthesis	16
Findings<br>16 Outcome of synthesis process<br>Describe the new theory, conceptual framework, model, configuration, or interpretation of data developed from the synthesis	Figure 1
**Phase 7—Expressing the synthesis**	

Discussion<br>17 Summary of findings<br>Summarize the main interpretive findings of the translation and synthesis and compare them to existing literature	28 to 30
18 Strengths, limitations, and reflexivity<br>Reflect on and describe the strengths and limitations of the synthesis:<br>- Methodological aspects—for example,describe how the synthesis Findings were influenced by the nature of the included studies and how the meta-ethnography was conducted.<br>- Reflexivity—for example, the impact of the research team on the synthesis findings	30
19 Recommendations and conclusions<br>Describe the implications of the Synthesis	31

Reference: France et al. BMC Medical Research Methodology (2019) 19:25 https://doi.org/10.1186/s12874-018-0600-0
