# Supplementary material for: Experiences of people taking opioid medication for chronic non-malignant pain: a qualitative evidence synthesis using meta-ethnography
Source: BMJ Open. 2020 Feb 18;10(2):e032988. doi: 10.1136/bmjopen-2019-032988 (PMC7044883; doi:10.1136/bmjopen-2019-032988)
Supplement: Supplementary data [file bmjopen-2019-032988supp002.pdf]

Appendix 2 – example of search terms

Scopus<br>((TITLE-ABS-KEY(buprenorphine or fentanyl or heroin or hydromorphone or methadone or morphine or opium or oxycodone or pentazocine or tramadol or opiate* or opioid*) OR TITLE-ABS-KEY(suboxone or bunavail or zubsolv or dipipanone or diconal or wellconal or diamorphine or duragesic or fentora or actiq or abstral or recivit or effentora or instanyl or pecfent or oxycontin or roxicodone or oramorph or papaveretum or omnopon or fortral)OR TITLE-ABS-KEY(sosegon or "talwin nx" or pethidine or meperidine or demerol or tapentadol or nucynta or palexia or tapal or ultram or zytram))) and ((TITLE-ABS-KEY(qualitative w/5 (theor* or study or studies or research or analysis)) OR TITLE-ABS-KEY(ethno* or emic or etic or phenomenolog* or hermeneutic* or heidegger* or husserl* or colaizzi* or giorgi* or glaser or strauss or (van and kaam*) or (van and manen) or ricoeur or spiegelberg* or merleau) OR TITLE-ABS-KEY(constant w/3 compar*) OR TITLE-ABS-KEY(focus w/3 group*) OR TITLE-ABS-KEY(grounded w/3 (theor* or study or studies or research or analysis)) OR TITLE-ABS-KEY(narrative w/3 analysis) OR TITLE-ABS-KEY(discourse w/3 analysis) OR TITLE-ABS-KEY((lived or life) w/3 experience*) OR TITLE-ABS-KEY((theoretical or purposive) w/3 sampl*) OR TITLE-ABS-KEY("field note*" or "field record*" or fieldnote*) OR TITLE-ABS-KEY(participant* w/3 observ*) OR TITLE-ABS-KEY("action research") OR TITLE-ABS-KEY("digital adj record*" or audiorecord* or taperecord* or videorecord* or videotap*) OR TITLE-ABS-KEY(cooperative and inquir*) OR TITLE-ABS-KEY(co and operative and inquir*) OR TITLE-ABS-KEY(co-operative and inquir*) OR TITLE-ABS-KEY(("semi-structured" or semistructured or unstructured or structured) w/3 interview*) OR TITLE-ABS-KEY((Informal or in-depth or indepth or "in depth") w/3 interview*) OR TITLE-ABS-KEY(("face-to-face" or "face to face") w/3 interview*) OR TITLE-ABS-KEY("ipa" or "interpretive phenomenological analysis") OR TITLE-ABS-KEY(social and construct*) OR TITLE-ABS-KEY("appreciative inquiry") OR TITLE-ABS-KEY(poststructural* or "post structural*" or post-structural*) OR TITLE-ABS-KEY(postmodern* or "post modern*" or post-modern*) OR TITLE-ABS-KEY(feminis*) OR TITLE-ABS-KEY(humanistic or existential or experiential))) and (TITLE-ABS-KEY(pain))
