# Supplementary material for: Experiences of people taking opioid medication for chronic non-malignant pain: a qualitative evidence synthesis using meta-ethnography
Source: BMJ Open. 2020 Feb 18;10(2):e032988. doi: 10.1136/bmjopen-2019-032988 (PMC7044883; doi:10.1136/bmjopen-2019-032988)
Supplement: Supplementary data [file bmjopen-2019-032988supp003.pdf]

Appendix 3 Critical Appraisal Skills Programme (CASP) Quality appraisal tool scores

Study	Q1	Q2	Q3	Q4	Q5	Q6	Q7	Q8	Q9	Q10	Score
**Arnaert 2006**	Yes	Yes	Yes	Yes	?	No	Yes	Yes	Yes	Yes	17/20
**Bergman 2013**	Yes	Yes	Yes	Yes	Yes	No	Yes	?	Yes	Yes	17/20
**Blake 2007**	Yes	Yes	Yes	Yes	Yes	?	Yes	Yes	Yes	Yes	19/20
**Brooks 2015**	Yes	Yes	Yes	Yes	Yes	No	Yes	?	Yes	Yes	17/20
**Buchbinder 2015**	Yes	Yes	Yes	Yes	Yes	No	Yes	Yes	Yes	Yes	18/20
**Chang F 2017**	Yes	Yes	Yes	Yes	Yes	No	?	?	Yes	Yes	16/20
**Chang Y-P 2011**	Yes	Yes	Yes	Yes	Yes	No	Yes	?	?	Yes	16/20
**Coyne 2015**	Yes	Yes	Yes	Yes	Yes	No	Yes	?	Yes	Yes	17/20
**Esquibel 2014**	Yes	Yes	Yes	Yes	Yes	Yes	Yes	Yes	Yes	Yes	20/20
**Frank 2016**	Yes	Yes	Yes	Yes	Yes	Yes	Yes	Yes	Yes	Yes	20/20
**Green 2017**	Yes	Yes	Yes	Yes	Yes	No	Yes	Yes	Yes	Yes	18/20
**Hooten 2011**	Yes	Yes	Yes	Yes	Yes	No	Yes	Yes	Yes	Yes	18/20
**Krebs 2014**	Yes	Yes	Yes	Yes	Yes	?	Yes	Yes	Yes	Yes	19/20
**Matthias 2014**	Yes	Yes	Yes	Yes	Yes	No	Yes	Yes	Yes	Yes	18/20
**McCrorie 2015**	Yes	Yes	?	Yes	Yes	No	Yes	Yes	Yes	Yes	17/20
**Mueller 2017**	Yes	Yes	Yes	Yes	?	No	Yes	Yes	?	Yes	16/20
**Paterson 2016**	Yes	Yes	Yes	Yes	Yes	?	?	Yes	Yes	Yes	18/20
**Penney 2017**	Yes	Yes	?	?	?	?	Yes	?	?	?	13/20
**Rieb 2016**	Yes	Yes	Yes	Yes	Yes	?	Yes	?	Yes	Yes	18/20
**Simmonds 2015**	Yes	Yes	?	Yes	Yes	?	Yes	Yes	?	?	16/20
**St Marie 2016**	Yes	Yes	Yes	Yes	Yes	?	Yes	Yes	Yes	Yes	19/20
**Vallerand 1 2009**	Yes	Yes	Yes	Yes	Yes	No	No	Yes	?	Yes	15/20
**Vallerand 2 2010**	Yes	Yes	Yes	?	Yes	No	No	Yes	?	?	13/20
**Wallace 2014**	Yes	Yes	Yes	Yes	Yes	No	Yes	?	?	Yes	16/20
**Warms 2005**	Yes	Yes	Yes	Yes	Yes	No	No	Yes	Yes	Yes	16/20
**Zgierska 2016**	Yes	Yes	Yes	Yes	Yes	No	Yes	Yes	Yes	Yes	18/20
**Zheng 2013**	Yes	Yes	Yes	Yes	Yes	No	?	Yes	Yes	Yes	17/20
**Al Achkar 2017**	Yes	Yes	Yes	Yes	Yes	No	Yes	Yes	Yes	Yes	18/20
**Matthias 2017**	Yes	Yes	Yes	Yes	Yes	No	Yes	Yes	Yes	Yes	18/20
**Matthias 2018**	Yes	Yes	Yes	Yes	Yes	?	Yes	Yes	Yes	Yes	19/20
**Smith 2018**	Yes	Yes	Yes	Yes	Yes	Yes	Yes	Yes	Yes	Yes	20/20

Legend 1 Critical Appraisal Skills Programme (CASP) questions scoring: Yes =2 ? (Can't Tell) = 1 No = 0

Q1. Was there a clear statement of the aims of the research?
Q2. Is a qualitative methodology appropriate?
Q3. Was the research design appropriate to the aims of the research?
Q4. Was the recruitment strategy appropriate to the aims of the research?
Q5. Was the data collected in a way that addressed the research issue?
Q6. Has the relationship between researcher and participants been adequately considered?
Q7. Have ethical issues been taken into consideration?
Q8. Was the data analysis sufficiently rigorous?
Q9. Is there a clear statement of findings?
Q10. How valuable is the research?
