# Supplementary material for: Experiences of people taking opioid medication for chronic non-malignant pain: a qualitative evidence synthesis using meta-ethnography
Source: BMJ Open. 2020 Feb 18;10(2):e032988. doi: 10.1136/bmjopen-2019-032988 (PMC7044883; doi:10.1136/bmjopen-2019-032988)
Supplement: Author's manuscript [file bmjopen-2019-032988.draft_revisions.pdf]

BMJ Open

BMJ Open is committed to open peer review. As part of this commitment we make the peer review history of every article we publish publicly available.

When an article is published we post the peer reviewers' comments and the authors' responses online. We also post the versions of the paper that were used during peer review. These are the versions that the peer review comments apply to.

The versions of the paper that follow are the versions that were submitted during the peer review process. They are not the versions of record or the final published versions. They should not be cited or distributed as the published version of this manuscript.

BMJ Open is an open access journal and the full, final, typeset and author-corrected version of record of the manuscript is available on our site with no access controls, subscription charges or pay-per-view fees (http://bmjopen.bmj.com).

If you have any questions on BMJ Open's open peer review process please email info.bmjopen@bmj.com

BMJ Open

A systematic review and qualitative evidence synthesis of the experiences of people taking opioid medication for chronic non-malignant pain: a meta-ethnography

Journal:	*BMJ Open*
Manuscript ID	bmjopen-2019-032988
Article Type:	Research
Date Submitted by the Author:	15-Jul-2019
Complete List of Authors:	Nichols, Vivien; University of Warwick, Warwick Clinical Trials Unit, Warwick Medical School<br>Toye, Francine; Oxford University Hospitals NHS Trust, Nuffield Orthopaedic Centre Physiotherapy Research Unit<br>Eldabe, Sam; The James Cook University Hospital<br>Sandhu, Harbinder; University of Warwick, Warwick Clinical Trials Unit, Warwick Medical School<br>Underwood, Martin; University of Warwick, Warwick Clinical Trials Unit, Warwick Medical School; University Hospitals of Coventry and Warwickshire<br>Seers, Kate; University of Warwick, Warwick Research in Nursing, Division of Health Sciences, Warwick Medical School
Keywords:	opioid, patients' views, QUALITATIVE RESEARCH, chronic non-malignant pain, meta-ethnography, qualitative evidence synthesis

SCHOLARONE™
Manuscripts

I, the Submitting Author has the right to grant and does grant on behalf of all authors of the Work (as defined in the below author licence), an exclusive licence and/or a non-exclusive licence for contributions from authors who are: i) UK Crown employees; ii) where BMJ has agreed a CC-BY licence shall apply, and/or iii) in accordance with the terms applicable for US Federal Government officers or employees acting as part of their official duties; on a worldwide, perpetual, irrevocable, royalty-free basis to BMJ Publishing Group Ltd ("BMJ") its licensees and where the relevant Journal is co-owned by BMJ to the co-owners of the Journal, to publish the Work in this journal and any other BMJ products and to exploit all rights, as set out in our licence.

The Submitting Author accepts and understands that any supply made under these terms is made by BMJ to the Submitting Author unless you are acting as an employee on behalf of your employer or a postgraduate student of an affiliated institution which is paying any applicable article publishing charge ("APC") for Open Access articles. Where the Submitting Author wishes to make the Work available on an Open Access basis (and intends to pay the relevant APC), the terms of reuse of such Open Access shall be governed by a Creative Commons licence – details of these licences and which Creative Commons licence will apply to this Work are set out in our licence referred to above.

Other than as permitted in any relevant BMJ Author's Self Archiving Policies, I confirm this Work has not been accepted for publication elsewhere, is not being considered for publication elsewhere and does not duplicate material already published. I confirm all authors consent to publication of this Work and authorise the granting of this licence.

For peer review only

A systematic review and qualitative evidence synthesis of the experiences of people taking opioid medication for chronic non-malignant pain: a meta-ethnography

Mrs Vivien Nichols (1) Dr Francine Toye (2) Professor Sam Eldabe (3) Dr Harbinder Sandhu (1) Professor Martin Underwood (1,4) and Professor Kate Seers (5)

(1) Warwick Clinical Trials Unit
Warwick Medical School
University of Warwick, UK

(2) Physiotherapy Research Unit, Nuffield Orthopaedic Centre,
Oxford University Hospitals NHS Foundation Trust
Oxford, UK

(3) Academic Pain Department
The James Cook University Hospital
Middlesbrough, UK

(4) University Hospitals of Coventry and Warwickshire
Coventry, UK

(5) Warwick Research in Nursing
Warwick Medical School
University of Warwick, UK

Corresponding author:
Mrs Vivien Nichols
Warwick Clinical Trials Unit,
Division of Health Sciences,
Warwick Medical School,
University of Warwick
Coventry CV4 7AL
Tel: 02476574652
Email: V.P.Nichols@warwick.ac.uk

For peer review only
For peer review only - http://bmjopen.bmj.com/site/about/guidelines.xhtml

Abstract

Objective To review qualitative studies of the experience of taking opioid medication for Chronic Non-Malignant Pain (CNMP)

Design A qualitative evidence synthesis using meta-ethnography.

Methods We used a seven step approach from the methods of meta-ethnography. We searched selected databases for qualitative studies which gave patients' views of taking opioid medication for CNMP. Papers were quality appraised using a CASP tool and GRADE-CERQual guidelines were applied. We identified concepts and iteratively abstracted these concepts into a line of argument.

Results We screened 2129 unique citations, checked 153 full texts and 31 met our review criteria. We identified five themes: 1) Reluctant users with little choice; 2) Understanding opioids: the good and the bad; 3) A therapeutic alliance: not always on the same page; 4) Stigma: feeling scared and secretive but needing support; and 5) The challenge of tapering or withdrawal. A new overarching theme of 'constantly balancing' emerged from the data.

Conclusions People taking opioids are constantly balancing tensions, not always wanting to take opioids, but feeling they have no choice because of the pain. They frequently feel judged, were not always 'on the same page' as their health care professional and changes in opioid use were often challenging.

Key words: Opioid, patients' views, qualitative research, chronic non-malignant pain, meta-ethnography, qualitative evidence synthesis.

Word count 4,863

For peer review only - http://bmjopen.bmj.com/site/about/guidelines.xhtml

Strengths and Limitations

- To our knowledge this is the first qualitative evidence synthesis of patients' experiences of taking opioid medications.
- Meta-ethnography provides a thorough, systematic way of synthesising qualitative findings across multiple studies and gives the reviewer's interpretation of the data.
- Using a GRADE-CERQual approach can assist in rating confidence in the review findings.
- Qualitative research that illuminates patients' perspectives can help to shape future approaches to opioid management.

Introduction

Chronic non-malignant pain (CNMP) affects an estimated 11 to 20% of the population in Europe and US respectively and can impact heavily on people's quality of life [1, 2] . Opioid medications are strong painkillers which have a well-established role in the treatment of acute and cancer pain; they have also been advocated for CNMP. Opioids can have distressing side effects as dosages increase such as; constipation, sedation, drowsiness, nausea, decreased concentration and memory, or mood changes [3]. Most people who use opioids develop tolerance to the painkilling effect of opioids, and some become dependent on them. Studies have shown that high opioid usage can also put people's lives at risk [4]. Despite this, the prescription of opioid medication for CNMP has risen sharply in the higher income countries. Few studies of opioids have shown effectiveness beyond 12 weeks follow up. Population

For peer review only - http://bmjopen.bmj.com/site/about/guidelines.xhtml

surveys have shown long-term use to be associated with increased side effects and limited pain relief [3 5 6].

This synthesis of qualitative research was undertaken to underpin a process evaluation for the Improving the Wellbeing of people with Opioid Treated Chronic pain (I-WOTCH) study funded by the National Institute for Health Research. I-WOTCH is a randomised controlled trial evaluating a multi-component education and patient centred group intervention with tapering programme against a control of an advice booklet with a relaxation CD. More information can be found in the main study protocol (in press) and process evaluation protocol (under review) (references to be added).

This qualitative evidence synthesis uses the methods of meta-ethnography to find out what peoples' experiences are of both using opioids for CNMP and their attempts to stop taking them.

Methods

We use Noblit and Hare's 7 stages of meta-ethnographic analysis [7]. We used the new eMERGe reporting guidelines for meta-ethnography to structure our report [8] (*See appendix 1).* The protocol is published in the international prospective register of systematic reviews (PROSPERO) registration number: CRD42017082418. http://www.crd.york.ac.uk/PROSPERO

Step 1 Getting Started.

In order to address what has been labelled an opioid epidemic [9], we need to understand peoples' experiences of being on opioids and of coming off them. Our team was chosen because of its expertise in primary qualitative research and qualitative evidence synthesis specific to chronic pain and opioid prescription.

For peer review only - http://bmjopen.bmj.com/site/about/guidelines.xhtml

Step 2 Deciding what is relevant.

We undertook systematic electronic searches in June 2017 with a rerun in September 2018, appraising relevant papers for quality using the Critical Appraisal Skills Programme (CASP) tool for qualitative research [10]. One researcher (VN) with the assistance of an academic librarian (SJ) searched 7 electronic databases; Medline, Embase, AMED, CINAHL, PsycInfo Web of Science and Scopus (Science citation index and Social Science Citation Index) and forward citation searches. We used search terms, free text and MeSH terms for all opioid drugs as well as their generic names. We combined these with the MeSH term 'pain' and a wide range of MeSH terms and words to describe all types of qualitative research and its analysis based upon a search used by Toye, Seers and Barker in 2017 [11]. The search was limited to those in English regarding humans with no cut-off date. Appendix 2 shows an example of our search terms.

Unique citations were screened independently by 2 researchers (VN ST *see acknowledgements*) against our inclusion and exclusion criteria (see Box 1). Any disagreements were arbitrated by a third researcher (KS). Papers for full text reading were identified and read by two researchers. Quality was assessed using a CASP tool. VN critically appraised the studies and KS independently appraised 10% for consistency. The CASP scores are shown in table 1 and appendix 3. The GRADE-CERQual (Confidence in Evidence from Reviews of Qualitative research) was used to appraise the reviewers' confidence in the research findings [12, 13].

Box 1 Inclusion/Exclusion criteria

Included Studies
Adults (18 years or older) taking or have taken opioid medication in the last five years
Published in English in peer review journals with no time constraint
Must relate to patient perspectives on using opioid medication for chronic non-malignant pain
Must use qualitative methodology (any analytical approach) or mixed quantitative and qualitative methodology with qualitative findings reported separately
Where studies include participants with differing medication we will include studies where the

For peer review only - http://bmjopen.bmj.com/site/about/guidelines.xhtml

experience of those taking opioids is reported separately
**Excluded studies**
Theoretical or methodological papers
Purely quantitative studies or mixed methods studies where the qualitative data are not presented separately
Studies concerning active cancer
Studies concerning headache
Studies concerning any acute, or acute postoperative, pain
Studies concerned only with heath care professional or carer perspectives, or studies of mixed carer/ patient/ professional populations where patient perspectives are not presented separately
Non- English language studies
Theses or conference abstracts which are not peer reviewed

Step 3 Reading the studies

VN read all the studies and KS and FT read half of these papers each (so all were read twice) and all extracted the second order concepts independently. A second order concept is a researcher's interpretation of data in a primary qualitative study [14]. VN, KS and FT met to discuss and reach agreement, and compiled a spreadsheet of all of the concepts extracted from the papers

Step 4 Determining how the studies are related?

VN sorted the concepts into categories by looking for any similarities and differences across all the studies. VN, KS and FT discussed the categorisation of data on multiple occasions. To enable comparison across studies, VN recorded descriptive data about each study (see table 1)

For peer review only

For peer review only

For peer review only - http://bmjopen.bmj.com/site/about/guidelines.xhtml

Table 1 Study characteristics with CASP and GRADE-CERQual relevance ratings

Studies	Country	Data collection / participants	Analytical Approach	Aims *Italics = verbatim quotes*	Comments	CASP score	Relevance
**1 Arnaert et al 2006**[15] Response Phases in Methadone Treatment for Chronic Non-malignant Pain	Canada	Semi-structured interview N=11 (4M/7F)	Content analysis	*"...to develop an understanding and to gain knowledge about the beliefs of patients with CNMP who were taking methadone, and to explore what challenges, if any, existed that affected their beliefs when first starting methadone treatment as an activity in their personal lives."*	Methadone specific	17/20	P
**2 Bergman et al 2013** [16]Contrasting Tensions Between Patients and PCPs in Chronic Pain Management: A Qualitative Study	USA	In-depth interviews N=26 (24m/2F)	Inductive thematic analysis	*"... to develop a better understanding of the respective experiences, perceptions, and challenges both patients with chronic pain and PCPs face communicating with each other about pain management in the primary care setting."*	USA Veteran	17/20	P
**3 Blake et al 2007**[17] Experiences of patients requiring strong opioid drugs for chronic non-cancer pain: a patient initiated study	UK	One focus group N=4 (2M/2F) and interviews N=10 (3M/5F)	Interpretative Phenomenological Analysis	*"...to determine the attitudes and experiences of patients receiving long-term strong opioid medication for chronic non-cancer pain in primary care."*	Key	19/20	R
**4 Brooks et al 2015** [18]Exploring the lived experience of adults using prescription opioids to manage chronic noncancer pain	Canada	In-depth interviews N=9 (4M/5F)	Interpretative Phenomenological Analysis	*"...to explore the lived experience of adults using prescription opioids to manage CNCP, focusing on how opioid medication affected their daily lives."*	Key	17/20	R

For peer review only - http://bmjopen.bmj.com/site/about/guidelines.xhtml

**5 Buchbinder et al 2014** [19]''Is there any way I can get something for my pain?'' Patient strategies for requesting analgesics	USA	Audio recorded clinical encounters N=74 (37M/37F)	Qualitative approach based on conversational analysis	*"We examined the direct and indirect means by which patients express a desire for analgesic medication."*	More for acute back pain in an emergency setting. Not opioid specific although includes information about opioids	18/20	I
**6 Chang Y-P et al 2011** [20] Use of Prescription Opioid Medication among Community-Dwelling Older Adults with Noncancer Chronic Pain	USA	Face to face interview N=21 (13M/8F) ≥65yrs	Content analysis, mixed methods	*"… to: (1) describe older adults' patterns of adherence to their prescription opioid medication regimens and their reasons for these medication use patterns; and (2) examine the associations between adherence of prescription opioids, pain intensity, and pain interference on daily activity."*	Thematically 'thin'	16/20	P
**7 Chang,F et al 2017** [21]Perceptions of Community-Dwelling Patients and Their Physicians on OxyContin ® Discontinuation and the Impact on Chronic Pain Management	Canada	Pt interviews N=13 1M/ 12F	Not specified, no references given	*"… to explore the perceptions of patients with chronic pain and their physicians on OxyContin discontinuation and the impact that it had on chronic pain care and management."*	Key	16/20	P
**8 Coyne et al 2015** [22] Assessment of a Stool Symptom Screener and Understanding the Opioid-Induced Constipation Symptom Experience	USA	Cognitive interview semi structured think-aloud approach N=66 (27M/39F)	Content analysis	*"…to evaluate the content validity of the Stool Symptom Screener by assessing patient understanding of its items, patient ability to differentiate among response options, and patient perceptions about the use of a 2-week recall period for evaluating stool symptoms, BMs, and laxative use.* *- to evaluate how patients describe their*	PROM specific with additional questions about constipation	17/20	I

For peer review only - http://bmjopen.bmj.com/site/about/guidelines.xhtml

				constipation experience and to understand whether this differs between patients who frequently use laxatives and those who do not."			
**9 Esquibel et al 2014** [23] Doctors and patients in pain: Conflict and collaboration in opioid prescription in primary care	USA	In depth interviews with pts with CNCP receiving opioids and their physicians. N=21 pts (8M/13F)	Immersion/ crystallization process to generate a thematic codebook. Mixed methods with some quant. data analysis	*"...to better understand the effects of COT [chronic opioid therapy]on the doctor–patient relationship."*	Key	20/20	R
**10 Frank et al 2016** [24] Patients' Perspectives on Tapering of Chronic Opioid Therapy: A Qualitative Study	USA	Semi-structured interviews N=24 (11M/13F)	Team based, mixed inductive and deductive approach guided by the Health Belief Model	*"... to explore patients' perspectives on opioid tapering."*	Key	20/20	R
**11 Green et al 2017** [25] Perpetuating stigma or reducing risk? Perspectives from naloxone consumers and pharmacists on pharmacy-based naloxone in 2 states	USA	8 focus groups (only 2 with patients with chronic pain) N=15 (8M/7F)	Thematic analysis. Content analysis with a collaborative codebook development process	*"...to explore the attitudes of various stakeholders toward pharmacy-based naloxone and opioid medication safety and to capture the range of initial experiences with pharmacy naloxone in 2 states with pharmacy naloxone policies."*	Naloxone specific thematically thin	18/20	P

For peer review only - http://bmjopen.bmj.com/site/about/guidelines.xhtml

**12 Hooten et al 2011** [26] Smoking Cessation and Chronic Pain: Patient and Pain Medicine Physician Attitudes	USA	N = 18 Interviews N= 15 and focus group N=3 10M/8F	Participatory research, mixed methods - Thematic and content analysis	*"...to determine the attitudes and beliefs of both patients and pain medicine physicians regarding smoking cessation interventions applied during pain therapy."*	Main focus smoking cessation	18/20	I
**13 Krebs et al 2014** [27] Barriers to Guideline-Concordant Opioid Management in Primary Care—A Qualitative Study	USA	Semi structered interviews N= 26 patients (14 PC physicians) 13M/13F	Immersion crystallization approach	*"...to better understand primary care physicians' and patients' perspectives on recommended opioid management practices and to identify potential barriers and facilitators of guideline concordant opioid management in primary care."*	USA specific barriers to guideline use	16/20	P
**14 Matthias et al 2013** [28] Communicating about opioids for chronic pain: A qualitativestudy of patient attributions and the influence of thepatient–physician relationship	USA	Audio Recordings of primary care visits and Semi-structured interviews after clinic visit. N=30 26M/4F	Immersion/crystallization approach	*"...to advance understanding about communication about opioids by directly capturing clinical communication about opioids, as well as interviewing patients to gain insight into communication patterns and their broader relationships with their physicians, and how these relationships shape communication about opioids."*	USA veterans medical centre.	18/20	R
**15 McCrorie et al 2015**[29] Understanding long-term opioid prescribing for non-cancer pain in primary care: a qualitative study	UK	Semi structured interviews with patients (focus groups with GPs) N=23	Grounded approach for thematic analysis. Constant comparison	*"... to understand the processes which bring about and perpetuate long-term prescribing of opioids for chronic, non-cancer pain. We interviewed patients and GPs about their experiences, beliefs and expectations of analgesia prescribing, to improve understanding of how problematic long-term*	Key	17/20	R

For peer review only

		6M/17F		*opioid prescribing becomes established".*			
**16 Mueller et al 2016** [30] Attitudes Toward Naloxone Prescribing in Clinical Settings: A Qualitative Study of Patients Prescribed High Dose Opioids for Chronic Non-Cancer Pain	USA	Semi-stuctured interview N=24 8M/16F	Ethnographic iterative methods. Inductive and deductive approaches	*"This study assessed the knowledge and attitudes toward naloxone prescribing among non-cancer patients prescribed opioids in primary care."*	Naloxone specific	16/20	I
**17 Paterson et al 2016** [31] Resisting Prescribed Opioids: A Qualitative Study of Decision Making in Patients Taking Opioids for Chronic Noncancer Pain	Australi a	Qualitative individual interviews N=20	Constant comparison and decision making explored in depth and with a thematic analysis utilizing a published "Model of medicine-taking"	"…*to identify the varying influences on patients' decisions about their use of prescribed long-term opioids. The research questions are: 1) does this conceptual Model of medicine-taking apply to people taking prescribed opioids, 2) is the concept of "resistance" to medication useful in this context, and 3) does this concept lead to new insights which may improve communication and shared decision making between"*	Key. Patients interested in non-medication pain management options.	18/20	P
**18 Penney et al 2016** [32] Provider and patient perspectives on opioids	USA	11 Focus groups N=80 and	Thematic coding ref Ryan, Bernard	*"…to identify the practical issues patients and providers face when accessing alternatives to opioids, and how multiple parties view these*	FG and interview questions not opioid specific. No	13/20	P

For peer review only - http://bmjopen.bmj.com/site/about/guidelines.xhtml

and alternative treatments for managing chronic pain: a qualitative study		individual interviews N=10 27M/63F	2003	*issues."*	demographics		
**19 Rieb et al 2016** [33] Withdrawal-associated injury site pain (WISP): a descriptive case series of an opioid cessation phenomenon	Canada	In person semi structured interviews N=21 14M/7F	Deductive and inductive approaches. Use of survey and emergent interview themes	*"…. to document the existence and characteristics of this pain phenomenon that we have named withdrawal-associated injury site pain (WISP)."*	Thematically thin. Specific to 2 weeks post withdrawal of opioids	18/20	P
**20 Simmonds et al 2015** [34] A Qualitative Study of Veterans on Long-Term Opioid Analgesics: Barriers and Facilitators to Multimodality Pain Management	USA	Semi structured focus groups x 3 N=25 17M/8F	Grounded theory-informed approach. Framework provided by the theory of planned behaviour	*"…to examine barriers and facilitators to multimodality chronic pain care among veterans on high-dose opioid analgesics for chronic non-cancer pain."*	USA veteran specific.	16/20	P
**21 St Marie et al 2016** [35] Primary care experiences of people who live with chronic pain and receive opioids to manage pain: A qualitative methodology	USA	Semistructured interviews. N=12 6M/6F	Thematic and interpretive analyses	*"…to provide a deeper understanding of the experiences, issues, and challenges that people face who live with chronic pain and received opioids to help manage their pain in primary care. The following research questions were addressed: (a) What are the experiences of individuals who live with chronic pain and receive opioid pain medications to manage*	9/12 had a history of substance use disorder	19/20	R

For peer review only - http://bmjopen.bmj.com/site/about/guidelines.xhtml

				their pain in primary care? (b) What have been their healthcare experiences as they strive to manage their pain?"			
**22 Vallerand et al 1 2009** [36] Chronic Opioid Therapy for Nonmalignant Pain: The Patient's Perspective. Part I—Life Before and After Opioid Therapy	USA	In depth serial interviews N=22 6M/16F	Phenomenologic study / Constant comparative method	*"...to examine the lived experience of adults receiving opioid therapy for relief of chronic non-malignant pain through the examination of data obtained through serial taper recorded interviews."*	Same cohort as study below.	14/20	R
**23 Vallerand et al 2 2010** [37] Chronic Opioid Therapy for Non-malignant Pain: The Patient's Perspective. Part II— Barriers to Chronic Opioid Therapy	USA	In depth serial narrative interviews N=22 6M/16F	Phenomenologic study / Constant comparative method	*"...to elucidate the essence of the lived experience of patients receiving chronic opioid therapy for chronic non-malignant pain through examining their life narratives."*	Same cohort as study above. Medication costs and medication may be USA specific.	13/20	R
**24 Wallace et al 2014** [38] Voices that may not otherwise be heard: a qualitative exploration into the perspectives of primary care patients living with chronic pain	USA	Photovoice photos, Interviews 1st N=31 2nd N=25 , and focus groups N=19	Grounded theory	*"...this study examined the utility of a combination of qualitative methods (Photovoice, one-on-one interviews, and focus groups) in examining the daily experiences of primary care patients living with chronic pain".*	All receiving opioid meds for CNCP for at least 6ms	16/20	R
**25 Warms et al 2005** [39] There are a few things you did not ask about my pain: writing on the margins of a survey	USA	Comments written in the margins of a questionnaire N=797	Content analysis	*"...to determine the characteristics of those who wrote comments* [written in the margin of survey questionnaire] *and to understand what was being communicated in their comments."*	Primarily about spinal cord injury and amputation.	16/20	P

For peer review only - http://bmjopen.bmj.com/site/about/guidelines.xhtml

**26 Zgierska et al 2016** [40] Mindfulness Meditation-Based Intervention Is Feasible, Acceptable, and Safe for Chronic Low Back Pain Requiring Long-Term Daily Opioid Therapy	USA	Qual data on treatment satisfaction and experience N= 17	Qualitative analysis methods. Grounded theory	*"... to determine feasibility, acceptability, and safety of an MM-based intervention in patients with CLBP requiring daily opioid therapy."*	Focus is on evaluating an intervention	18/20	I
**27 Zheng et al 2013** [41]Chaos to Hope: A Narrative of Healing	Australi a	In depth qual interviews (N=20) 10M/10F	Illness narratives. Thematic analysis	*"...to investigate the progression of the illness and opioid journeys of people who are taking opioids for chronic non-cancer pain".*	Key Qualitative study nested in a RCT investigating the role of acupuncture in reducing opioid medication consumption by patients with chronic non-cancer pain	17/20	P
**Rerun of search**							
**28 Al Achkar et al 2017** [42] Exploring perceptions and experiences of patients who have chronic pain as state prescription opioid policies change: a qualitative study in Indiana	USA	N=9 3M/6F	Inductive emergent thematic analysis	*"...to evaluate the impact of Indiana's opioid prescription legislation on the patient experiences around pain management."*	USA state specific	19/20	R
**29 Matthias et al 2017** [43] "I'm Not Gonna Pull	USA	N=37 12M/25F	Inductive approach,	*"...to understand communication processes related to opioid tapering."*	Key	19/20	R

For peer review only - http://bmjopen.bmj.com/site/about/guidelines.xhtml

the Rug out From Under You": Patient-Provider Communication About Opioid Tapering			constant comparison				
**30 Matthias et al 2018** [44] "I Was a Little Surprised": Qualitative Insights From Patients Enrolled in a 12-Month Trial Comparing Opioids With Nonopioid Medications for Chronic Musculoskeletal Pain	USA	N=34 28M/6F	Inductive approach, constant comparison	*"…to understand patients' experiences with the SPACE trial including their views and anticipated benefits of opioids, versus non - opioids, experiences with the intervention and to what extent expectations were met after completing the study."*	Only used data of 18 who had experience of opioid medication	18/20	R
**31 Smith et al 2018** [45] Seeking Chronic Pain Relief: A Hermeneutic Exploration	USA	N=15 4M/11F	Concurrent embedded mixed methods design, Heideggerian hermeneutic phenomenolog ical approach	*"This research presents an interpretation of the experience of seeking pain relief for a group of people taking opioid pain medications whose pain is not adequately controlled"*	Key but recruited through internet Specifically uncontrolled pain	20/20	P

Legend: GRADE-CERQual Relevance component: R= Relevant, P= Partial relevance, I= Indirect relevance, U=Uncertain relevance

For peer review only - http://bmjopen.bmj.com/site/about/guidelines.xhtml

Step 5 Translating studies into each other

Patterns and associations between categories were explored and all researchers felt that a line of argument approach as defined by Noblit and Hare [7] would be the most useful method to interpret the data.

Step 6 Synthesising Translations

Agreement was reached by clearly defining the over-arching or 3rd order concepts arising from the data. A third order concept is the reviewers' interpretation of second order concepts.

Step 7 Expressing the synthesis

We developed a conceptual model to show how the themes related to each other in a line of argument. (see figure 1)

Insert figure 1 about here.

Patient and Public Involvement

We did not involve patients or the public in our work.

Findings

Two reviewers VN and ST screened 2994 titles or abstracts (after the removal of duplicates from the 5064 citations retrieved) and identified 153 full texts of interest. Two reviewers VN and KS read these and 122 were excluded. Reasons are given in the PRISMA flowchart, see figure 2. The reviewers agreed to include 31 studies. The included studies were from US (24), Canada (4) UK (2), and Australia (2) and used a range of qualitative methods.

We report the 4 facets of GRADE-CERQual for all papers. 1) Methodological 2) Confidence 3) Relevance 4) Adequacy of data - See table 2 below.

For peer review only - http://bmjopen.bmj.com/site/about/guidelines.xhtml

Table 2 Confidence in review findings: GRADE-CERQual assessment

Review findings	Studies contributing	Methodological limitations	Relevance	Coherence	Adequacy of data
Reluctant users with little choice	1,3,4,5,6,7,17,18,21,22,26,27,30 (13 studies)	7 no concerns	4 Relevant<br>6 Partial<br>2 Indirect	No concerns	No concerns
Understanding opioids: the good the bad	1,3,7,9,10,11,15,16,17, 23,25, 27,29. (13 studies)	11 no concerns<br>1 minor concerns	6 Relevant<br>5 Partial<br>1 Indirect	No concerns	No concerns
A therapeutic alliance: not always on the same page	1,2,3,4,5,7,9,10,11,13,14,15,16,17,18,19,20,21,22,23,24,25,26,28,29,31(26 studies)	23 no concerns<br>3 minor concerns	10 Relevant<br>10 Partial<br>3 Indirect	No concerns	No concerns
Stigma: feeling scared, and secretive but needing support	1,2,3,4,7,9,10,14,16,17,18,20,21,22,23,24,27,28,31 (19 studies)	11 no concerns<br>3 minor concerns	8 Relevant<br>8 Partial<br>1 Indirect	No concerns	No concerns
The challenge of tapering/ withdrawal from opioids	7,10,18,19,30,31	5 no concerns<br>1 minor concerns	3 Relevant<br>3 Partial	Minor concerns	Minor concerns

Legend of GRADE-CERQual component scoring:

Methodological limitations, Coherence, Adequacy of data	No or very minor concerns<br>Minor concerns<br>Moderate concerns<br>Serious concerns
Relevance:	Relevant<br>Partial<br>Indirect<br>Uncertain

For peer review only

Synthesis of Findings

We abstracted five themes from the 2nd order concepts. Table 3 below shows how each study contributed to each theme. We have illustrated each concept with exemplary quotations.

Table 3 Themes apparent in each study

Author date	RU	U	TA	S	TW
1 Arnaert and Ciccotosto 2006	X	X	X	X	
2 Bergman, Matthias et al 2013			X	X	
3 Blake, Ruel et al 2007	X	X	X	X	

For peer review only - http://bmjopen.bmj.com/site/about/guidelines.xhtml

4 Brooks, Unruh et al 2015	X		X	X	
5 Buchbinder, Wilbur et al 2014	X		X		
6 Chang Y-P, Wray et al 2011	X				
7 Chang,F and Ibrahim,S 2017	X	X	X	X	X
8 Coyne, Currie et al 2015					
9 Esquibel and Borkan 2014		X	X	X	
10 Frank, Levy et al 2016		X	X	X	X
11 Green, Case et al 2017		X	X		
12 Hooten, Vickers et al 2011					
13 Krebs, Bergman et al 2014			X		
14 Matthias,Krebs et al 2013			X	X	
15 McCrorie, Closs et al 2015		X	X		
16 Mueller, Koester et al 2016		X	X	X	
17 Paterson, Ledgerwood 2016	X	X	X	X	
18 Penney, Ritenbaugh et al 2016	X		X	X	X
19 Rieb, Norman et al 2016			X		X
20 Simmonds, Finley et al 2015			X	X	
21 St Marie et al 2015	X		X	X	
22 Vallerand et al 1 2009	X		X	X	
23 Vallerand et al 2 2010		X	X	X	
24 Wallace et al 2014			X	X	
25 Warms et al 2005 o		X	X		
26 Zgierska et al 2016	X		X		
27 Zheng et al 2013	X	X		X	
**Studies from search rerun**	**RU**	**U**	**TA**	**S**	**TW**
28 Al Achkar et al 2017			X	X	X
29 Matthias et al 2017		X	X		X
30 Matthias et al 2018	X				
31 Smith et al 2018			X	X	

Legend:
RU = Reluctant users with little choice
U = Understanding about opioids: the good and the bad
TA = A therapeutic alliance: not always on the same page
S = Stigma: feeling scared, and secretive but needing support
TW = The challenges of tapering or withdrawal
X = theme present in paper

For peer review only

1) Reluctant users with little choice

This describes a resistance or hesitancy to take opioids mainly due to concerns about side effects or addiction, although they felt there were no other options available.

> *"I don't want to become addicted, if I'm going to become addicted then as far as I'm concerned I'm a druggie, so I might as well not be here anyway, so I don't want to become addicted…"* Blake et al Pg103

"I just didn't want to go on them because I mean once you get on them that's it, you're sort of stuck on them. I didn't want to take morphine at first because there was a girl that I went through one of the courses with and she always seemed really dopey and drugged up so it took them a long while to talk me to into taking the morphine because I didn't want to be like that. Zheng et al pg 1832

Some spoke of underusing or were keen to reduce their medications when possible. There was a dislike of being on long term medication and some thought that it would not relieve their pain.

"I don't want to do that [take more morphine]. *I want to stay on as little as I possibly can because there might come a time when I need more and I don't want to be on high doses. I've always tried to keep it at a minimum amount of tablets each day…"* Blake et al Pg 105

Even though some were reluctant there were other instances of dramatic improvement in people's lives. This then weighted their choice to stay on the opioids.

*"I mean it is just like a miracle as far as I am concerned. It is like knowing it [*the pain*] is there but you have the instruments to prevent it from getting out and [be]coming a roaring demon."* Vallerand et al 1pg 170

"But opiates, that's my way of life. There would be no life if I didn't have this. And I thank God for them because without them I'd be…well I wouldn't be. I just couldn't go on. I would have committed suicide a long time ago. And I say that truthfully cause you could not live like that, with that constant, constant pain. But, with the opiates it's made it possible to be able to have a part of a life, you know." Brooks et al pg 20

For peer review only - http://bmjopen.bmj.com/site/about/guidelines.xhtml

2) Understanding opioids: the good and the bad

This describes patients' knowledge or understanding about opioids which had generally been acquired ad hoc and slowly over time, from pharmacists, patient package inserts in their medication, leaflets, the internet, television programmes and from doctors, especially doctors at the pain clinic.

> *"When you see it in the media, when you see it on the television, you think if you're taking regular morphine you must be in a pretty bad way, you know."* Blake et al Pg103

> *"I always ask before I go on a medication, what are the side effects, I was told I may experience constipation; nothing else was explained to me."* Paterson et al pg 721

There was often poor knowledge about using opioids for chronic pain, and about addiction, overdose risk and side effects.

> *"There's not too much education about it [overdose]… When I first started taking it [the opioid medication], no one told me about OD[overdose]or anything about that. Because I was taking it not [as] prescribed…I was just like when I felt pain I would just take like five or six of them or whatever. Then at the end, I'd run out."* Mueller et al pg 279

Patients often had to defend their usage and this added to their stress especially when they felt their healthcare professionals lacked an understanding of the place for opioids in the treatment of CNMP or were cautious about using them.

> *"The concern is that if they increase my opioid dosage, I could stop breathing. It's ridiculous."* Frank et al Pg1841

> *''There are still a lot of doctors out there that are against it. They think it is bad. Bad medicine. Bad practice.''* Vallerand et al 2 pg 129

For peer review only - http://bmjopen.bmj.com/site/about/guidelines.xhtml

In contrast, some people felt well informed which either produced more concern or gave the patients confidence in their opioid regime.

> *"... and from what I've read up, because I like to, sort of, keep on top of things, that it's an opium based drug, so you will build up some tolerance and you will build up [becomes tearful] And you will potentially become sort of addicted to it, if you like."* McCrorie et al pg 3
>
> *"Under Dr A [pain clinic] I've learnt more. And my concern has been, well it was initially the possibility of addiction, but she has assured me that I'm not showing no signs of addiction at all. I may have some withdrawal problems"* Paterson et al Pg 721

3) A therapeutic alliance: not always on the same page

This describes a therapeutic alliance or the relationship between patients and their health care providers which was considered important.

Overall there was a feeling that HCPs and patients were often 'not on the same page' about opioid usage.

> *"My family doctor…does not want me to be dependent on heavy pain meds, so I am intensely miserable 99% of the time."* Warms et al pg 252

Some patients felt they were not listened to and were frustrated by a lack of empathy from physicians regarding their pain experience.

> *"I frequently have difficulty with the residents (doctors in training) explaining why these drugs, this many drugs…Finally Dr. [family physician] wrote a note in my file – stop harassing [participant's name]. This is what she gets and why she gets it. And they did stop but it was inconvenient. For instance, they would not prescribe me three months at a time. I would be dispensed one month at a*

For peer review only - http://bmjopen.bmj.com/site/about/guidelines.xhtml

time. And for someone who had been taking the same drugs for 10 years I found that condescending. " Brooks et al pg 18

A reluctance to prescribe from GPs and pharmacists and the use of opioid contracts or a restriction of medication were often considered punitive.

> *"It kind of made me feel like I was doing something wrong, which I wasn't, but I signed a contract. You know, what would I be without my meds?"* Krebs et al pg 1152

> *"And I told my doctor that, that I wanted so I could sleep through the night. And now he, well, I'll give you 10, but it's got to last. Like he treats me like a drug addict."* Penney et al pg 6

The healthcare system often worked against a therapeutic alliance with lack of continuity or care or frequent visits which fed into mistrust. Patients complained that provider turnover affected their ability to receive individualised care; conversations about pain and treatment options often had to be started over again from scratch.

> "*I don't have the same doctor long enough to know*". Bergman et al pg 1693

However having blood or urine tests for levels of opioids and regular checks were seen by some as being cared for.

> "*I would say, 'I have this agreement and you don't have to sign it if you don't want to, but I would like to go over it with you. These are suggestions because this medication is addictive, it is dangerous, and I just want to make sure you're aware.' I think if you really want to make it where people are not hostile, say they have to have a urine test every 6 months, everybody, and that 'it's a policy because we care about all of you.'"* Krebs et al pg 1152

Some talked of the need for good relationships built on trust, shared decision making and knowledgeable specialists who communicate well.

For peer review only - http://bmjopen.bmj.com/site/about/guidelines.xhtml

"I wouldn't say I researched it to that depth, you know, I read a little bit about, and asked a lot of questions at my doctor, and then we decided." Paterson pg 722

4) Stigma: feeling scared and secretive but needing support

This describes feelings of stigma and fear which people expressed directly in relation to their opioid usage. This includes peoples' negative attitudes from family, medical professionals and work colleagues which lead to them feeling stigmatised and judged for taking opioids.

"So I'm constantly trying to clean up because I think people are going to judge me. 'Oh, because she's on all this medication, ooh, she can't look after her children.'" Paterson et al pg 724

"As soon as you mention to someone that you are on pain medication it's, 'Oh my god, you've got to get off it.' It is viewed as weak. Somehow I am weak for being on this medication." Vallerand et al 2 pg 128

To protect themselves some chose to keep their opioids a secret.

"But you know, after 2 years of pain, you are physically exhausted, mentally exhausted and depressed. So, I take my medication and I hide it at the bottom of my drawer. It's my secret life. It's always a secret, and I've got to hide it and not tell anyone." Vallerand et al 1 pg 169

Some people made a conscious decision about who they could tell and who they couldn't due to negative reactions. Relationships suffered when patients felt unsupported.

"My son told me I was a drug addict. He did. He really did. He was to the point, he didn't know what he could do for me. It really was that bad." Vallerand et al 2 pg 128

For peer review only - http://bmjopen.bmj.com/site/about/guidelines.xhtml

> *"I had originally told my sister and she was very concerned. Then she said, 'As long as you don't stay on them.' She thought it was OK if I did it for a while but as long as I didn't stay on them. So I just sort of never told her. And she never asked."* Vallerand et al 2 pg 128

Although some seemed confident in using opioids, mostly people spoke about fears such as; addiction and uncontrolled pain. Feeling supported validated their choices and experiences and lessened some of their fears and concerns.

> *"And at the end, my partner says—we sat down there and he goes 'Stay on them.' …I've always spoke to my partner, and if he's been unsure— we've both been unsure, we've both gone into the doctor together to ask questions."* Zheng et al pg 1834

> *"my wife wanted me to take this medication. She was like: let's go for it."* Arnaert et al pg 26

5) The challenge of tapering/withdrawal from opioids

Four papers [21, 24 42, 43] explore patients' experiences of tapering or withdrawing as their main content. Two further papers [32, 33] addressed it as a more peripheral issue (see CERQual ratings in table 2). This describes the challenges and profound effects of tapering or withdrawing from opioids.

Tapering and withdrawing from opioids could be challenging and provoke anxiety.

> *"I have a tremendous fear in a doctor saying I want you to taper off the methadone and get totally off the methadone with no alternative whatsoever. I think that would be an irrational decision by a doctor, and I probably wouldn't take that advice."* Frank et al pg1842

This anxiety could be alleviated by support from a trusted health care provider or other person.

For peer review only - http://bmjopen.bmj.com/site/about/guidelines.xhtml

"The best thing about it was that nobody acted like I was a bad person because I was on these medications and was having to be going through this really slow process of coming down off of them." Frank et al pg1843

Successful tapering was described as a collaborative agreement between HCP and patient.

''She put me down to 2 and a half [pills per day]. Then she said, okay, we'll go down to half a pill. I told her I didn't think that just 2 a day would do it, and she said okay, we'll try 2 and a half, are you agreeable with that? I said that's fine. I mean, we can discuss stuff. It doesn't have to be a disagreement because we can talk about it. It's not an argument. We're 2 adults having a conversation, figuring out what to do.'' Matthias et al 2017 pg 1369

However, not all people experienced joint decision making when tapering

''I just don't feel that he's understanding. he don't seem to care what I'm saying, because he's lowering it down anyway, even though I've told him…that I didn't agree with it being lowered.'' Matthias et al 2017 pg 1369

For those in the USA, prescribing policies, advising clinicians to monitor and decrease opioid use, and the legislation to enforce these policies made those taking opioids feel as if they were 'a public health problem'. This could have a negative effect on the doctor patient relationship and leave the patient feeling disempowered. This was compounded when opioids had been withdrawn by legislation.[21, 42]

"I have to struggle, suffer, to make the next the next time that I can get my medicine. And I don't think that's fair to me because if I can take my medicine a little more regularly , I would be able to do more……I don't think that the law, people, politicians, or anybody should be able to tell anybody that's in pain what type of medicine they can take." Al Achkar et al pg 7

For peer review only - http://bmjopen.bmj.com/site/about/guidelines.xhtml

"That kinda got me mad, cause I thought well you know. . .they're taking it off the market because of people abusing it. . .It's not fair to us, you know. . . . I think the government was wrong to. . . pull them off the market, you know, because of people abusing them, no like they weren't looking at the people that need them. . .But I think it's really unfair that people that really do need them can't get them." Chang and Ibrahim 2017 pg 3

Overarching theme: Constantly balancing

After considering the fives themes, an overarching theme emerged - 'Constant balancing'. The theme *Reluctant users with little choice* describes the need to balance the pros and cons of starting opioids and the need to balance having pain with their hesitancy to use opioids.

"I don't really like being on a lot of tablets, I've never been a tablet person, um. . . but I mean I can't have the pain either so it's one evil outdoing the other evil. Paterson et al pg 723

Studies describe balancing the dose for pain management with their side effects to allow them to function. Participants constantly weighed up the effects on their life; dealing with an internal conflict of unresolved pain versus necessary medication, being opioid free versus having uncontrolled pain and balancing other stressors against opioid dose changes.

"If you're going to be able to walk, and you take one pain pill so you can walk and live life, you're going to do it, even though you may not like it." Penney et al pg 6

The theme *Stigma feeling scared and secretive but needing support*, describes the need to balance their hopes for relief with fear of side effects, and also to balance

For peer review only - http://bmjopen.bmj.com/site/about/guidelines.xhtml

whether or not to disclose their opioid use with the risk of being labelled a 'drug seeker' versus having unrelieved pain.

> "*I do it for my own protection by not telling them because I see how they react by reading something in the paper…and it's just their ignorance. And I don't have time. Well they know what's going on but they don't get it to this day. So you have to pick your battles…"* Brooks et al pg 19

The theme *Understanding opioids; the good and the bad*, showed people had different levels of understanding but weighed up their decisions and trade-offs against their pain relief.

> *"It's, it's got a good and bad side, morphine. ……When I take it, it works really, really well but it makes you feel rather sick, umm, rather spaced out and thinking wise, umm, it outcomes more on the other, do I want to be sick or do I want to cry with pain? So I'd rather be sick but it is a very, very good painkiller. "* Blake et al pg 105

The therapeutic alliance theme showed that often it was evident that they were 'not on the same page' with them balancing the advice from their doctors with what they wanted.

> *"[My provider] said you could die any time, and my husband and I said, well, we realize that, but because of the pain, you know, we were willing to take that risk that I would die from the narcotic medication."* Frank et al pg 1841

It also meant that there were multiple barriers to the process of decreasing opioids due to this constant balancing act which is described in the theme *the challenge of tapering/withdrawal from opioids*.

For peer review only - http://bmjopen.bmj.com/site/about/guidelines.xhtml

"I will tell her, if I do come off this medication, there are going to be consequences. I can't walk as often, I can't stand as long, I just can't do it...." Vallerand et al 1 pg 169

Discussion

Our five themes were; 1**)** Reluctant users with little choice; 2) Understanding opioids: the good the bad; 3) A therapeutic alliance: not always on the same page; 4**)** Stigma: feeling scared and secretive but needing support; and 5) The challenge of tapering or withdrawal. An overarching theme of 'constantly balancing' emerged from the data. These themes all had positive and negative aspects although the negative were more prevalent by far.

We present a line of argument of how complex it is for the patient to balance decisions at every stage of their journey. First their reluctance to start taking opioids but feeling they had no option. Patients are given opioids for CNMP often as a last resort when all other treatment has failed and their lives are so profoundly affected that they talk of a desperation, that they would literally 'try anything'. Patients spoke about not being given any detailed information about opioids and that they had learned more about them over time from different sources. This varied understanding about opioids and their side effects can affect the decisions that people make. Patients reported the need to keep the dosage of opioids as low as possible and often that they were not at risk of addiction or overdose if they were taking them as prescribed. Even those who felt they may be addicted sometimes viewed this as an acceptable trade-off for short term pain relief. Our findings indicate that patient desperation combined with inadequate information from healthcare professionals could trigger the prescription of opioids. It may be that delivering accurate information about the

For peer review only - http://bmjopen.bmj.com/site/about/guidelines.xhtml

potential side effects and limited efficacy of opioids for chronic pain management would reduce the use of opioids.

Our findings demonstrate that the stigma surrounding how patients feel about being on opioids can be compounded by the judgements of others. Although patients often describe themselves in terms of 'reluctant users', if they experienced the benefits of opioids through decreased pain and thus increased function they are often too scared to reduce opioids and return to a life of potentially unmanaged pain.

Our findings suggest that clinicians and patients with chronic pain are not always 'on the same page'. The theme *Therapeutic alliance* captures the positives, but also the tensions and mismatches of perceptions held by healthcare providers who are attempting to limit dose escalation, and patients who may view constant dose escalation as an acceptable trade-off for reducing relentless pain. The therapeutic alliance is a robust theme supported by 26 of the 31 studies included. This is not surprising as patients rely on their health care professionals to prescribe opioids. This finding resonates with qualitative evidence syntheses (QES) exploring the experience of patients [11] and healthcare professionals [46] It seems clear that joint decision -making is important for appropriate healthcare; however, our findings suggests that there are instances of mistrust on both sides. A QES exploring clinicians experience of prescribing opioids for chronic pain demonstrate that the process of prescribing opioids is not straightforward for clinicians who face a complex decision - 'Should I shouldn't I' prescribe opioids for chronic non-malignant pain [46]. They also demonstrate that clinicians must walk a fine line to balance the pros and cons of opioids whilst also maintaining patient trust. This suggests that both patients and

For peer review only - http://bmjopen.bmj.com/site/about/guidelines.xhtml

HCPs find dealing with prescribing/taking opioids for CNMP is complex and involves balancing and trade-offs.

Current guidance from Royal College of Anaesthetists in the UK and The Centers for Disease Control and Prevention in the US advocate a preference for non-opioid therapies in the treatment of CNMP[47]. If a clinician feels that opioids are indicated, then they recommend a low dose for a short duration which should be assessed for effectiveness and regularly evaluated for benefits and harms. All but four studies in this review are between 2005 and 2017, prior to these guidelines. Although opioid contracts in some areas of the USA and Canada can make patients feel stigmatised and judged, this effect can be mediated by a good therapeutic relationship. Some physicians may view contracts as necessary to guard against uncontrolled dose escalation, repeated demands for replacement of lost or misplaced medication, subversion and illicit opioid intake. This finding resonates with Toye et al (2017) who describe the moral boundary work and social guardianship that clinicians associate with opioid prescription. Our findings suggest that this role does may not contribute to an effective therapeutic partnership.

Limitations of this study

A majority of the studies are from the United States and the findings need to be taken in the context of its health and social care systems. Most of the articles in this qualitative synthesis were published or the research was conducted, before the impact of the opioid epidemic became clear to regulators and the medical profession. Further evidence is needed to find out if these themes are universal for developed countries or whether there are important differences.

Our conceptual framework highlights patients need to constantly balance and to consider the pros and cons of taking opioids. This can have a profound effect on

For peer review only - http://bmjopen.bmj.com/site/about/guidelines.xhtml

peoples' relationships with their family, friends and health care providers and their perceived standing in the community which is reflected in their careful balancing of disclosure. The therapeutic alliance and having a clear understanding of all the positive and negative aspects of opioids were important factors that underpinned their ability to maintain this fragile balance. This balance might also affect a person's desire or ability to taper or withdraw from opioids.

The GRADE-CERQual ratings (table 2) revealed we had confidence in the findings with only a few minor concerns and no moderate or serious concerns.

Conclusions and recommendations for future research

The first meta-ethnography on this topic revealed a constant balancing and a life in flux in an effort to maintain participation in life and relationships. These are important features of opioid use for CNMP. To maintain this delicate balance they often need support from family or clinicians, however this balance can be upset by the feeling of being judged by this same potential support system or peers and society at large through the media. The therapeutic alliance with healthcare professionals, the extent of people's understanding as well as the stigma attached to opioid use need to be navigated by people who are often reluctant to be on opioids in the first place.

Authors Contribution

VN and KS contributed to the review concept and design as part of the I-WOTCH process evaluation team. HS, SE, MU and KS were involved in the design of the IWOTCH study. VN, KS and FT screened search results or extracted data, conducted the analysis and synthesis. All authors contributed to data interpretation, revised the final manuscript critically for important intellectual content and appraised the final manuscript. VN prepared the final manuscript and will be the corresponding author.

For peer review only - http://bmjopen.bmj.com/site/about/guidelines.xhtml

Funding

This project was funded by the National Institute for Health Research, Health Technology Assessment (project number 14/224/04). The views and opinions expressed therein are those of the authors and do not necessarily reflect those of the HTA, NIHR, NHS or the Department of Health. Ethics approval was provided by Yorkshire and the Humber South Yorkshire Research Ethics committee on 13-9-16.

Declaration of competing interests

KS has undertaken other meta-ethnographies and was on the NIHR HS&DR Board until January 2018. The authors declared no potential conflicts of interest with respect to the research, authorship, and/or publication of this article.

SE is grant holder on a number of NIHR studies including being Co- chief investigator of the I-WOTCH study, he has prescribed opioids as part of his pain practice.

MU was Chair of the NICE accreditation advisory committee until March 2017 for which he received a fee. He is chief investigator or co-investigator on multiple previous and current research grants from the UK National Institute for Health Research, Arthritis Research UK and is a co-investigator on grants funded by the Australian NHMRC. He is an NIHR Senior Investigator. He has received travel expenses for speaking at conferences from the professional organisations hosting the conferences. He is a director and shareholder of Clinvivo Ltd that provides electronic data collection for health services research. He is part of an academic partnership with Serco Ltd related to return to work initiatives. He is a co-investigator on a study receiving support in kind from Stryker Ltd. He has accepted honoraria for teaching

For peer review only

from CARTA He is an editor of the NIHR journal series, and a member of the NIHR Journal Editors Group, for which he receives a fee.

Data Sharing Statement: No additional data are available

Acknowledgements

We would like to thank Samantha Johnson an academic librarian who helped with the electronic searches and Dr Stephanie Tierney who helped to screen the citations.

References

1. Breivik H, Collett B, Ventafridda V, et al. Survey of chronic pain in Europe: prevalence, impact on daily life, and treatment. *Eur J Pain*2006;10:287-333.
2. Nahin RL. Estimates of pain prevalence and severity in adults: United States, 2012. *J Pain*2015;16:769-80.
3. Els C, Jackson TD, Kunyk D, et al. Adverse events associated with medium- and long-term use of opioids for chronic non-cancer pain: an overview of Cochrane Reviews. *Cochrane Database of Systematic Reviews*2017.
4. Gomes T, Tadrous M, Mamdani MM, et al. The burden of opioid-related mortality in the united states. *JAMA Network Open*2018;1:e180217.
5. Furlan AD, Sandoval JA, Mailis-Gagnon A, et al. Opioids for chronic noncancer pain: a meta-analysis of effectiveness and side effects. *CMAJ*2006;174:1589-94.
6. Busse JW, Wang L, Kamaleldin M, et al. Opioids for Chronic Noncancer Pain: A Systematic Review and Meta-analysis. *JAMA*2018;320:2448-60.
7. Noblit GW and Hare RD. Meta-ethnography: Synthesizing qualitative studies. sage, 1988.
8. France EF, Cunningham M, Ring N, et al. Improving reporting of meta-ethnography: The eMERGe reporting guidance. *J Adv Nurs*2019;75:1126-39.
9. Manchikanti L, Helm S, 2nd, Fellows B, et al. Opioid epidemic in the United States. *Pain Physician*2012;15:ES9-38.
10. Critical Appraisal Skills Programme (CASP). Oxford: Public Health Resource Unit, 2006.
11. Toye F, Seers K and Barker KL. Meta-ethnography to understand healthcare professionals' experience of treating adults with chronic non-malignant pain. *BMJ Open*2017;7:e018411.
12. Lewin S, Booth A, Glenton C, et al. Applying GRADE-CERQual to qualitative evidence synthesis findings: introduction to the series. *Implement Sci*2018;13:2.
13. Lewin S, Bohren M, Rashidian A, et al. Applying GRADE-CERQual to qualitative evidence synthesis findings-paper 2: how to make an overall CERQual

For peer review only - http://bmjopen.bmj.com/site/about/guidelines.xhtml

assessment of confidence and create a Summary of Qualitative Findings table. *Implement Sci*2018;13:10.
14. Schutz AJCP. Collected Papers. Vol. 1. The Hague: Martinus Nijhoff.. 1964. 1962;2.
15. Arnaert A and Ciccotosto G. Response phases in methadone treatment for chronic nonmalignant pain. *Pain Management Nursing*2006;7:23-30.
16. Bergman AA, Matthias MS, Coffing JM, et al. Contrasting tensions between patients and PCPs in chronic pain management: a qualitative study. *Pain Medicine*2013;14:1689-97.
17. Blake S, Ruel B, Seamark C, et al. Experiences of patients requiring strong opioid drugs for chronic non-cancer pain: a patient-initiated study. *British Journal of General Practice*2007;57:101-8.
18. Brooks EA, Unruh A and Lynch ME. Exploring the lived experience of adults using prescription opioids to manage chronic noncancer pain. *Pain Research & Management*2015;20:15-22.
19. Buchbinder M, Wilbur R, McLean S, et al. "Is there any way I can get something for my pain?" Patient strategies for requesting analgesics. *Patient Education and Counseling*2015;98:137-43.
20. Chang Y-P, Wray LO, Sessanna L, et al. Use of prescription opioid medication among community-dwelling older adults with noncancer chronic pain. *Journal of Addictions Nursing (Taylor & Francis Ltd)*2011;22:19-24.
21. Chang F and Ibrahim S. Perceptions of community-dwelling patients and their physicians on OxyContin ® discontinuation and the impact on chronic pain management. *Pain Research & Management*2017;2017:11.
22. Coyne KS, Currie BM, Holmes WC, et al. Assessment of a Stool Symptom Screener and Understanding the Opioid-Induced Constipation Symptom Experience. *Patient*2015;8:317-27.
23. Esquibel AY and Borkan J. Doctors and patients in pain: Conflict and collaboration in opioid prescription in primary care. *Pain*2014;155:2575-82.
24. Frank JW, Levy C, Matlock DD, et al. Patients' Perspectives on Tapering of Chronic Opioid Therapy: A Qualitative Study. *Pain Medicine*2016;17:1838-47.
25. Green TC, Case P, Fiske H, et al. Perpetuating stigma or reducing risk ? Perspectives from naloxone consumers and pharmacists on pharmacy-based naloxone in 2 states. *Journal of the American Pharmacists Association*2017;57:S19-+.
26. Hooten WM, Vickers KS, Shi Y, et al. Smoking cessation and chronic pain: patient and pain medicine physician attitudes. *Pain Practice*2011;11:552-63.
27. Krebs EE, Bergman AA, Coffing JM, et al. Barriers to guideline-concordant opioid management in primary care-a qualitative study. *Journal of Pain*2014;15:1148-55.
28. Matthias MS, Krebs EE, Bergman AA, et al. Communicating about opioids for chronic pain: A qualitative study of patient attributions and the influence of the patient–physician relationship. *European Journal of Pain*2014;18:835-43.
29. McCrorie C, Closs SJ, House A, et al. Understanding long-term opioid prescribing for non-cancer pain in primary care: a qualitative study. *BMC Family Practice*2015;16:121.
30. Mueller SR, Koester S, Glanz JM, et al. Attitudes Toward Naloxone Prescribing in Clinical Settings: A Qualitative Study of Patients Prescribed High Dose Opioids for Chronic Non-Cancer Pain. *Journal of General Internal Medicine*2017;32:277-83.

For peer review only - http://bmjopen.bmj.com/site/about/guidelines.xhtml

31. Paterson C, Ledgerwood K, Arnold C, et al. Resisting Prescribed Opioids: A Qualitative Study of Decision Making in Patients Taking Opioids for Chronic Noncancer Pain. *Pain Medicine*2016;17:717-27.
32. Penney LS, Ritenbaugh C, DeBar LL, et al. Provider and patient perspectives on opioids and alternative treatments for managing chronic pain: a qualitative study. *BMC Family Practice*2017;17.
33. Rieb LM, Norman WV, Martin RE, et al. Withdrawal-associated injury site pain (WISP): A descriptive case series of an opioid cessation phenomenon. *Pain*2016;157:2865-74.
34. Simmonds MJ, Finley EP, Vale S, et al. A qualitative study of veterans on long-term opioid analgesics: barriers and facilitators to multimodality pain management. *Pain Medicine*2015;16:726-32.
35. St Marie B. Primary care experiences of people who live with chronic pain and receive opioids to manage pain: A qualitative methodology. *Journal of the American Association of Nurse Practitioners*2016;28:429-35.
36. Vallerand A and Nowak L. Chronic Opioid Therapy for Nonmalignant Pain: The Patient's Perspective. Part I-Life Before and After Opioid Therapy. *Pain Management Nursing*2009;10:165-72.
37. Vallerand A and Nowak L. Chronic opioid therapy for nonmalignant pain: the patient's perspective. Part II--Barriers to chronic opioid therapy. *Pain Management Nursing*2010;11:126-31.
38. Wallace LS, Wexler RK, McDougle L, et al. Voices that may not otherwise be heard: A qualitative exploration into the perspectives of primary care patients living with chronic pain. *Journal of Pain Research*2014;7:291-99.
39. Warms CA, Marshall HM, Hoffman AJ, et al. There are a few things you did not ask about my pain: writing on the margins of a survey questionnaire. *Rehabilitation Nursing*2005;30:248-56.
40. Zgierska AE, Burzinski CA, Cox J, et al. Mindfulness Meditation-Based Intervention Is Feasible, Acceptable, and Safe for Chronic Low Back Pain Requiring Long-Term Daily Opioid Therapy. *Journal of Alternative & Complementary Medicine*2016;22:610-20.
41. Zheng Z, Paterson C, Ledgerwood K, et al. Chaos to hope: a narrative of healing. *Pain Medicine*2013;14:1826-38.
42. Al Achkar M, Revere D, Dennis B, et al. Exploring perceptions and experiences of patients who have chronic pain as state prescription opioid policies change: a qualitative study in Indiana. *BMJ open*2017;7:e015083.
43. Matthias MS, Johnson NL, Shields CG, et al. "I'm Not Gonna Pull the Rug out From Under You": Patient-Provider Communication About Opioid Tapering. *Journal of Pain*2017;18:1365-73.
44. Matthias MS, Donaldson MT, Jensen AC, et al. "I Was a Little Surprised": Qualitative Insights From Patients Enrolled in a 12-Month Trial Comparing Opioids With Nonopioid Medications for Chronic Musculoskeletal Pain. *Journal of Pain*2018;19:1082-90.
45. Smith CL, Severtsen B, Vandermause R, et al. Seeking Chronic Pain Relief: A Hermeneutic Exploration. *Pain Management Nursing*2018.
46. Toye F, Seers K, Tierney S, et al. A qualitative evidence synthesis to explore healthcare professionals' experience of prescribing opioids to adults with chronic non-malignant pain. *BMC Fam Pract*2017;18:94.
47. CDC. CDC Guideline for Prescribing Opioids for Chronic Pain: (2019, accessed 12-4-19).

For peer review only - http://bmjopen.bmj.com/site/about/guidelines.xhtml

For peer review only

For peer review only - http://bmjopen.bmj.com/site/about/guidelines.xhtml

Figure 1 Concept model of the experiences of people taking opioid medication for chronic non-malignant pain

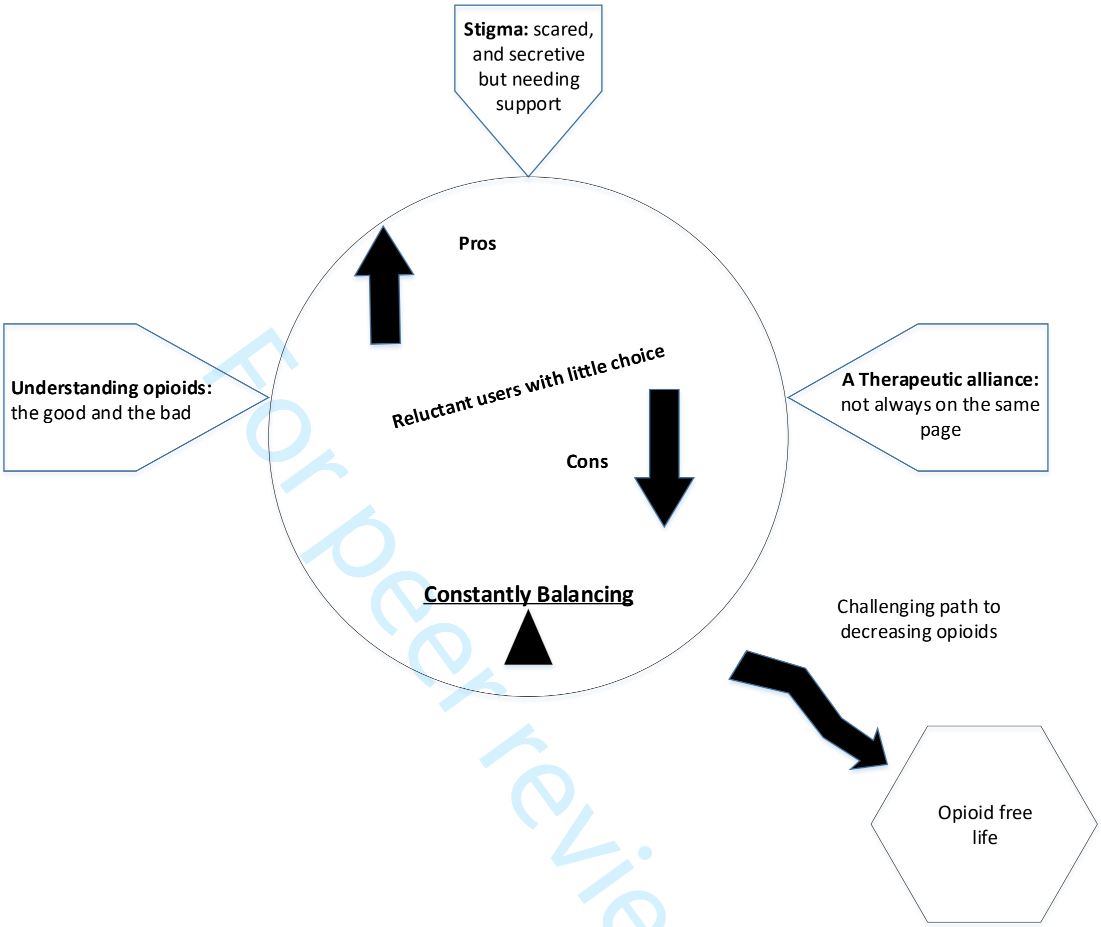

For peer review only

Figure 2: PRISMA Flow Diagram

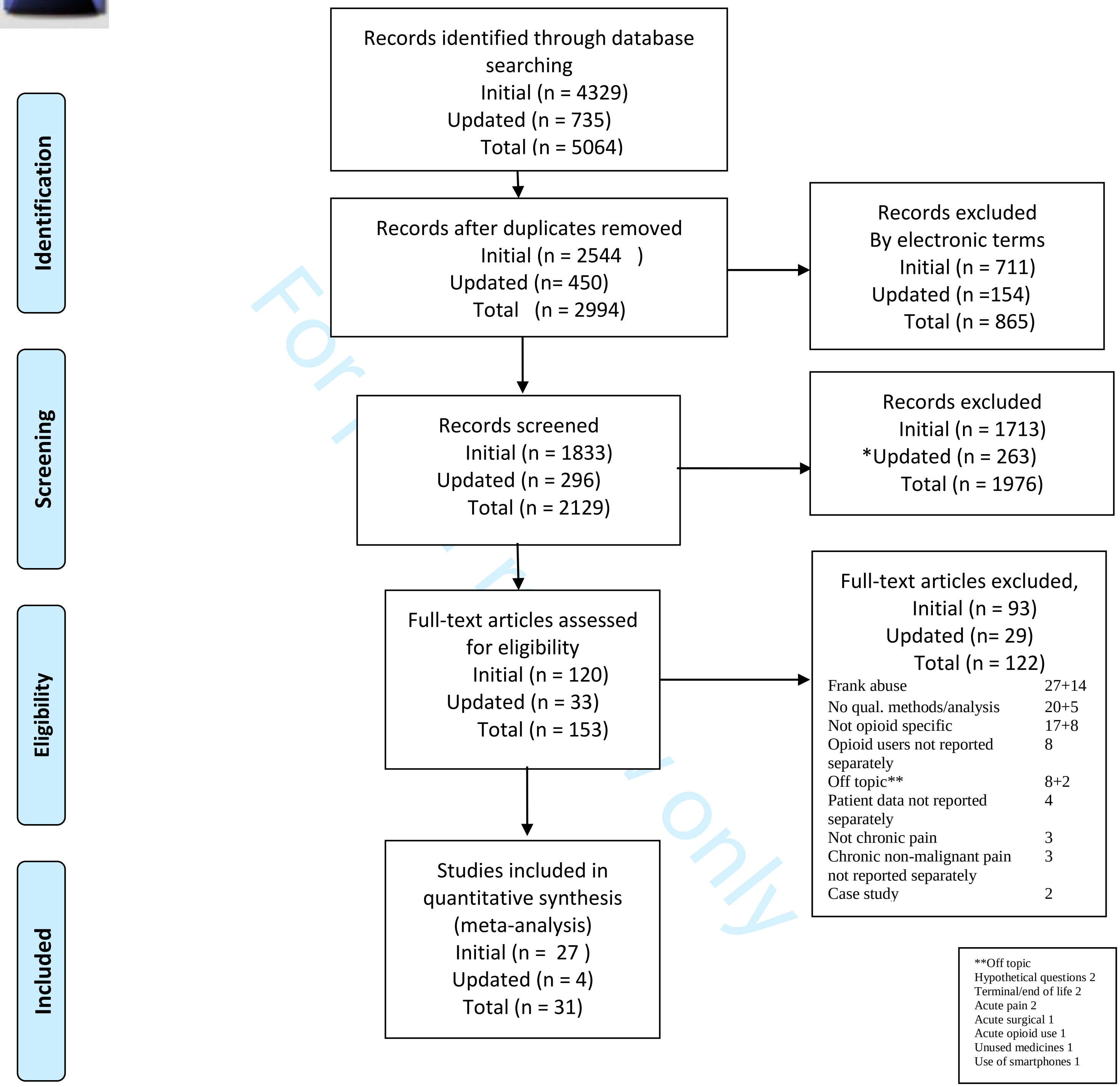

For peer review only

Initial search June 2017 and updated search Sept 2018 reported.

The search dates for the rerun could be specified in some databases, but not all. Therefore some papers were found in both searches. These twice found duplicate papers were removed at this point*

From: Moher D, Liberati A, Tetzlaff J, Altman DG, The PRISMA Group (2009). *P*referred *R*eporting *I*tems for *S*ystematic Reviews and *M*eta-*A*nalyses: The PRISMA Statement. PLoS Med 6(7): e1000097. doi:10.1371/journal.pmed1000097

Appendix 1 The eMERGe meta-ethnography reporting guidance

Numbered criteria headings with explanatory reporting criteria	Page
**Phase 1—Selecting meta-ethnography and getting started**	
Introduction<br>1 Rationale and context for the meta-ethnography<br>Describe the gap in research or knowledge to be filled by the meta-ethnography, and the wider context of the meta-ethnography	4
2 Aim(s) of the meta-ethnography<br>Describe the meta-ethnography aim(s)	4
3 Focus of the meta-ethnography<br>Describe the meta-ethnography review question(s) (or objectives)	4
4 Rationale for using meta-ethnography<br>Explain why meta-ethnography was considered the most appropriate qualitative synthesis methodology	4
**Phase 2—Deciding what is relevant**	
Methods<br>5 Search strategy<br>Describe the rationale for the literature search strategy	4/5
6 Search processes<br>Describe how the literature searching was carried out and by whom	4/5
7 Selecting primary studies<br>Describe the process of study screening and selection, and who was involved	5
Findings<br>8 Outcome of study selection<br>Describe the results of study searches and screening	16
**Phase 3—Reading included studies**	
Methods<br>9 Reading and data extraction approach<br>Describe the reading and data extraction method and processes	6
Findings<br>10 Presenting characteristics of included studies<br>Describe characteristics of the included studies	Table1
**Phase 4—Determining how studies are related**	
Methods<br>11 Process for determining how studies are related<br>Describe the methods and processes for determining how the included studies are related: - Which aspects of studies were compared AND- How the studies were compared	6
Findings<br>12 Outcome of relating studies<br>Describe how studies relate to each other	17
**Phase 5—Translating studies into one another**	16
Methods<br>13 Process of translating studies<br>Describe the methods of translation: - Describe steps taken to preserve the context and meaning of the relationships between concepts within and across studies- Describe how the reciprocal and refutational translations were conducted- Describe how potential alternative interpretations or explanations were considered in the translations	16
Findings<br>14 Outcome of translation<br>Describe the interpretive findings of the translation.	17 to 28
**Phase 6—Synthesizing translations**	
Methods<br>15 Synthesis process<br>Describe the methods used to develop overarching concepts (“synthesised translations”)Describe how potential alternative interpretations or explanations were considered in the synthesis	16
Findings<br>16 Outcome of synthesis process<br>Describe the new theory, conceptual framework, model, configuration, or interpretation of data developed from the synthesis	Figure 1
**Phase 7—Expressing the synthesis**	

For peer review only

Discussion<br>17 Summary of findings<br>Summarize the main interpretive findings of the translation and synthesis and compare them to existing literature	28 to 30
18 Strengths, limitations, and reflexivity<br>Reflect on and describe the strengths and limitations of the synthesis:<br>- Methodological aspects—for example,describe how the synthesis Findings were influenced by the nature of the included studies and how the meta-ethnography was conducted.<br>- Reflexivity—for example, the impact of the research team on the synthesis findings	30
19 Recommendations and conclusions<br>Describe the implications of the Synthesis	31

Reference: France et al. BMC Medical Research Methodology (2019) 19:25
https://doi.org/10.1186/s12874-018-0600-0

For peer review only

Appendix 2 – example of search terms

Scopus<br>((TITLE-ABS-KEY(buprenorphine or fentanyl or heroin or hydromorphone or methadone or morphine or opium or oxycodone or pentazocine or tramadol or opiate* or opioid*) OR TITLE-ABS-KEY(suboxone or bunavail or zubsolv or dipipanone or diconal or wellconal or diamorphine or duragesic or fentora or actiq or abstral or recivit or effentora or instanyl or pecfent or oxycontin or roxicodone or oramorph or papaveretum or omnopon or fortral)OR TITLE-ABS-KEY(sosegon or "talwin nx" or pethidine or meperidine or demerol or tapentadol or nucynta or palexia or tapal or ultram or zytram))) and ((TITLE-ABS-KEY(qualitative w/5 (theor* or study or studies or research or analysis)) OR TITLE-ABS-KEY(ethno* or emic or etic or phenomenolog* or hermeneutic* or heidegger* or husserl* or colaizzi* or giorgi* or glaser or strauss or (van and kaam*) or (van and manen) or ricoeur or spiegelberg* or merleau) OR TITLE-ABS-KEY(constant w/3 compar*) OR TITLE-ABS-KEY(focus w/3 group*) OR TITLE-ABS-KEY(grounded w/3 (theor* or study or studies or research or analysis)) OR TITLE-ABS-KEY(narrative w/3 analysis) OR TITLE-ABS-KEY(discourse w/3 analysis) OR TITLE-ABS-KEY((lived or life) w/3 experience*) OR TITLE-ABS-KEY((theoretical or purposive) w/3 sampl*) OR TITLE-ABS-KEY("field note*" or "field record*" or fieldnote*) OR TITLE-ABS-KEY(participant* w/3 observ*) OR TITLE-ABS-KEY("action research") OR TITLE-ABS-KEY("digital adj record*" or audiorecord* or taperecord* or videorecord* or videotap*) OR TITLE-ABS-KEY(cooperative and inquir*) OR TITLE-ABS-KEY(co and operative and inquir*) OR TITLE-ABS-KEY(co-operative and inquir*) OR TITLE-ABS-KEY(("semi-structured" or semistructured or unstructured or structured) w/3 interview*) OR TITLE-ABS-KEY((Informal or in-depth or indepth or "in depth") w/3 interview*) OR TITLE-ABS-KEY(("face-to-face" or "face to face") w/3 interview*) OR TITLE-ABS-KEY("ipa" or "interpretive phenomenological analysis") OR TITLE-ABS-KEY(social and construct*) OR TITLE-ABS-KEY("appreciative inquiry") OR TITLE-ABS-KEY(poststructural* or "post structural*" or post-structural*) OR TITLE-ABS-KEY(postmodern* or "post modern*" or post-modern*) OR TITLE-ABS-KEY(feminis*) OR TITLE-ABS-KEY(humanistic or existential or experiential))) and (TITLE-ABS-KEY(pain))

For peer review only

For peer review only

Appendix 2 Critical Appraisal Skills Programme (CASP) Quality appraisal tool scores

Study	Q1	Q2	Q3	Q4	Q5	Q6	Q7	Q8	Q9	Q10	Score
**Arnaert 2006**	Yes	Yes	Yes	Yes	?	No	Yes	Yes	Yes	Yes	17/20
**Bennett 2013**	Yes	Yes	Yes	Yes	Yes	No	Yes	?	Yes	Yes	17/20
**Bergman 2013**	Yes	Yes	Yes	Yes	Yes	No	Yes	?	Yes	Yes	17/20
**Blake 2007**	Yes	Yes	Yes	Yes	Yes	?	Yes	Yes	Yes	Yes	19/20
**Brooks 2015**	Yes	Yes	Yes	Yes	Yes	No	Yes	?	Yes	Yes	17/20
**Buchbinder 2015**	Yes	Yes	Yes	Yes	Yes	No	Yes	Yes	Yes	Yes	18/20
**Chang F 2017**	Yes	Yes	Yes	Yes	Yes	No	?	?	Yes	Yes	16/20
**Chang Y-P 2011**	Yes	Yes	Yes	Yes	Yes	No	Yes	?	?	Yes	16/20
**Coyne 2015**	Yes	Yes	Yes	Yes	Yes	No	Yes	?	Yes	Yes	17/20
**Esquibel 2012**	Yes	Yes	Yes	Yes	Yes	Yes	Yes	Yes	Yes	Yes	20/20
**Frank 2016**	Yes	Yes	Yes	Yes	Yes	Yes	Yes	Yes	Yes	Yes	20/20
**Green 2017**	Yes	Yes	Yes	Yes	Yes	No	Yes	Yes	Yes	Yes	18/20
**Hooten 2011**	Yes	Yes	Yes	Yes	Yes	No	Yes	Yes	Yes	Yes	18/20
**Krebs 2014**	Yes	Yes	Yes	Yes	Yes	?	Yes	Yes	Yes	Yes	19/20
**Lewis 2014**	Yes	Yes	Yes	?	Yes	?	Yes	Yes	Yes	Yes	18/20
**Matthias 2013**	Yes	Yes	Yes	Yes	Yes	No	Yes	Yes	Yes	Yes	18/20
**McCrorie**	Yes	Yes	?	Yes	Yes	No	Yes	Yes	Yes	Yes	17/20
**Mueller**	Yes	Yes	Yes	Yes	?	No	Yes	Yes	?	Yes	16/20
**Paterson 2016**	Yes	Yes	Yes	Yes	Yes	?	?	Yes	Yes	Yes	18/20
**Penney 2016**	Yes	Yes	?	?	?	?	Yes	?	?	?	13/20
**Rieb 2016**	Yes	Yes	Yes	Yes	Yes	?	Yes	?	Yes	Yes	18/20
**Simmonds 2015**	Yes	Yes	?	Yes	Yes	?	Yes	Yes	?	?	16/20
**St Marie 2016**	Yes	Yes	Yes	Yes	Yes	?	Yes	Yes	Yes	Yes	19/20
**Vallerand 1 2009**	Yes	Yes	Yes	Yes	Yes	No	No	Yes	?	Yes	15/20
**Vallerand 2 2010**	Yes	Yes	Yes	?	Yes	No	No	Yes	?	?	13/20
**Wallace 2014**	Yes	Yes	Yes	Yes	Yes	No	Yes	?	?	Yes	16/20
**Warms 2005**	Yes	Yes	Yes	Yes	Yes	No	No	Yes	Yes	Yes	16/20
**Zgierska 2016**	Yes	Yes	Yes	Yes	Yes	No	Yes	Yes	Yes	Yes	18/20
**Zheng 2013**	Yes	Yes	Yes	Yes	Yes	No	?	Yes	Yes	Yes	17/20
**Al Achkar 2017**	Yes	Yes	Yes	Yes	Yes	No	Yes	Yes	Yes	Yes	18/20
**Matthias 2017**	Yes	Yes	Yes	Yes	Yes	No	Yes	Yes	Yes	Yes	18/20
**Matthias 2018**	Yes	Yes	Yes	Yes	Yes	?	Yes	Yes	Yes	Yes	19/20
**Smith 2018**	Yes	Yes	Yes	Yes	Yes	Yes	Yes	Yes	Yes	Yes	20/20

Legend 1 Critical Appraisal Skills Programme (CASP) questions scoring: Yes =2 ? (Can't Tell) = 1 No = 0

Q1. Was there a clear statement of the aims of the research?
Q2. Is a qualitative methodology appropriate?
Q3. Was the research design appropriate to the aims of the research?
Q4. Was the recruitment strategy appropriate to the aims of the research?
Q5. Was the data collected in a way that addressed the research issue?
Q6. Has the relationship between researcher and participants been adequately considered?
Q7. Have ethical issues been taken into consideration?
Q8. Was the data analysis sufficiently rigorous?
Q9. Is there a clear statement of findings?
Q10. How valuable is the research?

For peer review only

BMJ Open

A systematic review and qualitative evidence synthesis of the experiences of people taking opioid medication for chronic non-malignant pain: a meta-ethnography

Journal:	*BMJ Open*
Manuscript ID	bmjopen-2019-032988.R1
Article Type:	Original research
Date Submitted by the Author:	29-Oct-2019
Complete List of Authors:	Nichols, Vivien; University of Warwick, Warwick Clinical Trials Unit, Warwick Medical School<br>Toye, Francine; Oxford University Hospitals NHS Trust, Physiotherapy Research Unit, Nuffield Orthopaedic Centre<br>Eldabe, Sam; The James Cook University Hospital<br>Sandhu, Harbinder; University of Warwick, Warwick Clinical Trials Unit, Warwick Medical School<br>Underwood, Martin; University of Warwick, Warwick Clinical Trials Unit, Warwick Medical School<br>Seers, Kate; University of Warwick, Warwick Research in Nursing, Division of Health Sciences, Warwick Medical School
**Primary Subject Heading**:	Qualitative research
Secondary Subject Heading:	Patient-centred medicine
Keywords:	opioid, patients' views, QUALITATIVE RESEARCH, chronic non-malignant pain, meta-ethnography, qualitative evidence synthesis

SCHOLARONE™
Manuscripts

I, the Submitting Author has the right to grant and does grant on behalf of all authors of the Work (as defined in the below author licence), an exclusive licence and/or a non-exclusive licence for contributions from authors who are: i) UK Crown employees; ii) where BMJ has agreed a CC-BY licence shall apply, and/or iii) in accordance with the terms applicable for US Federal Government officers or employees acting as part of their official duties; on a worldwide, perpetual, irrevocable, royalty-free basis to BMJ Publishing Group Ltd ("BMJ") its licensees and where the relevant Journal is co-owned by BMJ to the co-owners of the Journal, to publish the Work in this journal and any other BMJ products and to exploit all rights, as set out in our licence.

The Submitting Author accepts and understands that any supply made under these terms is made by BMJ to the Submitting Author unless you are acting as an employee on behalf of your employer or a postgraduate student of an affiliated institution which is paying any applicable article publishing charge ("APC") for Open Access articles. Where the Submitting Author wishes to make the Work available on an Open Access basis (and intends to pay the relevant APC), the terms of reuse of such Open Access shall be governed by a Creative Commons licence – details of these licences and which Creative Commons licence will apply to this Work are set out in our licence referred to above.

Other than as permitted in any relevant BMJ Author's Self Archiving Policies, I confirm this Work has not been accepted for publication elsewhere, is not being considered for publication elsewhere and does not duplicate material already published. I confirm all authors consent to publication of this Work and authorise the granting of this licence.

For peer review only

A systematic review and qualitative evidence synthesis of the experiences of people taking opioid medication for chronic non-malignant pain: a meta-ethnography

Mrs Vivien Nichols (1) Dr Francine Toye (2) Professor Sam Eldabe (3) Dr Harbinder Sandhu (1) Professor Martin Underwood (1,) and Professor Kate Seers (4)

(1) Warwick Clinical Trials Unit
Warwick Medical School
University of Warwick, UK

(2) Physiotherapy Research Unit, Nuffield Orthopaedic Centre,
Oxford University Hospitals NHS Foundation Trust
Oxford, UK

(3) Academic Pain Department
The James Cook University Hospital
Middlesbrough, UK

(4) Warwick Research in Nursing
Warwick Medical School
University of Warwick, UK

Corresponding author:
Mrs Vivien Nichols
Warwick Clinical Trials Unit,
Warwick Medical School,
University of Warwick
Coventry CV4 7AL
Tel: 02476574652
Email: V.P.Nichols@warwick.ac.uk

For peer review only - http://bmjopen.bmj.com/site/about/guidelines.xhtml

Abstract

Objective To review qualitative studies of the experience of taking opioid medication for Chronic Non-Malignant Pain (CNMP) or coming off them.

Design A qualitative evidence synthesis using meta-ethnography. We used a seven step approach from the methods of meta-ethnography.

Data sources and eligibility criteria We searched selected databases for qualitative studies which gave patients' views of taking opioid medication for CNMP or of coming off them.

Data extraction and synthesis Papers were quality appraised using a Critical Appraisal Skills Programme (CASP) tool and Grading of Recommendations Assessment, Development and Evaluation working group - Confidence in Evidence from Reviews of Qualitative research) (GRADE-CERQual) guidelines were applied. We identified concepts and iteratively abstracted these concepts into a line of argument.

Results We screened 2129 unique citations, checked 153 full texts and 31 met our review criteria. We identified five themes: 1) Reluctant users with little choice; 2) Understanding opioids: the good and the bad; 3) A therapeutic alliance: not always on the same page; 4) Stigma: feeling scared and secretive but needing support; and 5) The challenge of tapering or withdrawal. A new overarching theme of 'constantly balancing' emerged from the data.

Conclusions People taking opioids are constantly balancing tensions, not always wanting to take opioids, but feeling they have no choice because of the pain. They frequently feel judged, were not always 'on the same page' as their health care professional and changes in opioid use were often challenging.

For peer review only

Key words: Opioid, patients' views, qualitative research, chronic non-malignant pain, meta-ethnography, qualitative evidence synthesis.

Word count 4,940

Strengths and Limitations

- To our knowledge this is the first qualitative evidence synthesis of patients' experiences of taking opioid medications.
- Meta-ethnography provides a thorough, systematic way of synthesising qualitative findings across multiple studies and gives the reviewer's interpretation of the data.
- Using a GRADE-CERQual approach can assist in rating confidence in the review findings.
- Qualitative research that illuminates patients' perspectives can help to shape future approaches to opioid management.

Introduction

Chronic non-malignant pain (CNMP) affects between an estimated 11% and 20% of the population in Europe and US and can impact heavily on people's quality of life [1, 2]. Opioid medications are strong painkillers which have a well-established role in the treatment of acute and cancer pain; they have also been advocated for CNMP. Opioids can have distressing side effects as dosages increase such as; constipation, sedation, drowsiness, nausea, decreased concentration and memory, or mood changes [3]. Most people who use opioids develop tolerance to the painkilling effect of opioids, and

For peer review only - http://bmjopen.bmj.com/site/about/guidelines.xhtml

For peer review only

some become dependent on them. Studies have shown that high opioid usage can also put people's lives at risk [4]. Despite this, the prescription of opioid medication for CNMP has risen sharply in the higher income countries. Few studies of opioids have shown effectiveness beyond 12 weeks follow up. Population surveys have shown long-term use to be associated with increased side effects and limited pain relief [3 5 6].

This synthesis of qualitative research was undertaken to underpin a process evaluation for the Improving the Wellbeing of people with Opioid Treated CHronic pain (I-WOTCH) study funded by the National Institute for Health Research. I-WOTCH is a randomised controlled trial evaluating a multi-component education and patient centred group intervention with a one-to-one tapering programme against a control of an advice booklet with a relaxation CD. More information can be found in the main study protocol [7] and process evaluation protocol [8].

This qualitative evidence synthesis uses the methods of meta-ethnography to find out what peoples' experiences are of both using opioids for CNMP and their attempts to stop taking them.

Methods

We use Noblit and Hare's 7 stages of meta-ethnographic analysis [9]. We used the new eMERGe reporting guidelines for meta-ethnography to structure our report [10] (*See appendix 1).* The protocol is published in the international prospective register of systematic reviews (PROSPERO) registration number: CRD42017082418. http://www.crd.york.ac.uk/PROSPERO

Step 1 Getting Started.

In order to address what has been labelled an opioid epidemic[11], we need to understand people's experiences of being on opioids and of coming off them. Our

For peer review only - http://bmjopen.bmj.com/site/about/guidelines.xhtml

team was chosen because of its expertise in primary qualitative research and qualitative evidence synthesis specific to chronic pain and opioid prescription.

Step 2 Deciding what is relevant.

We undertook systematic electronic searches in June 2017 with a rerun in September 2018, appraising relevant papers for quality using the Critical Appraisal Skills Programme (CASP) tool for qualitative research [12]. One researcher (VN) with the assistance of an academic librarian (SJ) searched seven electronic databases; Medline, Embase, AMED, CINAHL, PsycInfo Web of Science and Scopus (Science citation index and Social Science Citation Index) and forward citation searches. We used search terms, free text and MeSH terms for all opioid drugs as well as their generic names. We combined these with the MeSH term 'pain' and a wide range of MeSH terms and words to describe all types of qualitative research and its analysis based upon a search used by Toye, Seers and Barker in 2017 [13]. The search was limited to those in English regarding humans with no cut-off date. Appendix 2 shows an example of our search terms.

Unique citations were screened independently by 2 researchers (VN ST *see acknowledgements*) against our inclusion and exclusion criteria (see Box 1). Any disagreements were arbitrated by a third researcher (KS). Papers for full text reading were identified and read by two researchers. Quality was assessed using a CASP tool. VN critically appraised the studies and KS independently appraised 10% for consistency. The CASP scores are shown in table 1 and appendix 3. The GRADE-CERQual was used to appraise the reviewers' confidence in the research findings [14, 15].

Box 1 Inclusion/Exclusion criteria

**Included Studies**<br>Adults (18 years or older) taking or have taken opioid medication in the last five years

For peer review only - http://bmjopen.bmj.com/site/about/guidelines.xhtml

Published in English in peer review journals with no time constraint
Must relate to patient perspectives on using opioid medication for chronic non-malignant pain
Must use qualitative methodology (any analytical approach) or mixed quantitative and qualitative methodology with qualitative findings reported separately
Where studies include participants with differing medication we will include studies where the experience of those taking opioids is reported separately
**Excluded studies**
Theoretical or methodological papers
Purely quantitative studies or mixed methods studies where the qualitative data are not presented separately
Studies concerning active cancer
Studies concerning headache
Studies concerning any acute, or acute postoperative, pain
Studies concerned only with heath care professional or carer perspectives, or studies of mixed carer/ patient/ professional populations where patient perspectives are not presented separately
Non- English language studies
Theses or conference abstracts which are not peer reviewed

Step 3 Reading the studies

VN read all the studies and KS and FT read half of these papers each (so all were read twice) and all extracted the second order concepts independently. A second order concept is a researcher's interpretation of data in a primary qualitative study [16]. VN, KS and FT met to discuss and reach agreement, and compiled a spreadsheet of all of the concepts extracted from the papers

Step 4 Determining how the studies are related?

VN sorted the concepts into categories by looking for any similarities and differences across all the studies. VN, KS and FT discussed the categorisation of data on multiple occasions. To enable comparison across studies, VN recorded descriptive data about each study (see table 1)

For peer review only - http://bmjopen.bmj.com/site/about/guidelines.xhtml

For peer review only

For peer review only - http://bmjopen.bmj.com/site/about/guidelines.xhtml

Table 1 Study characteristics with CASP and GRADE-CERQual relevance ratings

Studies	Country	Data collection / participants	Analytical Approach	Aims *Italics = verbatim quotes*	Morphine Equivalent Daily Dose mg/day (MED)	CASP score	Relevance
**1 Arnaert et al 2006**[17] Response Phases in Methadone Treatment for Chronic Non-malignant Pain	Canada	Semi-structured interview N=11 (4M/7F)	Content analysis	*"...to develop an understanding and to gain knowledge about the beliefs of patients with CNMP who were taking methadone, and to explore what challenges, if any, existed that affected their beliefs when first starting methadone treatment as an activity in their personal lives."*	None reported	17/20	P
**2 Bergman et al 2013** [18]Contrasting Tensions Between Patients and PCPs in Chronic Pain Management: A Qualitative Study	USA	In-depth interviews N=26 (24m/2F)	Inductive thematic analysis	*"... to develop a better understanding of the respective experiences, perceptions, and challenges both patients with chronic pain and PCPs face communicating with each other about pain management in the primary care setting."*	None reported	17/20	P
**3 Blake et al 2007**[19] Experiences of patients requiring strong opioid drugs for chronic non-cancer pain: a patient initiated study	UK	One focus group N=4 (2M/2F) and interviews N=10 (3M/5F)	Interpretative Phenomenological Analysis	*"...to determine the attitudes and experiences of patients receiving long-term strong opioid medication for chronic non-cancer pain in primary care."*	Individual opioid dosages	19/20	R
**4 Brooks et al 2015** [20]Exploring the lived experience of adults using prescription opioids to manage chronic noncancer pain	Canada	In-depth interviews N=9 (4M/5F)	Interpretative Phenomenological Analysis	*"...to explore the lived experience of adults using prescription opioids to manage CNCP, focusing on how opioid medication affected their daily lives."*	None reported	17/20	R

For peer review only - http://bmjopen.bmj.com/site/about/guidelines.xhtml

**5 Buchbinder et al 2014** [21]''Is there any way I can get something for my pain?'' Patient strategies for requesting analgesics	USA	Audio recorded clinical encounters N=74 (37M/37F)	Qualitative approach based on conversational analysis	*"We examined the direct and indirect means by which patients express a desire for analgesic medication."*	None reported	18/20	I
**6 Chang Y-P et al 2011** [22] Use of Prescription Opioid Medication among Community-Dwelling Older Adults with Noncancer Chronic Pain	USA	Face to face interview N=21 (13M/8F) ≥65yrs	Content analysis, mixed methods	*"… to: (1) describe older adults' patterns of adherence to their prescription opioid medication regimens and their reasons for these medication use patterns; and (2) examine the associations between adherence of prescription opioids, pain intensity, and pain interference on daily activity."*	None reported	16/20	P
**7 Chang,F et al 2017** [23]Perceptions of Community-Dwelling Patients and Their Physicians on OxyContin ® Discontinuation and the Impact on Chronic Pain Management	Canada	Pt interviews N=13 1M/ 12F	Not specified, no references given	*"… to explore the perceptions of patients with chronic pain and their physicians on OxyContin discontinuation and the impact that it had on chronic pain care and management."*	None reported	16/20	P
**8 Coyne et al 2015** [24] Assessment of a Stool Symptom Screener and Understanding the Opioid-Induced Constipation Symptom Experience	USA	Cognitive interview semi structured think-aloud approach N=66 (27M/39F)	Content analysis	*"…to evaluate the content validity of the Stool Symptom Screener by assessing patient understanding of its items, patient ability to differentiate among response options, and patient perceptions about the use of a 2-week recall period for evaluating stool symptoms, BMs, and laxative use.*<br>*- to evaluate how patients describe their constipation experience and to understand*	None reported	17/20	I

For peer review only - http://bmjopen.bmj.com/site/about/guidelines.xhtml

				whether this differs between patients who frequently use laxatives and those who do not."			
**9 Esquibel et al 2014** [25] Doctors and patients in pain: Conflict and collaboration in opioid prescription in primary care	USA	In depth interviews with pts with CNCP receiving opioids and their physicians. N=21 pts (8M/13F)	Immersion/ crystallization process to generate a thematic codebook. Mixed methods with some quant. data analysis	*"…to better understand the effects of COT [chronic opioid therapy] on the doctor–patient relationship."*	None reported	20/20	R
**10 Frank et al 2016** [26] Patients' Perspectives on Tapering of Chronic Opioid Therapy: A Qualitative Study	USA	Semi-structured interviews N=24 (11M/13F)	Team based, mixed inductive and deductive approach guided by the Health Belief Model	*"… to explore patients' perspectives on opioid tapering."*	MED: Used algorithm<br><br>Median (IQR) 70 (30-165) Range 15-1845	20/20	R
**11 Green et al 2017** [27] Perpetuating stigma or reducing risk? Perspectives from naloxone consumers and pharmacists on pharmacy-based naloxone in 2 states	USA	8 focus groups (only 2 with patients with chronic pain) N=15 (8M/7F)	Thematic analysis. Content analysis with a collaborative codebook development process	*"…to explore the attitudes of various stakeholders toward pharmacy-based naloxone and opioid medication safety and to capture the range of initial experiences with pharmacy naloxone in 2 states with pharmacy naloxone policies."*	None reported	18/20	P

For peer review only - http://bmjopen.bmj.com/site/about/guidelines.xhtml

**12 Hooten et al 2011** [28] Smoking Cessation and Chronic Pain: Patient and Pain Medicine Physician Attitudes	USA	N = 18 Interviews N= 15 and focus group N=3 10M/8F	Participatory research, mixed methods - Thematic and content analysis	*"...to determine the attitudes and beliefs of both patients and pain medicine physicians regarding smoking cessation interventions applied during pain therapy."*	MED - used equianalgesic conversion software programme Mean ± SD 227 ± 356	18/20	I
**13 Krebs et al 2014** [29] Barriers to Guideline-Concordant Opioid Management in Primary Care—A Qualitative Study	USA	Semi structured interviews N= 26 patients (14 PC physicians) 13M/13F	Immersion crystallization approach	*"...to better understand primary care physicians' and patients' perspectives on recommended opioid management practices and to identify potential barriers and facilitators of guideline concordant opioid management in primary care."*	None reported	16/20	P
**14 Matthias et al 2014** [30] Communicating about opioids for chronic pain: A qualitative study of patient attributions and the influence of the patient–physician relationship	USA	Audio Recordings of primary care visits and Semi-structured interviews after clinic visit. N=30 26M/4F	Immersion/crystallization approach	*"...to advance understanding about communication about opioids by directly capturing clinical communication about opioids, as well as interviewing patients to gain insight into communication patterns and their broader relationships with their physicians, and how these relationships shape communication about opioids."*	None reported	18/20	R
**15 McCrorie et al 2015**[31] Understanding long-term opioid prescribing for non-cancer pain in primary care: a qualitative study	UK	Semi structured interviews with patients (focus groups with	Grounded approach for thematic analysis. Constant comparison	*"... to understand the processes which bring about and perpetuate long-term prescribing of opioids for chronic, non-cancer pain. We interviewed patients and GPs about their experiences, beliefs and expectations of analgesia prescribing, to improve understanding of how*	None reported	17/20	R

For peer review only - http://bmjopen.bmj.com/site/about/guidelines.xhtml

		GPs) N=23 6M/17F		*problematic long-term opioid prescribing becomes established".*			
**16 Mueller et al 2016** [32] Attitudes Toward Naloxone Prescribing in Clinical Settings: A Qualitative Study of Patients Prescribed High Dose Opioids for Chronic Non-Cancer Pain	USA	Semi-stuctured interview N=24 8M/16F	Ethnographic iterative methods. Inductive and deductive approaches	*"This study assessed the knowledge and attitudes toward naloxone prescribing among non-cancer patients prescribed opioids in primary care."*	Inclusion criteria ≥100mg MED	16/20	I
**17 Paterson et al 2016** [33] Resisting Prescribed Opioids: A Qualitative Study of Decision Making in Patients Taking Opioids for Chronic Noncancer Pain	Australia	Qualitative individual interviews N=20	Constant comparison and decision making explored in depth and with a thematic analysis utilizing a published "Model of medicine-taking"	*"…to identify the varying influences on patients' decisions about their use of prescribed long-term opioids. The research questions are: 1) does this conceptual Model of medicine-taking apply to people taking prescribed opioids, 2) is the concept of "resistance" to medication useful in this context, and 3) does this concept lead to new insights which may improve communication and shared decision making between"*	None reported	18/20	P
**18 Penney et al 2016** [34] Provider and patient perspectives on opioids	USA	11 Focus groups N=80 and	Thematic coding ref	*"…to identify the practical issues patients and providers face when accessing alternatives to*	None reported	13/20	P

For peer review only - http://bmjopen.bmj.com/site/about/guidelines.xhtml

and alternative treatments for managing chronic pain: a qualitative study		individual interviews N=10 27M/63F	Ryan, Bernard 2003	*opioids, and how multiple parties view these issues."*			
**19 Rieb et al 2016** [35] Withdrawal-associated injury site pain (WISP): a descriptive case series of an opioid cessation phenomenon	Canada	In person semi structured interviews N=21 14M/7F	Deductive and inductive approaches. Use of survey and emergent interview themes	*".... to document the existence and characteristics of this pain phenomenon that we have named withdrawal-associated injury site pain (WISP)."*	Recalled dose before WISP	18/20	P
**20 Simmonds et al 2015** [36] A Qualitative Study of Veterans on Long-Term Opioid Analgesics: Barriers and Facilitators to Multimodality Pain Management	USA	Semi structured focus groups x 3 N=25 17M/8F	Grounded theory-informed approach. Framework provided by the theory of planned behaviour	*"...to examine barriers and facilitators to multimodality chronic pain care among veterans on high-dose opioid analgesics for chronic non-cancer pain."*	Inclusion criteria at least 50 mg MED	16/20	P
**21 St Marie et al 2016** [37] Primary care experiences of people who live with chronic pain and receive opioids to manage pain: A qualitative methodology	USA	Semistructured interviews. N=12 6M/6F	Thematic and interpretive analyses	*"...to provide a deeper understanding of the experiences, issues, and challenges that people face who live with chronic pain and received opioids to help manage their pain in primary care. The following research questions were addressed: (a) What are the experiences of individuals who live with chronic pain and receive opioid pain medications to manage their pain in*	None reported	19/20	R

For peer review only - http://bmjopen.bmj.com/site/about/guidelines.xhtml

				primary care? (b) What have been their healthcare experiences as they strive to manage their pain?"			
**22 Vallerand et al 1 2009** [38] Chronic Opioid Therapy for Nonmalignant Pain: The Patient's Perspective. Part I— Life Before and After Opioid Therapy	USA	In depth serial interviews N=22 6M/16F	Phenomenologic study / Constant comparative method	*"…to examine the lived experience of adults receiving opioid therapy for relief of chronic non-malignant pain through the examination of data obtained through serial taper recorded interviews."*	Range 22.5 to 3,200	14/20	R
**23 Vallerand et al 2 2010** [39] Chronic Opioid Therapy for Non-malignant Pain: The Patient's Perspective. Part II— Barriers to Chronic Opioid Therapy	USA	In depth serial narrative interviews N=22 6M/16F	Phenomenologic study / Constant comparative method	*"…to elucidate the essence of the lived experience of patients receiving chronic opioid therapy for chronic non-malignant pain through examining their life narratives."*	None reported	13/20	R
**24 Wallace et al 2014** [40] Voices that may not otherwise be heard: a qualitative exploration into the perspectives of primary care patients living with chronic pain	USA	Photovoice photos, Interviews 1st N=31 2nd N=25 , and focus groups N=19	Grounded theory	*"…this study examined the utility of a combination of qualitative methods (Photovoice, one-on-one interviews, and focus groups) in examining the daily experiences of primary care patients living with chronic pain"*.	None reported	16/20	R
**25 Warms et al 2005** [41] There are a few things you did not ask about my pain: writing on the margins of a survey	USA	Comments written in the margins of a questionnaire N=797	Content analysis	*"…to determine the characteristics of those who wrote comments* [written in the margin of survey questionnaire] *and to understand what was being communicated in their comments."*	None reported	16/20	P

For peer review only

**26 Zgierska et al 2016** [42] Mindfulness Meditation-Based Intervention Is Feasible, Acceptable, and Safe for Chronic Low Back Pain Requiring Long-Term Daily Opioid Therapy	USA	Qual data on treatment satisfaction and experience N= 17	Qualitative analysis methods. Grounded theory	*"... to determine feasibility, acceptability, and safety of an MM-based intervention in patients with CLBP requiring daily opioid therapy."*	Inclusion criteria ≥30mg MED<br><br>Mean ± SD 166.9 ± 153.7	18/20	I
**27 Zheng et al 2013** [43]Chaos to Hope: A Narrative of Healing	Australi a	In depth qual interviews (N=20) 10M/10F	Illness narratives. Thematic analysis	*"...to investigate the progression of the illness and opioid journeys of people who are taking opioids for chronic non-cancer pain".*	None reported	17/20	P
**Rerun of search**							
**28 Al Achkar et al 2017** [44] Exploring perceptions and experiences of patients who have chronic pain as state prescription opioid policies change: a qualitative study in Indiana	USA	N=9 3M/6F	Inductive emergent thematic analysis	*"...to evaluate the impact of Indiana's opioid prescription legislation on the patient experiences around pain management."*	None reported	18/20	R
**29 Matthias et al 2017** [45] "I'm Not Gonna Pull the Rug out From Under You": Patient-Provider	USA	N=37 12M/25F	Inductive approach, constant comparison	*"...to understand communication processes related to opioid tapering."*	None reported	18/20	R

For peer review only - http://bmjopen.bmj.com/site/about/guidelines.xhtml

Communication About Opioid Tapering							
**30 Matthias et al 2018** [46] "I Was a Little Surprised": Qualitative Insights From Patients Enrolled in a 12-Month Trial Comparing Opioids With Nonopioid Medications for Chronic Musculoskeletal Pain	USA	N=34 28M/6F	Inductive approach, constant comparison	*"…to understand patients' experiences with the SPACE trial including their views and anticipated benefits of opioids, versus non -opioids, experiences with the intervention and to what extent expectations were met after completing the study."*	None reported	19/20	R
**31 Smith et al 2018** [47] Seeking Chronic Pain Relief: A Hermeneutic Exploration	USA	N=15 4M/11F	Concurrent embedded mixed methods design, Heideggerian hermeneutic phenomenological approach	*"This research presents an interpretation of the experience of seeking pain relief for a group of people taking opioid pain medications whose pain is not adequately controlled"*	None reported	20/20	P

Legend: GRADE-CERQual Relevance component: R= Relevant, P= Partial relevance, I= Indirect relevance, U=Uncertain relevance

IQR = Interquartile Range, SD = Standard Deviation

For peer review only - http://bmjopen.bmj.com/site/about/guidelines.xhtml

Step 5 Translating studies into each other

Patterns and associations between categories were explored and all researchers felt that a line of argument approach as defined by Noblit and Hare [9] would be the most useful method to interpret the data.

Step 6 Synthesising Translations

Agreement was reached by clearly defining the over-arching or 3rd order concepts arising from the data. A third order concept is the reviewers' interpretation of second order concepts.

Step 7 Expressing the synthesis

We developed a conceptual model to show how the themes related to each other in a line of argument. (see figure 1)

Insert figure 1 about here.

Patient and Public Involvement

We did not involve patients or the public in our work.

Results

Two reviewers VN and ST screened 2994 titles or abstracts (after the removal of duplicates from the 5064 citations retrieved) and identified 153 full texts of interest. Two reviewers VN and KS read these and 122 were excluded. Reasons are given in the PRISMA flowchart, see figure 2. The reviewers agreed to include 31 studies. The included studies were from US (23), Canada (4) UK (2), and Australia (2) and used a range of qualitative methods.

We report the 4 facets of GRADE-CERQual for all papers. 1) Methodological 2) Confidence 3) Relevance 4) Adequacy of data - See table 2 below.

For peer review only - http://bmjopen.bmj.com/site/about/guidelines.xhtml

Table 2 Confidence in review findings: GRADE-CERQual assessment

Review findings	Studies contributing (see table 1 column 1 for study number)	Methodological limitations (study number)	Relevance (See table 1 end column)	Coherence	Adequacy of data
Reluctant users with little choice	1,3,4,5,6,7,17,18,21,22,26,27,30 (13 studies)	11 no concerns<br>2 minor concerns (18,22)	5 Relevant<br>6 Partial<br>2 Indirect	No concerns	No concerns
Understanding opioids: the good the bad	1,3,7,9,10,11,15, 16,17, 23,25, 27,29. (13 studies)	12 no concerns<br>1 minor concerns (23)	6 Relevant<br>6 Partial<br>1 Indirect	No concerns	No concerns
A therapeutic alliance: not always on the same page	1,2,3,4,5,7,9,10, 11,13,14,15,16, 17,18,19,20,21, 22,23,24,25,26, 28,29,31 (26 studies)	23 no concerns<br>3 minor concerns (18,22,23)	12 Relevant<br>11 Partial<br>3 Indirect	No concerns	No concerns
Stigma: feeling scared, and secretive but needing support	1,2,3,4,7,9,10,14,16,17,18,20,21, 22, 23, 24, 27, 28, 31 (19 studies)	16 no concerns<br>3 minor concerns (18,22,23)	10 Relevant<br>8 Partial<br>1 Indirect	No concerns	No concerns
The challenge of tapering/ withdrawal from opioids	7,10,18,19,30,31 (6 studies)	5 no concerns<br>1 minor concerns (18)	2 Relevant<br>4 Partial	Minor concerns	Minor concerns

Legend of GRADE-CERQual component scoring:

Methodological limitations, Coherence, Adequacy of data	No or very minor concerns<br>Minor concerns<br>Moderate concerns<br>Serious concerns
Relevance:	Relevant<br>Partial<br>Indirect<br>Uncertain

For peer review only

Synthesis of Findings

We abstracted five themes from the 2nd order concepts. Table 3 below shows how each study contributed to each theme. We have illustrated each concept with exemplary quotations.

Table 3 Themes apparent in each study

Author date	RU	U	TA	S	TW
1 Arnaert and Ciccotosto 2006	X	X	X	X	

2 Bergman, Matthias et al 2013			X	X	
3 Blake, Ruel et al 2007	X	X	X	X	
4 Brooks, Unruh et al 2015	X		X	X	
5 Buchbinder, Wilbur et al 2014	X		X		
6 Chang Y-P, Wray et al 2011	X				
7 Chang,F and Ibrahim,S 2017	X	X	X	X	X
8 Coyne, Currie et al 2015					
9 Esquibel and Borkan 2014		X	X	X	
10 Frank, Levy et al 2016		X	X	X	X
11 Green, Case et al 2017		X	X		
12 Hooten, Vickers et al 2011					
13 Krebs, Bergman et al 2014			X		
14 Matthias,Krebs et al 2013			X	X	
15 McCrorie, Closs et al 2015		X	X		
16 Mueller, Koester et al 2016		X	X	X	
17 Paterson, Ledgerwood 2016	X	X	X	X	
18 Penney, Ritenbaugh et al 2016	X		X	X	X
19 Rieb, Norman et al 2016			X		X
20 Simmonds, Finley et al 2015			X	X	
21 St Marie et al 2015	X		X	X	
22 Vallerand et al 1 2009	X		X	X	
23 Vallerand et al 2 2010		X	X	X	
24 Wallace et al 2014			X	X	
25 Warms et al 2005 o		X	X		
26 Zgierska et al 2016	X		X		
27 Zheng et al 2013	X	X		X	
**Studies from search rerun**	**RU**	**U**	**TA**	**S**	**TW**
28 Al Achkar et al 2017			X	X	X
29 Matthias et al 2017		X	X		X
30 Matthias et al 2018	X				
31 Smith et al 2018			X	X	

Legend:
RU = Reluctant users with little choice
U = Understanding about opioids: the good and the bad
TA = A therapeutic alliance: not always on the same page
S = Stigma: feeling scared, and secretive but needing support
TW = The challenges of tapering or withdrawal
X = theme present in paper

For peer review only

1) Reluctant users with little choice

This describes a resistance or hesitancy to take opioids mainly due to concerns about side effects or addiction, although they felt there were no other options available.

> *"I don't want to become addicted, if I'm going to become addicted then as far as I'm concerned I'm a druggie, so I might as well not be here anyway, so I don't want to become addicted…"* Blake et al Pg103

For peer review only - http://bmjopen.bmj.com/site/about/guidelines.xhtml

"I just didn't want to go on them because I mean once you get on them that's it, you're sort of stuck on them. I didn't want to take morphine at first because there was a girl that I went through one of the courses with and she always seemed really dopey and drugged up so it took them a long while to talk me to into taking the morphine because I didn't want to be like that.

Zheng et al pg 1832

Some spoke of underusing or were keen to reduce their medications when possible. There was a dislike of being on long term medication and some thought that it would not relieve their pain.

"I don't want to do that [take more morphine]. *I want to stay on as little as I possibly can because there might come a time when I need more and I don't want to be on high doses. I've always tried to keep it at a minimum amount of tablets each day…"* Blake et al Pg 105

Even though some were reluctant there were other instances of dramatic improvement in people's lives. This then weighted their choice to stay on the opioids.

*"I mean it is just like a miracle as far as I am concerned. It is like knowing it [*the pain*] is there but you have the instruments to prevent it from getting out and [be]coming a roaring demon."* Vallerand et al 1pg 170

"But opiates, that's my way of life. There would be no life if I didn't have this. And I thank God for them because without them I'd be…well I wouldn't be. I just couldn't go on. I would have committed suicide a long time ago. And I say that truthfully cause you could not live like that, with that constant, constant pain. But, with the opiates it's made it possible to be able to have a part of a life, you know." Brooks et al pg 20

For peer review only - http://bmjopen.bmj.com/site/about/guidelines.xhtml

2) Understanding opioids: the good and the bad

This describes patients' knowledge or understanding about opioids which had generally been acquired ad hoc and slowly over time, from pharmacists, patient package inserts in their medication, leaflets, the internet, television programmes and from doctors, especially doctors at the pain clinic.

> *"When you see it in the media, when you see it on the television, you think if you're taking regular morphine you must be in a pretty bad way, you know."* Blake et al Pg103

> *"I always ask before I go on a medication, what are the side effects, I was told I may experience constipation; nothing else was explained to me."* Paterson et al pg 721

There was often poor knowledge about using opioids for chronic pain, and about addiction, overdose risk and side effects.

> *"There's not too much education about it [overdose]… When I first started taking it [the opioid medication], no one told me about OD[overdose]or anything about that. Because I was taking it not [as] prescribed…I was just like when I felt pain I would just take like five or six of them or whatever. Then at the end, I'd run out."* Mueller et al pg 279

Patients often had to defend their usage and this added to their stress especially when they felt their healthcare professionals lacked an understanding of the place for opioids in the treatment of CNMP or were cautious about using them.

> *"The concern is that if they increase my opioid dosage, I could stop breathing. It's ridiculous."* Frank et al Pg1841

> *''There are still a lot of doctors out there that are against it. They think it is bad. Bad medicine. Bad practice.''* Vallerand et al 2 pg 129

For peer review only - http://bmjopen.bmj.com/site/about/guidelines.xhtml

For peer review only

In contrast, some people felt well informed which either produced more concern or gave the patients confidence in their opioid regime.

> *"… and from what I've read up, because I like to, sort of, keep on top of things, that it's an opium based drug, so you will build up some tolerance and you will build up [becomes tearful] And you will potentially become sort of addicted to it, if you like."* McCrorie et al pg 3
>
> *"Under Dr A [pain clinic] I've learnt more. And my concern has been, well it was initially the possibility of addiction, but she has assured me that I'm not showing no signs of addiction at all. I may have some withdrawal problems"* Paterson et al Pg 721

3) A therapeutic alliance: not always on the same page

This describes a therapeutic alliance or the relationship between patients and their health care providers which was considered important.

Overall there was a feeling that HCPs and patients were often 'not on the same page' about opioid usage.

> *"My family doctor…does not want me to be dependent on heavy pain meds, so I am intensely miserable 99% of the time."* Warms et al pg 252

Some patients felt they were not listened to and were frustrated by a lack of empathy from physicians regarding their pain experience.

> *"I frequently have difficulty with the residents (doctors in training) explaining why these drugs, this many drugs…Finally Dr. [family physician] wrote a note in my file – stop harassing [participant's name]. This is what she gets and why she gets it. And they did stop but it was inconvenient. For instance, they would not prescribe me three months at a time. I would be dispensed one month at a*

For peer review only - http://bmjopen.bmj.com/site/about/guidelines.xhtml

time. And for someone who had been taking the same drugs for 10 years I found that condescending. " Brooks et al pg 18

A reluctance to prescribe from GPs and pharmacists and the use of opioid contracts or a restriction of medication were often considered punitive.

"It kind of made me feel like I was doing something wrong, which I wasn't, but I signed a contract. You know, what would I be without my meds?" Krebs et al pg 1152

"And I told my doctor that, that I wanted so I could sleep through the night. And now he, well, I'll give you 10, but it's got to last. Like he treats me like a drug addict." Penney et al pg 6

The healthcare system often worked against a therapeutic alliance with lack of continuity or care or frequent visits which fed into mistrust. Patients complained that provider turnover affected their ability to receive individualised care; conversations about pain and treatment options often had to be started over again from scratch.

"*I don't have the same doctor long enough to know*". Bergman et al pg 1693

However having blood or urine tests for levels of opioids and regular checks were seen by some as being cared for.

"*I would say, 'I have this agreement and you don't have to sign it if you don't want to, but I would like to go over it with you. These are suggestions because this medication is addictive, it is dangerous, and I just want to make sure you're aware.' I think if you really want to make it where people are not hostile, say they have to have a urine test every 6 months, everybody, and that 'it's a policy because we care about all of you.'"* Krebs et al pg 1152

Some talked of the need for good relationships built on trust, shared decision making and knowledgeable specialists who communicate well.

For peer review only - http://bmjopen.bmj.com/site/about/guidelines.xhtml

"I wouldn't say I researched it to that depth, you know, I read a little bit about, and asked a lot of questions at my doctor, and then we decided." Paterson pg 722

4) Stigma: feeling scared and secretive but needing support

This describes feelings of stigma and fear which people expressed directly in relation to their opioid usage. This includes peoples' negative attitudes from family, medical professionals and work colleagues which lead to them feeling stigmatised and judged for taking opioids.

"So I'm constantly trying to clean up because I think people are going to judge me. 'Oh, because she's on all this medication, ooh, she can't look after her children.'" Paterson et al pg 724

"As soon as you mention to someone that you are on pain medication it's, 'Oh my god, you've got to get off it.' It is viewed as weak. Somehow I am weak for being on this medication." Vallerand et al 2 pg 128

To protect themselves some chose to keep their opioids a secret.

"But you know, after 2 years of pain, you are physically exhausted, mentally exhausted and depressed. So, I take my medication and I hide it at the bottom of my drawer. It's my secret life. It's always a secret, and I've got to hide it and not tell anyone." Vallerand et al 1 pg 169

Some people made a conscious decision about who they could tell and who they couldn't due to negative reactions. Relationships suffered when patients felt unsupported.

"My son told me I was a drug addict. He did. He really did. He was to the point, he didn't know what he could do for me. It really was that bad." Vallerand et al 2 pg 128

For peer review only - http://bmjopen.bmj.com/site/about/guidelines.xhtml

"I had originally told my sister and she was very concerned. Then she said, 'As long as you don't stay on them.' She thought it was OK if I did it for a while but as long as I didn't stay on them. So I just sort of never told her. And she never asked." Vallerand et al 2 pg 128

Although some seemed confident in using opioids, mostly people spoke about fears such as; addiction and uncontrolled pain. Feeling supported validated their choices and experiences and lessened some of their fears and concerns.

"And at the end, my partner says—we sat down there and he goes 'Stay on them.' …I've always spoke to my partner, and if he's been unsure— we've both been unsure, we've both gone into the doctor together to ask questions." Zheng et al pg 1834

"my wife wanted me to take this medication. She was like: let's go for it." Arnaert et al pg 26

5) The challenge of tapering/withdrawal from opioids

Four papers [23, 26 44, 45] explore patients' experiences of tapering or withdrawing as their main content. Two further papers [34, 35] addressed it as a more peripheral issue (see CERQual ratings in table 2). This describes the challenges and profound effects of tapering or withdrawing from opioids.

Tapering and withdrawing from opioids could be challenging and provoke anxiety.

"I have a tremendous fear in a doctor saying I want you to taper off the methadone and get totally off the methadone with no alternative whatsoever. I think that would be an irrational decision by a doctor, and I probably wouldn't take that advice." Frank et al pg1842

This anxiety could be alleviated by support from a trusted health care provider or other person.

For peer review only - http://bmjopen.bmj.com/site/about/guidelines.xhtml

"The best thing about it was that nobody acted like I was a bad person because I was on these medications and was having to be going through this really slow process of coming down off of them." Frank et al pg1843

Successful tapering was described as a collaborative agreement between HCP and patient.

''She put me down to 2 and a half [pills per day]. Then she said, okay, we'll go down to half a pill. I told her I didn't think that just 2 a day would do it, and she said okay, we'll try 2 and a half, are you agreeable with that? I said that's fine. I mean, we can discuss stuff. It doesn't have to be a disagreement because we can talk about it. It's not an argument. We're 2 adults having a conversation, figuring out what to do.'' Matthias et al 2017 pg 1369

However, not all people experienced joint decision making when tapering

''I just don't feel that he's understanding. he don't seem to care what I'm saying, because he's lowering it down anyway, even though I've told him…that I didn't agree with it being lowered.'' Matthias et al 2017 pg 1369

For those in the USA, prescribing policies, advising clinicians to monitor and decrease opioid use, and the legislation to enforce these policies made those taking opioids feel as if they were 'a public health problem'. This could have a negative effect on the doctor patient relationship and leave the patient feeling disempowered. This was compounded when opioids had been withdrawn by legislation.[23, 44]

"I have to struggle, suffer, to make the next the next time that I can get my medicine. And I don't think that's fair to me because if I can take my medicine a little more regularly , I would be able to do more……I don't think that the law, people, politicians, or anybody should be able to tell anybody that's in pain what type of medicine they can take." Al Achkar et al pg 7

For peer review only - http://bmjopen.bmj.com/site/about/guidelines.xhtml

"That kinda got me mad, cause I thought well you know. . .they're taking it off the market because of people abusing it. . .It's not fair to us, you know. . . . I think the government was wrong to. . . pull them off the market, you know, because of people abusing them, no like they weren't looking at the people that need them. . .But I think it's really unfair that people that really do need them can't get them." Chang and Ibrahim 2017 pg 3

Overarching theme: Constantly balancing

After considering the fives themes, an overarching theme emerged - 'Constant balancing'. The theme *Reluctant users with little choice* describes the need to balance the pros and cons of starting opioids and the need to balance having pain with their hesitancy to use opioids.

"I don't really like being on a lot of tablets, I've never been a tablet person, um. . . but I mean I can't have the pain either so it's one evil outdoing the other evil. Paterson et al pg 723

Studies describe balancing the dose for pain management with their side effects to allow them to function. Participants constantly weighed up the effects on their life; dealing with an internal conflict of unresolved pain versus necessary medication, being opioid free versus having uncontrolled pain and balancing other stressors against opioid dose changes.

"If you're going to be able to walk, and you take one pain pill so you can walk and live life, you're going to do it, even though you may not like it." Penney et al pg 6

The theme *Stigma feeling scared and secretive but needing support*, describes the need to balance their hopes for relief with fear of side effects, and also to balance

For peer review only - http://bmjopen.bmj.com/site/about/guidelines.xhtml

whether or not to disclose their opioid use with the risk of being labelled a 'drug seeker' versus having unrelieved pain.

> "*I do it for my own protection by not telling them because I see how they react by reading something in the paper…and it's just their ignorance. And I don't have time. Well they know what's going on but they don't get it to this day. So you have to pick your battles…"* Brooks et al pg 19

The theme *Understanding opioids; the good and the bad*, showed people had different levels of understanding but weighed up their decisions and trade-offs against their pain relief.

> *"It's, it's got a good and bad side, morphine. ……When I take it, it works really, really well but it makes you feel rather sick, umm, rather spaced out and thinking wise, umm, it outcomes more on the other, do I want to be sick or do I want to cry with pain? So I'd rather be sick but it is a very, very good painkiller. "* Blake et al pg 105

The therapeutic alliance theme showed that often it was evident that they were 'not on the same page' with them balancing the advice from their doctors with what they wanted.

> *"[My provider] said you could die any time, and my husband and I said, well, we realize that, but because of the pain, you know, we were willing to take that risk that I would die from the narcotic medication."* Frank et al pg 1841

It also meant that there were multiple barriers to the process of decreasing opioids due to this constant balancing act which is described in the theme *the challenge of tapering/withdrawal from opioids*.

For peer review only - http://bmjopen.bmj.com/site/about/guidelines.xhtml

"I will tell her, if I do come off this medication, there are going to be consequences. I can't walk as often, I can't stand as long, I just can't do it…." Vallerand et al 1 pg 169

Discussion

Our five themes were; 1) Reluctant users with little choice; 2) Understanding opioids: the good the bad; 3) A therapeutic alliance: not always on the same page; 4) Stigma: feeling scared and secretive but needing support; and 5) The challenge of tapering or withdrawal. An overarching theme of 'constantly balancing' emerged from the data. These themes all had positive and negative aspects although the negative were more prevalent by far.

We present a line of argument of how complex it is for the patient to balance decisions at every stage of their journey. First their reluctance to start taking opioids but feeling they had no option. Patients are given opioids for CNMP often as a last resort when all other treatment has failed and their lives are so profoundly affected that they talk of a desperation, that they would literally 'try anything'. Patients spoke about not being given any detailed information about opioids and that they had learned more about them over time from different sources. This varied understanding about opioids and their side effects can affect the decisions that people make. Patients reported the need to keep the dosage of opioids as low as possible and often that they were not at risk of addiction or overdose if they were taking them as prescribed. Even those who felt they may be addicted sometimes viewed this as an acceptable trade-off for pain relief. Our findings indicate that patient desperation combined with inadequate information from healthcare professionals could trigger the prescription of opioids. It may be that delivering accurate information about the potential side effects

For peer review only - http://bmjopen.bmj.com/site/about/guidelines.xhtml

and limited efficacy of opioids for chronic pain management would reduce the use of opioids.

Our findings demonstrate that the stigma surrounding how patients feel about being on opioids can be compounded by the judgements of others. Although patients often describe themselves in terms of 'reluctant users', if they experienced the benefits of opioids through decreased pain and thus increased function they are often too scared to reduce opioids and return to a life of potentially unmanaged pain.

Our findings suggest that clinicians and patients with chronic pain are not always 'on the same page'. The theme *Therapeutic alliance* captures the positives, but also the tensions and mismatches of perceptions held by healthcare providers who are attempting to limit dose escalation, and patients who may view constant dose escalation as an acceptable trade-off for reducing relentless pain. The therapeutic alliance is a robust theme supported by 26 of the 31 studies included. This is not surprising as patients rely on their health care professionals to prescribe opioids. This finding resonates with qualitative evidence syntheses (QES) exploring the experience of patients [13] and healthcare professionals [48] It seems clear that joint decision -making is important for appropriate healthcare; however, our findings suggests that there are instances of mistrust on both sides. A QES exploring clinicians experience of prescribing opioids for chronic pain demonstrate that the process of prescribing opioids is not straightforward for clinicians who face a complex decision - 'Should I shouldn't I' prescribe opioids for chronic non-malignant pain [48]. They also demonstrate that clinicians must walk a fine line to balance the pros and cons of opioids whilst also maintaining patient trust. This suggests that both patients and

For peer review only - http://bmjopen.bmj.com/site/about/guidelines.xhtml

HCPs find dealing with prescribing/taking opioids for CNMP is complex and involves balancing and trade-offs.

Current guidance from Royal College of Anaesthetists in the UK and The Centers for Disease Control and Prevention in the US advocate a preference for non-opioid therapies in the treatment of CNMP[49]. If a clinician feels that opioids are indicated, then they recommend a low dose for a short duration which should be assessed for effectiveness and regularly evaluated for benefits and harms. All but four studies in this review are between 2005 and 2017, prior to these guidelines. Opioid contracts in some areas of the USA and Canada can make patients feel stigmatised and judged, this effect can be moderated by a good therapeutic relationship, and reframing these as agreements rather than contracts[50]. Some physicians may view contracts/agreements as necessary to guard against uncontrolled dose escalation, repeated demands for replacement of lost or misplaced medication, subversion and illicit opioid intake. This finding resonates with Toye et al (2017) who describe the moral boundary work and social guardianship that clinicians associate with opioid prescription. Our findings suggest that this role does may not contribute to an effective therapeutic partnership.

Limitations of this study

A majority of the studies are from the United States and the findings need to be taken in the context of its health and social care systems. Most of the articles in this qualitative synthesis were published or the research was conducted, before the impact of the opioid epidemic became clear to regulators and the medical profession. Some papers discuss using opioids as a last resort, although the opioid epidemic, especially in the US suggests they are not always given as a last resort. We acknowledge that our interpretation of the data might have been influenced by the current, much more

For peer review only - http://bmjopen.bmj.com/site/about/guidelines.xhtml

critical perception of opioid use for chronic non-malignant pain. Further evidence is needed to find out if these themes are universal for developed countries or whether there are important differences.

Our conceptual framework highlights patients need to constantly balance and to consider the pros and cons of taking opioids. This can have a profound effect on peoples' relationships with their family, friends and health care providers and their perceived standing in the community which is reflected in their careful balancing of disclosure. The therapeutic alliance and having a clear understanding of all the positive and negative aspects of opioids were important factors that underpinned their ability to maintain this fragile balance. This balance might also affect a person's desire or ability to taper or withdraw from opioids.

The GRADE-CERQual ratings (table 2) revealed we had confidence in the findings with only a few minor concerns and no moderate or serious concerns.

Conclusions and recommendations for future research

The first meta-ethnography on this topic revealed a constant balancing and a life in flux in an effort to maintain participation in life and relationships. These are important features of opioid use for CNMP. To maintain this delicate balance they often need support from family or clinicians, however this balance can be upset by the feeling of being judged by this same potential support system or peers and society at large through the media. The therapeutic alliance with healthcare professionals, the extent of people's understanding as well as the stigma attached to opioid use need to be navigated by people who are often reluctant to be on opioids in the first place.

For peer review only - http://bmjopen.bmj.com/site/about/guidelines.xhtml

Authors Contribution

VN and KS contributed to the review concept and design as part of the I-WOTCH process evaluation team. HS, SE, MU and KS were involved in the design of the IWOTCH study. VN, KS and FT screened search results or extracted data, conducted the analysis and synthesis. All authors contributed to data interpretation, revised the final manuscript critically for important intellectual content and appraised the final manuscript. VN prepared the final manuscript and will be the corresponding author.

Funding

This project was funded by the National Institute for Health Research, Health Technology Assessment (project number 14/224/04). The views and opinions expressed therein are those of the authors and do not necessarily reflect those of the HTA, NIHR, NHS or the Department of Health. Ethics approval was provided by Yorkshire and the Humber South Yorkshire Research Ethics committee on 13-9-16.

Declaration of competing interests

KS has undertaken other meta-ethnographies and was on the NIHR HS&DR Board until January 2018.

SE is investigator on a number of NIHR and industry sponsored studies. He received travel expenses for speaking at conferences from the professional organisations. SE consults for Medtronic, Abbott, Boston Scientific and Mainstay Medical, none in relation to opioids. SE is chair of the BPS Science and Research Committee. SE is deputy Chair of the NIHR CRN Anaesthesia Pain and Perioperative Medicine National Specialty Group. SE's department has received fellowship funding from Medtronic as well as nurse funding from Abbott.

For peer review only - http://bmjopen.bmj.com/site/about/guidelines.xhtml

For peer review only

HS is director of Health Psychology Services Ltd, providing psychological services for a range of health related conditions.

MU was Chair of the NICE accreditation advisory committee until March 2017 for which he received a fee. He is chief investigator or co-investigator on multiple previous and current research grants from the UK National Institute for Health Research, Arthritis Research UK and is a co-investigator on grants funded by the Australian NHMRC. He is an NIHR Senior Investigator. He has received travel expenses for speaking at conferences from the professional organisations hosting the conferences. He is a director and shareholder of Clinvivo Ltd that provides electronic data collection for health services research. He is part of an academic partnership with Serco Ltd related to return to work initiatives. He is a co-investigator on a study receiving support in kind from Stryker Ltd. He has accepted honoraria for teaching from CARTA He is an editor of the NIHR journal series, and a member of the NIHR Journal Editors Group, for which he receives a fee.

Data Availability Statement: Data is available on reasonable request.

Acknowledgements

We would like to thank Samantha Johnson an academic librarian who helped with the electronic searches and Dr Stephanie Tierney who helped to screen the citations.

References

1. Breivik H, Collett B, Ventafridda V, et al. Survey of chronic pain in Europe: prevalence, impact on daily life, and treatment. *Eur J Pain*2006;10:287-333.
2. Nahin RL. Estimates of pain prevalence and severity in adults: United States, 2012. *J Pain*2015;16:769-80.
3. Els C, Jackson TD, Kunyk D, et al. Adverse events associated with medium- and long-term use of opioids for chronic non-cancer pain: an overview of Cochrane Reviews. *Cochrane Database of Systematic Reviews*2017.

For peer review only - http://bmjopen.bmj.com/site/about/guidelines.xhtml

4. Gomes T, Tadrous M, Mamdani MM, et al. The burden of opioid-related mortality in the united states. *JAMA Network Open*2018;1:e180217.
5. Furlan AD, Sandoval JA, Mailis-Gagnon A, et al. Opioids for chronic noncancer pain: a meta-analysis of effectiveness and side effects. *CMAJ*2006;174:1589-94.
6. Busse JW, Wang L, Kamaleldin M, et al. Opioids for Chronic Noncancer Pain: A Systematic Review and Meta-analysis. *JAMA*2018;320:2448-60.
7. Sandhu HK, Abraham C, Alleyne S, et al. Testing a support programme for opioid reduction for people with chronic non-malignant pain: the I-WOTCH randomised controlled trial protocol. *BMJ Open*2019;9:e028937.
8. Nichols VP, Abraham C, Eldabe S, et al. Process evaluation protocol for the I-WOTCH study: an opioid tapering support programme for people with chronic non-malignant pain. *BMJ Open*2019;9:e028998.
9. Noblit GW and Hare RD. Meta-ethnography: Synthesizing qualitative studies. sage, 1988.
10. France EF, Cunningham M, Ring N, et al. Improving reporting of meta-ethnography: The eMERGe reporting guidance. *J Adv Nurs*2019;75:1126-39.
11. Manchikanti L, Helm S, 2nd, Fellows B, et al. Opioid epidemic in the United States. *Pain Physician*2012;15:ES9-38.
12. Critical Appraisal Skills Programme (CASP). Oxford: Public Health Resource Unit, 2006.
13. Toye F, Seers K and Barker KL. Meta-ethnography to understand healthcare professionals' experience of treating adults with chronic non-malignant pain. *BMJ Open*2017;7:e018411.
14. Lewin S, Booth A, Glenton C, et al. Applying GRADE-CERQual to qualitative evidence synthesis findings: introduction to the series. *Implement Sci*2018;13:2.
15. Lewin S, Bohren M, Rashidian A, et al. Applying GRADE-CERQual to qualitative evidence synthesis findings-paper 2: how to make an overall CERQual assessment of confidence and create a Summary of Qualitative Findings table. *Implement Sci*2018;13:10.
16. Schutz AJCP. Collected Papers. Vol. 1. The Hague: Martinus Nijhoff.. 1964. 1962;2.
17. Arnaert A and Ciccotosto G. Response phases in methadone treatment for chronic nonmalignant pain. *Pain Management Nursing*2006;7:23-30.
18. Bergman AA, Matthias MS, Coffing JM, et al. Contrasting tensions between patients and PCPs in chronic pain management: a qualitative study. *Pain Medicine*2013;14:1689-97.
19. Blake S, Ruel B, Seamark C, et al. Experiences of patients requiring strong opioid drugs for chronic non-cancer pain: a patient-initiated study. *British Journal of General Practice*2007;57:101-8.
20. Brooks EA, Unruh A and Lynch ME. Exploring the lived experience of adults using prescription opioids to manage chronic noncancer pain. *Pain Research & Management*2015;20:15-22.
21. Buchbinder M, Wilbur R, McLean S, et al. "Is there any way I can get something for my pain?" Patient strategies for requesting analgesics. *Patient Education and Counseling*2015;98:137-43.
22. Chang Y-P, Wray LO, Sessanna L, et al. Use of prescription opioid medication among community-dwelling older adults with noncancer chronic pain. *Journal of Addictions Nursing (Taylor & Francis Ltd)*2011;22:19-24.

For peer review only - http://bmjopen.bmj.com/site/about/guidelines.xhtml

23. Chang F and Ibrahim S. Perceptions of community-dwelling patients and their physicians on OxyContin ® discontinuation and the impact on chronic pain management. *Pain Research & Management*2017;2017:11.
24. Coyne KS, Currie BM, Holmes WC, et al. Assessment of a Stool Symptom Screener and Understanding the Opioid-Induced Constipation Symptom Experience. *Patient*2015;8:317-27.
25. Esquibel AY and Borkan J. Doctors and patients in pain: Conflict and collaboration in opioid prescription in primary care. *Pain*2014;155:2575-82.
26. Frank JW, Levy C, Matlock DD, et al. Patients' Perspectives on Tapering of Chronic Opioid Therapy: A Qualitative Study. *Pain Medicine*2016;17:1838-47.
27. Green TC, Case P, Fiske H, et al. Perpetuating stigma or reducing risk ? Perspectives from naloxone consumers and pharmacists on pharmacy-based naloxone in 2 states. *Journal of the American Pharmacists Association*2017;57:S19-+.
28. Hooten WM, Vickers KS, Shi Y, et al. Smoking cessation and chronic pain: patient and pain medicine physician attitudes. *Pain Practice*2011;11:552-63.
29. Krebs EE, Bergman AA, Coffing JM, et al. Barriers to guideline-concordant opioid management in primary care-a qualitative study. *Journal of Pain*2014;15:1148-55.
30. Matthias MS, Krebs EE, Bergman AA, et al. Communicating about opioids for chronic pain: A qualitative study of patient attributions and the influence of the patient–physician relationship. *European Journal of Pain*2014;18:835-43.
31. McCrorie C, Closs SJ, House A, et al. Understanding long-term opioid prescribing for non-cancer pain in primary care: a qualitative study. *BMC Family Practice*2015;16:121.
32. Mueller SR, Koester S, Glanz JM, et al. Attitudes Toward Naloxone Prescribing in Clinical Settings: A Qualitative Study of Patients Prescribed High Dose Opioids for Chronic Non-Cancer Pain. *Journal of General Internal Medicine*2017;32:277-83.
33. Paterson C, Ledgerwood K, Arnold C, et al. Resisting Prescribed Opioids: A Qualitative Study of Decision Making in Patients Taking Opioids for Chronic Noncancer Pain. *Pain Medicine*2016;17:717-27.
34. Penney LS, Ritenbaugh C, DeBar LL, et al. Provider and patient perspectives on opioids and alternative treatments for managing chronic pain: a qualitative study. *BMC Family Practice*2017;17.
35. Rieb LM, Norman WV, Martin RE, et al. Withdrawal-associated injury site pain (WISP): A descriptive case series of an opioid cessation phenomenon. *Pain*2016;157:2865-74.
36. Simmonds MJ, Finley EP, Vale S, et al. A qualitative study of veterans on long-term opioid analgesics: barriers and facilitators to multimodality pain management. *Pain Medicine*2015;16:726-32.
37. St Marie B. Primary care experiences of people who live with chronic pain and receive opioids to manage pain: A qualitative methodology. *Journal of the American Association of Nurse Practitioners*2016;28:429-35.
38. Vallerand A and Nowak L. Chronic Opioid Therapy for Nonmalignant Pain: The Patient's Perspective. Part I-Life Before and After Opioid Therapy. *Pain Management Nursing*2009;10:165-72.
39. Vallerand A and Nowak L. Chronic opioid therapy for nonmalignant pain: the patient's perspective. Part II--Barriers to chronic opioid therapy. *Pain Management Nursing*2010;11:126-31.

For peer review only - http://bmjopen.bmj.com/site/about/guidelines.xhtml

40. Wallace LS, Wexler RK, McDougle L, et al. Voices that may not otherwise be heard: A qualitative exploration into the perspectives of primary care patients living with chronic pain. *Journal of Pain Research*2014;7:291-99.
41. Warms CA, Marshall HM, Hoffman AJ, et al. There are a few things you did not ask about my pain: writing on the margins of a survey questionnaire. *Rehabilitation Nursing*2005;30:248-56.
42. Zgierska AE, Burzinski CA, Cox J, et al. Mindfulness Meditation-Based Intervention Is Feasible, Acceptable, and Safe for Chronic Low Back Pain Requiring Long-Term Daily Opioid Therapy. *Journal of Alternative & Complementary Medicine*2016;22:610-20.
43. Zheng Z, Paterson C, Ledgerwood K, et al. Chaos to hope: a narrative of healing. *Pain Medicine*2013;14:1826-38.
44. Al Achkar M, Revere D, Dennis B, et al. Exploring perceptions and experiences of patients who have chronic pain as state prescription opioid policies change: a qualitative study in Indiana. *BMJ open*2017;7:e015083.
45. Matthias MS, Johnson NL, Shields CG, et al. "I'm Not Gonna Pull the Rug out From Under You": Patient-Provider Communication About Opioid Tapering. *Journal of Pain*2017;18:1365-73.
46. Matthias MS, Donaldson MT, Jensen AC, et al. "I Was a Little Surprised": Qualitative Insights From Patients Enrolled in a 12-Month Trial Comparing Opioids With Nonopioid Medications for Chronic Musculoskeletal Pain. *Journal of Pain*2018;19:1082-90.
47. Smith CL, Severtsen B, Vandermause R, et al. Seeking Chronic Pain Relief: A Hermeneutic Exploration. *Pain Management Nursing*2018.
48. Toye F, Seers K, Tierney S, et al. A qualitative evidence synthesis to explore healthcare professionals' experience of prescribing opioids to adults with chronic non-malignant pain. *BMC Fam Pract*2017;18:94.
49. CDC. CDC Guideline for Prescribing Opioids for Chronic Pain: (2019, accessed 12-4-19).
50. Tobin DG, Keough Forte K and Johnson McGee S. Breaking the pain contract: A better controlled-substance agreement for patients on chronic opioid therapy. *Cleve Clin J Med*2016;83:827-35.

For peer review only

For peer review only - http://bmjopen.bmj.com/site/about/guidelines.xhtml

Figure 1 Concept model of the experiences of people taking opioid medication for chronic non-malignant pain

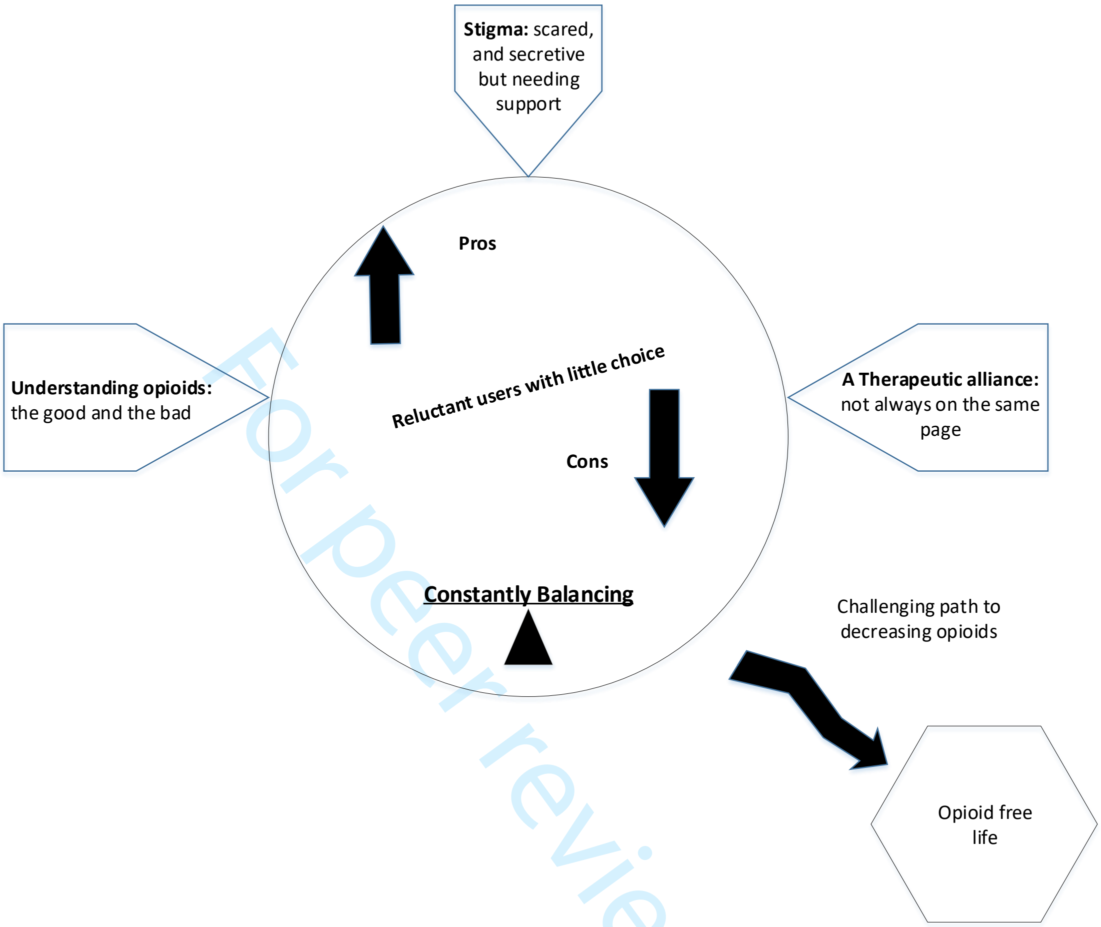

Figure 2: PRISMA Flow Diagram

Identification

Records identified through database searching
Initial (n = 4329)
Updated (n = 735)
Total (n = 5064)

Records after duplicates removed
Initial (n = 2544)
Updated (n= 450)
Total (n = 2994)

Records excluded by electronic terms
(Did not meet inclusion criteria)
Initial (n = 711)
Updated (n =154)
Total (n = 865)

Screening

Records screened
Initial (n = 1833)
Updated (n = 296)
Total (n = 2129)

Records excluded
Initial (n = 1713)
*Updated (n = 263)
Total (n = 1976)

Eligibility

Full-text articles assessed for eligibility
Initial (n = 120)
Updated (n = 33)
Total (n = 153)

Full-text articles excluded,
Initial (n = 93)
Updated (n= 29)
Total (n = 122)

Frank abuse	28+14
No qual. methods/analysis	20+5
Not opioid specific	17+8
Off topic**	11+2
Opioid users not reported separately	8
Patient data not reported separately	4
Chronic non-malignant pain not reported separately	3
Case study	2

**Off topic
Not chronic pain 3
Hypothetical questions 2
Terminal/end of life 2
Acute pain 2
Acute surgical 1
Acute opioid use 1
Unused medicines 1
Use of smartphones 1

Included

Studies included in quantitative synthesis (meta-ethnography)
Initial (n = 27)
Updated (n = 4)
Total (n = 31)

Initial search June 2017 and updated search Sept 2018 reported.
The search dates for the rerun could be specified in some databases, but not all. Therefore some papers were found in both searches. These twice found duplicate papers were removed at this point*

From: Moher D, Liberati A, Tetzlaff J, Altman DG, The PRISMA Group (2009). *P*referred *R*eporting *I*tems for *S*ystematic Reviews and *M*eta-*A*nalyses: The PRISMA Statement. PLoS Med 6(7): e1000097. doi:10.1371/journal.pmed1000097

For peer review only - http://bmjopen.bmj.com/site/about/guidelines.xhtml

Appendix 1 The eMERGe meta-ethnography reporting guidance

Numbered criteria headings with explanatory reporting criteria	Page
**Phase 1—Selecting meta-ethnography and getting started**	
Introduction<br>1 Rationale and context for the meta-ethnography<br>Describe the gap in research or knowledge to be filled by the meta-ethnography, and the wider context of the meta-ethnography	4
2 Aim(s) of the meta-ethnography<br>Describe the meta-ethnography aim(s)	4
3 Focus of the meta-ethnography<br>Describe the meta-ethnography review question(s) (or objectives)	4
4 Rationale for using meta-ethnography<br>Explain why meta-ethnography was considered the most appropriate qualitative synthesis methodology	4
**Phase 2—Deciding what is relevant**	
Methods<br>5 Search strategy<br>Describe the rationale for the literature search strategy	4/5
6 Search processes<br>Describe how the literature searching was carried out and by whom	4/5
7 Selecting primary studies<br>Describe the process of study screening and selection, and who was involved	5
Findings<br>8 Outcome of study selection<br>Describe the results of study searches and screening	16
**Phase 3—Reading included studies**	
Methods<br>9 Reading and data extraction approach<br>Describe the reading and data extraction method and processes	6
Findings<br>10 Presenting characteristics of included studies<br>Describe characteristics of the included studies	Table1
**Phase 4—Determining how studies are related**	
Methods<br>11 Process for determining how studies are related<br>Describe the methods and processes for determining how the included studies are related: - Which aspects of studies were compared AND- How the studies were compared	6
Findings<br>12 Outcome of relating studies<br>Describe how studies relate to each other	17
**Phase 5—Translating studies into one another**	16
Methods<br>13 Process of translating studies<br>Describe the methods of translation: - Describe steps taken to preserve the context and meaning of the relationships between concepts within and across studies- Describe how the reciprocal and refutational translations were conducted- Describe how potential alternative interpretations or explanations were considered in the translations	16
Findings<br>14 Outcome of translation<br>Describe the interpretive findings of the translation.	17 to 28
**Phase 6—Synthesizing translations**	
Methods<br>15 Synthesis process<br>Describe the methods used to develop overarching concepts ("synthesised translations")Describe how potential alternative interpretations or explanations were considered in the synthesis	16
Findings<br>16 Outcome of synthesis process<br>Describe the new theory, conceptual framework, model, configuration, or interpretation of data developed from the synthesis	Figure 1
**Phase 7—Expressing the synthesis**	

For peer review only

Discussion<br>17 Summary of findings<br>Summarize the main interpretive findings of the translation and synthesis and compare them to existing literature	28 to 30
18 Strengths, limitations, and reflexivity<br>Reflect on and describe the strengths and limitations of the synthesis:<br>- Methodological aspects—for example,describe how the synthesis Findings were influenced by the nature of the included studies and how the meta-ethnography was conducted.<br>- Reflexivity—for example, the impact of the research team on the synthesis findings	30
19 Recommendations and conclusions<br>Describe the implications of the Synthesis	31

Reference: France et al. BMC Medical Research Methodology (2019) 19:25
https://doi.org/10.1186/s12874-018-0600-0

For peer review only

Appendix 2 – example of search terms

Scopus<br>((TITLE-ABS-KEY(buprenorphine or fentanyl or heroin or hydromorphone or methadone or morphine or opium or oxycodone or pentazocine or tramadol or opiate* or opioid*) OR TITLE-ABS-KEY(suboxone or bunavail or zubsolv or dipipanone or diconal or wellconal or diamorphine or duragesic or fentora or actiq or abstral or recivit or effentora or instanyl or pecfent or oxycontin or roxicodone or oramorph or papaveretum or omnopon or fortral)OR TITLE-ABS-KEY(sosegon or "talwin nx" or pethidine or meperidine or demerol or tapentadol or nucynta or palexia or tapal or ultram or zytram))) and ((TITLE-ABS-KEY(qualitative w/5 (theor* or study or studies or research or analysis)) OR TITLE-ABS-KEY(ethno* or emic or etic or phenomenolog* or hermeneutic* or heidegger* or husserl* or colaizzi* or giorgi* or glaser or strauss or (van and kaam*) or (van and manen) or ricoeur or spiegelberg* or merleau) OR TITLE-ABS-KEY(constant w/3 compar*) OR TITLE-ABS-KEY(focus w/3 group*) OR TITLE-ABS-KEY(grounded w/3 (theor* or study or studies or research or analysis)) OR TITLE-ABS-KEY(narrative w/3 analysis) OR TITLE-ABS-KEY(discourse w/3 analysis) OR TITLE-ABS-KEY((lived or life) w/3 experience*) OR TITLE-ABS-KEY((theoretical or purposive) w/3 sampl*) OR TITLE-ABS-KEY("field note*" or "field record*" or fieldnote*) OR TITLE-ABS-KEY(participant* w/3 observ*) OR TITLE-ABS-KEY("action research") OR TITLE-ABS-KEY("digital adj record*" or audiorecord* or taperecord* or videorecord* or videotap*) OR TITLE-ABS-KEY(cooperative and inquir*) OR TITLE-ABS-KEY(co and operative and inquir*) OR TITLE-ABS-KEY(co-operative and inquir*) OR TITLE-ABS-KEY(("semi-structured" or semistructured or unstructured or structured) w/3 interview*) OR TITLE-ABS-KEY((Informal or in-depth or indepth or "in depth") w/3 interview*) OR TITLE-ABS-KEY(("face-to-face" or "face to face") w/3 interview*) OR TITLE-ABS-KEY("ipa" or "interpretive phenomenological analysis") OR TITLE-ABS-KEY(social and construct*) OR TITLE-ABS-KEY("appreciative inquiry") OR TITLE-ABS-KEY(poststructural* or "post structural*" or post-structural*) OR TITLE-ABS-KEY(postmodern* or "post modern*" or post-modern*) OR TITLE-ABS-KEY(feminis*) OR TITLE-ABS-KEY(humanistic or existential or experiential))) and (TITLE-ABS-KEY(pain))

For peer review only

For peer review only

Appendix 3 Critical Appraisal Skills Programme (CASP) Quality appraisal tool scores

Study	Q1	Q2	Q3	Q4	Q5	Q6	Q7	Q8	Q9	Q10	Score
**Arnaert 2006**	Yes	Yes	Yes	Yes	?	No	Yes	Yes	Yes	Yes	17/20
**Bergman 2013**	Yes	Yes	Yes	Yes	Yes	No	Yes	?	Yes	Yes	17/20
**Blake 2007**	Yes	Yes	Yes	Yes	Yes	?	Yes	Yes	Yes	Yes	19/20
**Brooks 2015**	Yes	Yes	Yes	Yes	Yes	No	Yes	?	Yes	Yes	17/20
**Buchbinder 2015**	Yes	Yes	Yes	Yes	Yes	No	Yes	Yes	Yes	Yes	18/20
**Chang F 2017**	Yes	Yes	Yes	Yes	Yes	No	?	?	Yes	Yes	16/20
**Chang Y-P 2011**	Yes	Yes	Yes	Yes	Yes	No	Yes	?	?	Yes	16/20
**Coyne 2015**	Yes	Yes	Yes	Yes	Yes	No	Yes	?	Yes	Yes	17/20
**Esquibel 2014**	Yes	Yes	Yes	Yes	Yes	Yes	Yes	Yes	Yes	Yes	20/20
**Frank 2016**	Yes	Yes	Yes	Yes	Yes	Yes	Yes	Yes	Yes	Yes	20/20
**Green 2017**	Yes	Yes	Yes	Yes	Yes	No	Yes	Yes	Yes	Yes	18/20
**Hooten 2011**	Yes	Yes	Yes	Yes	Yes	No	Yes	Yes	Yes	Yes	18/20
**Krebs 2014**	Yes	Yes	Yes	Yes	Yes	?	Yes	Yes	Yes	Yes	19/20
**Matthias 2014**	Yes	Yes	Yes	Yes	Yes	No	Yes	Yes	Yes	Yes	18/20
**McCrorie 2015**	Yes	Yes	?	Yes	Yes	No	Yes	Yes	Yes	Yes	17/20
**Mueller 2017**	Yes	Yes	Yes	Yes	?	No	Yes	Yes	?	Yes	16/20
**Paterson 2016**	Yes	Yes	Yes	Yes	Yes	?	?	Yes	Yes	Yes	18/20
**Penney 2017**	Yes	Yes	?	?	?	?	Yes	?	?	?	13/20
**Rieb 2016**	Yes	Yes	Yes	Yes	Yes	?	Yes	?	Yes	Yes	18/20
**Simmonds 2015**	Yes	Yes	?	Yes	Yes	?	Yes	Yes	?	?	16/20
**St Marie 2016**	Yes	Yes	Yes	Yes	Yes	?	Yes	Yes	Yes	Yes	19/20
**Vallerand 1 2009**	Yes	Yes	Yes	Yes	Yes	No	No	Yes	?	Yes	15/20
**Vallerand 2 2010**	Yes	Yes	Yes	?	Yes	No	No	Yes	?	?	13/20
**Wallace 2014**	Yes	Yes	Yes	Yes	Yes	No	Yes	?	?	Yes	16/20
**Warms 2005**	Yes	Yes	Yes	Yes	Yes	No	No	Yes	Yes	Yes	16/20
**Zgierska 2016**	Yes	Yes	Yes	Yes	Yes	No	Yes	Yes	Yes	Yes	18/20
**Zheng 2013**	Yes	Yes	Yes	Yes	Yes	No	?	Yes	Yes	Yes	17/20
**Al Achkar 2017**	Yes	Yes	Yes	Yes	Yes	No	Yes	Yes	Yes	Yes	18/20
**Matthias 2017**	Yes	Yes	Yes	Yes	Yes	No	Yes	Yes	Yes	Yes	18/20
**Matthias 2018**	Yes	Yes	Yes	Yes	Yes	?	Yes	Yes	Yes	Yes	19/20
**Smith 2018**	Yes	Yes	Yes	Yes	Yes	Yes	Yes	Yes	Yes	Yes	20/20

Legend 1 Critical Appraisal Skills Programme (CASP) questions scoring: Yes =2 ? (Can't Tell) = 1 No = 0

Q1. Was there a clear statement of the aims of the research?
Q2. Is a qualitative methodology appropriate?
Q3. Was the research design appropriate to the aims of the research?
Q4. Was the recruitment strategy appropriate to the aims of the research?
Q5. Was the data collected in a way that addressed the research issue?
Q6. Has the relationship between researcher and participants been adequately considered?
Q7. Have ethical issues been taken into consideration?
Q8. Was the data analysis sufficiently rigorous?
Q9. Is there a clear statement of findings?
Q10. How valuable is the research?

For peer review only

For peer review only - http://bmjopen.bmj.com/site/about/guidelines.xhtml

BMJ Open

A systematic review and qualitative evidence synthesis of the experiences of people taking opioid medication for chronic non-malignant pain: a meta-ethnography

Journal:	*BMJ Open*
Manuscript ID	bmjopen-2019-032988.R2
Article Type:	Original research
Date Submitted by the Author:	13-Dec-2019
Complete List of Authors:	Nichols, Vivien; University of Warwick, Warwick Clinical Trials Unit, Warwick Medical School<br>Toye, Francine; Oxford University Hospitals NHS Trust, Physiotherapy Research Unit, Nuffield Orthopaedic Centre<br>Eldabe, Sam; The James Cook University Hospital<br>Sandhu, Harbinder; University of Warwick, Warwick Clinical Trials Unit, Warwick Medical School<br>Underwood, Martin; University of Warwick, Warwick Clinical Trials Unit, Warwick Medical School<br>Seers, Kate; University of Warwick, Warwick Research in Nursing, Division of Health Sciences, Warwick Medical School
**Primary Subject Heading**:	Qualitative research
Secondary Subject Heading:	Patient-centred medicine
Keywords:	opioid, patients' views, QUALITATIVE RESEARCH, chronic non-malignant pain, meta-ethnography, qualitative evidence synthesis

SCHOLARONE™
Manuscripts

I, the Submitting Author has the right to grant and does grant on behalf of all authors of the Work (as defined in the below author licence), an exclusive licence and/or a non-exclusive licence for contributions from authors who are: i) UK Crown employees; ii) where BMJ has agreed a CC-BY licence shall apply, and/or iii) in accordance with the terms applicable for US Federal Government officers or employees acting as part of their official duties; on a worldwide, perpetual, irrevocable, royalty-free basis to BMJ Publishing Group Ltd ("BMJ") its licensees and where the relevant Journal is co-owned by BMJ to the co-owners of the Journal, to publish the Work in this journal and any other BMJ products and to exploit all rights, as set out in our licence.

The Submitting Author accepts and understands that any supply made under these terms is made by BMJ to the Submitting Author unless you are acting as an employee on behalf of your employer or a postgraduate student of an affiliated institution which is paying any applicable article publishing charge ("APC") for Open Access articles. Where the Submitting Author wishes to make the Work available on an Open Access basis (and intends to pay the relevant APC), the terms of reuse of such Open Access shall be governed by a Creative Commons licence – details of these licences and which Creative Commons licence will apply to this Work are set out in our licence referred to above.

Other than as permitted in any relevant BMJ Author's Self Archiving Policies, I confirm this Work has not been accepted for publication elsewhere, is not being considered for publication elsewhere and does not duplicate material already published. I confirm all authors consent to publication of this Work and authorise the granting of this licence.

For peer review only

A systematic review and qualitative evidence synthesis of the experiences of people taking opioid medication for chronic non-malignant pain: a meta-ethnography

Mrs Vivien Nichols (1) Dr Francine Toye (2) Professor Sam Eldabe (3) Dr Harbinder Sandhu (1) Professor Martin Underwood (1,) and Professor Kate Seers (4)

(1) Warwick Clinical Trials Unit
Warwick Medical School
University of Warwick, UK

(2) Physiotherapy Research Unit, Nuffield Orthopaedic Centre,
Oxford University Hospitals NHS Foundation Trust
Oxford, UK

(3) Academic Pain Department
The James Cook University Hospital
Middlesbrough, UK

(4) Warwick Research in Nursing
Warwick Medical School
University of Warwick, UK

Corresponding author:
Mrs Vivien Nichols
Warwick Clinical Trials Unit,
Warwick Medical School,
University of Warwick
Coventry CV4 7AL
Tel: 02476574652
Email: V.P.Nichols@warwick.ac.uk

For peer review only

Abstract

Objective To review qualitative studies of the experience of taking opioid medication for Chronic Non-Malignant Pain (CNMP) or coming off them.

Design A qualitative evidence synthesis using meta-ethnography. We used a seven step approach from the methods of meta-ethnography.

Data sources and eligibility criteria We searched selected databases: Medline, Embase, AMED, CINAHL, PsycInfo, Web of Science and Scopus (Science citation index and Social Science Citation Index) for qualitative studies which gave patients' views of taking opioid medication for CNMP or of coming off them (June 2017, updated September 2018).

Data extraction and synthesis Papers were quality appraised using a Critical Appraisal Skills Programme (CASP) tool and Grading of Recommendations Assessment, Development and Evaluation working group - Confidence in Evidence from Reviews of Qualitative research (GRADE-CERQual) guidelines were applied. We identified concepts and iteratively abstracted these concepts into a line of argument.

Results We screened 2129 unique citations, checked 153 full texts and 31 met our review criteria. We identified five themes: 1) Reluctant users with little choice; 2) Understanding opioids: the good and the bad; 3) A therapeutic alliance: not always on the same page; 4) Stigma: feeling scared and secretive but needing support; and 5) The challenge of tapering or withdrawal. A new overarching theme of 'constantly balancing' emerged from the data.

Conclusions People taking opioids were constantly balancing tensions, not always wanting to take opioids, weighing the pros and cons of opioids but feeling they had no choice because of the pain. They frequently felt stigmatised, were not always 'on the

For peer review only - http://bmjopen.bmj.com/site/about/guidelines.xhtml

same page' as their health care professional and changes in opioid use were often challenging.

Key words: Opioid, patients' views, qualitative research, chronic non-malignant pain, meta-ethnography, qualitative evidence synthesis.

Word count 4,971

For peer review only

Strengths and Limitations

- To our knowledge this is the first qualitative evidence synthesis of patients' experiences of taking opioid medications.
- Meta-ethnography provides a thorough, systematic way of synthesising qualitative findings across multiple studies.
- Meta-ethnography provides the reviewer's interpretation of second order concepts.
- Using a GRADE-CERQual approach can assist in rating confidence in the review findings.
- Qualitative research that illuminates patients' perspectives can help to shape future approaches to opioid management.

Introduction

Chronic non-malignant pain (CNMP) affects between an estimated 11% and 20% of the population in Europe and US and can impact heavily on people's quality of life [1, 2]. Opioid medications are strong painkillers which have a well-established role in the

For peer review only - http://bmjopen.bmj.com/site/about/guidelines.xhtml

treatment of acute and cancer pain; they have also been advocated for CNMP. Opioids can have distressing side effects as dosages increase such as; constipation, sedation, drowsiness, nausea, decreased concentration and memory, or mood changes [3]. Most people who use opioids develop tolerance to the painkilling effect of opioids, and some become dependent on them. Studies have shown that high opioid usage can also put people's lives at risk [4]. Despite this, the prescription of opioid medication for CNMP has risen sharply in the higher income countries. Few studies of opioids have shown effectiveness beyond 12 weeks follow up. Population surveys have shown long-term use to be associated with increased side effects and limited pain relief [3 5 6]. This synthesis of qualitative research was undertaken to underpin a process evaluation for the Improving the Wellbeing of people with Opioid Treated CHronic pain (I-WOTCH) study funded by the National Institute for Health Research. I-WOTCH is a randomised controlled trial evaluating a multi-component education and patient centred group intervention with a one-to-one tapering programme against a control of an advice booklet with a relaxation CD. More information can be found in the main study protocol [7] and process evaluation protocol [8].

This qualitative evidence synthesis uses the methods of meta-ethnography to find out what peoples' experiences are of both using opioids for CNMP and their attempts to stop taking them.

Methods

We use Noblit and Hare's 7 stages of meta-ethnographic analysis [9]. We used the new eMERGe reporting guidelines for meta-ethnography to structure our report [10] (*See appendix 1).* The protocol is published in the international prospective register of

For peer review only - http://bmjopen.bmj.com/site/about/guidelines.xhtml

systematic reviews (PROSPERO) registration number: CRD42017082418.

http://www.crd.york.ac.uk/PROSPERO

Step 1 Getting Started.

In order to address what has been labelled an opioid epidemic[11], we need to understand people's experiences of being on opioids and of coming off them. Our team was chosen because of its expertise in primary qualitative research and qualitative evidence synthesis specific to chronic pain and opioid prescription.

Step 2 Deciding what is relevant.

We undertook systematic electronic searches in June 2017 with a rerun in September 2018, appraising relevant papers for quality using the Critical Appraisal Skills Programme (CASP) tool for qualitative research [12]. One researcher (VN) with the assistance of an academic librarian (SJ) searched seven electronic databases; Medline, Embase, AMED, CINAHL, PsycInfo Web of Science and Scopus (Science citation index and Social Science Citation Index) and forward citation searches. We used search terms, free text and MeSH terms for all opioid drugs as well as their generic names. We combined these with the MeSH term 'pain' and a wide range of MeSH terms and words to describe all types of qualitative research and its analysis based upon a search used by Toye, Seers and Barker in 2017 [13]. The search was limited to those in English regarding humans with no cut-off date. Appendix 2 shows an example of our search terms.

Unique citations were screened independently by 2 researchers (VN ST *see acknowledgements*) against our inclusion and exclusion criteria (see Box 1). Any disagreements were arbitrated by a third researcher (KS). Papers for full text reading were identified and read by two researchers. Quality was assessed using a CASP tool. VN critically appraised the studies and KS independently appraised 10% for

For peer review only - http://bmjopen.bmj.com/site/about/guidelines.xhtml

consistency. The CASP scores are shown in table 1 and appendix 3. The GRADE-CERQual was used to appraise the reviewers' confidence in the research findings [14, 15].

Box 1 Inclusion/Exclusion criteria

**Included Studies**
Adults (18 years or older) taking or have taken opioid medication in the last five years
Published in English in peer review journals with no time constraint
Must relate to patient perspectives on using opioid medication for chronic non-malignant pain
Must use qualitative methodology (any analytical approach) or mixed quantitative and qualitative methodology with qualitative findings reported separately
Where studies include participants with differing medication we will include studies where the experience of those taking opioids is reported separately
**Excluded studies**
Paediatric studies (age less than 18 years)
Theoretical or methodological papers
Purely quantitative studies or mixed methods studies where the qualitative data are not presented separately
Studies concerning active cancer
Studies concerning headache
Studies concerning any acute, or acute postoperative, pain
Studies concerned only with heath care professional or carer perspectives, or studies of mixed carer/ patient/ professional populations where patient perspectives are not presented separately
Non- English language studies
Theses or conference abstracts which are not peer reviewed

Step 3 Reading the studies

VN read all the studies and KS and FT read half of these papers each (so all were read twice) and all extracted the second order concepts independently. A second order concept is a researcher's interpretation of data in a primary qualitative study [16]. VN, KS and FT met to discuss and reach agreement, and compiled a spreadsheet of all of the concepts extracted from the papers

Step 4 Determining how the studies are related?

VN sorted the concepts into categories by looking for any similarities and differences across all the studies. VN, KS and FT discussed the categorisation of data on multiple occasions. To enable comparison across studies, VN recorded descriptive data about each study (see table 1)

For peer review only - http://bmjopen.bmj.com/site/about/guidelines.xhtml

For peer review only

For peer review only - http://bmjopen.bmj.com/site/about/guidelines.xhtml

Table 1 Study characteristics with CASP and GRADE-CERQual relevance ratings

Studies	Country	Data collection / participants	Analytical Approach	Aims *Italics = verbatim quotes*	Morphine Equivalent Daily Dose mg/day (MED)	CASP score	Relevance
**1 Arnaert et al 2006**[17] Response Phases in Methadone Treatment for Chronic Non-malignant Pain	Canada	Semi-structured interview N=11 (4M/7F)	Content analysis	*"...to develop an understanding and to gain knowledge about the beliefs of patients with CNMP who were taking methadone, and to explore what challenges, if any, existed that affected their beliefs when first starting methadone treatment as an activity in their personal lives."*	None reported	17/20	P
**2 Bergman et al 2013** [18]Contrasting Tensions Between Patients and PCPs in Chronic Pain Management: A Qualitative Study	USA	In-depth interviews N=26 (24m/2F)	Inductive thematic analysis	*"... to develop a better understanding of the respective experiences, perceptions, and challenges both patients with chronic pain and PCPs face communicating with each other about pain management in the primary care setting."*	None reported	17/20	P
**3 Blake et al 2007**[19] Experiences of patients requiring strong opioid drugs for chronic non-cancer pain: a patient initiated study	UK	One focus group N=4 (2M/2F) and interviews N=10 (3M/5F)	Interpretative Phenomenological Analysis	*"...to determine the attitudes and experiences of patients receiving long-term strong opioid medication for chronic non-cancer pain in primary care."*	Individual opioid dosages	19/20	R
**4 Brooks et al 2015** [20]Exploring the lived experience of adults using prescription opioids to manage chronic noncancer pain	Canada	In-depth interviews N=9 (4M/5F)	Interpretative Phenomenological Analysis	*"...to explore the lived experience of adults using prescription opioids to manage CNCP, focusing on how opioid medication affected their daily lives."*	None reported	17/20	R

For peer review only - http://bmjopen.bmj.com/site/about/guidelines.xhtml

**5 Buchbinder et al 2014** [21]''Is there any way I can get something for my pain?'' Patient strategies for requesting analgesics	USA	Audio recorded clinical encounters N=74 (37M/37F)	Qualitative approach based on conversational analysis	*"We examined the direct and indirect means by which patients express a desire for analgesic medication."*	None reported	18/20	I
**6 Chang Y-P et al 2011** [22] Use of Prescription Opioid Medication among Community-Dwelling Older Adults with Noncancer Chronic Pain	USA	Face to face interview N=21 (13M/8F) ≥65yrs	Content analysis, mixed methods	*"… to: (1) describe older adults' patterns of adherence to their prescription opioid medication regimens and their reasons for these medication use patterns; and (2) examine the associations between adherence of prescription opioids, pain intensity, and pain interference on daily activity."*	None reported	16/20	P
**7 Chang,F et al 2017** [23]Perceptions of Community-Dwelling Patients and Their Physicians on OxyContin ® Discontinuation and the Impact on Chronic Pain Management	Canada	Pt interviews N=13 1M/ 12F	Not specified, no references given	*"… to explore the perceptions of patients with chronic pain and their physicians on OxyContin discontinuation and the impact that it had on chronic pain care and management."*	None reported	16/20	P
**8 Coyne et al 2015** [24] Assessment of a Stool Symptom Screener and Understanding the Opioid-Induced Constipation Symptom Experience	USA	Cognitive interview semi structured think-aloud approach N=66 (27M/39F)	Content analysis	*"…to evaluate the content validity of the Stool Symptom Screener by assessing patient understanding of its items, patient ability to differentiate among response options, and patient perceptions about the use of a 2-week recall period for evaluating stool symptoms, BMs, and laxative use.* *- to evaluate how patients describe their constipation experience and to understand*	None reported	17/20	I

For peer review only - http://bmjopen.bmj.com/site/about/guidelines.xhtml

				whether this differs between patients who frequently use laxatives and those who do not."			
**9 Esquibel et al 2014** [25] Doctors and patients in pain: Conflict and collaboration in opioid prescription in primary care	USA	In depth interviews with pts with CNCP receiving opioids and their physicians. N=21 pts (8M/13F)	Immersion/ crystallization process to generate a thematic codebook. Mixed methods with some quant. data analysis	*"…to better understand the effects of COT [chronic opioid therapy] on the doctor–patient relationship."*	None reported	20/20	R
**10 Frank et al 2016** [26] Patients' Perspectives on Tapering of Chronic Opioid Therapy: A Qualitative Study	USA	Semi-structured interviews N=24 (11M/13F)	Team based, mixed inductive and deductive approach guided by the Health Belief Model	*"… to explore patients' perspectives on opioid tapering."*	MED: Used algorithm<br><br>Median (IQR) 70 (30-165) Range 15-1845	20/20	R
**11 Green et al 2017** [27] Perpetuating stigma or reducing risk? Perspectives from naloxone consumers and pharmacists on pharmacy-based naloxone in 2 states	USA	8 focus groups (only 2 with patients with chronic pain) N=15 (8M/7F)	Thematic analysis. Content analysis with a collaborative codebook development process	*"…to explore the attitudes of various stakeholders toward pharmacy-based naloxone and opioid medication safety and to capture the range of initial experiences with pharmacy naloxone in 2 states with pharmacy naloxone policies."*	None reported	18/20	P

For peer review only - http://bmjopen.bmj.com/site/about/guidelines.xhtml

**12 Hooten et al 2011** [28] Smoking Cessation and Chronic Pain: Patient and Pain Medicine Physician Attitudes	USA	N = 18 Interviews N= 15 and focus group N=3 10M/8F	Participatory research, mixed methods - Thematic and content analysis	*"...to determine the attitudes and beliefs of both patients and pain medicine physicians regarding smoking cessation interventions applied during pain therapy."*	MED - used equianalgesic conversion software programme Mean ± SD 227 ± 356	18/20	I
**13 Krebs et al 2014** [29] Barriers to Guideline-Concordant Opioid Management in Primary Care—A Qualitative Study	USA	Semi structured interviews N= 26 patients (14 PC physicians) 13M/13F	Immersion crystallization approach	*"...to better understand primary care physicians' and patients' perspectives on recommended opioid management practices and to identify potential barriers and facilitators of guideline concordant opioid management in primary care."*	None reported	16/20	P
**14 Matthias et al 2014** [30] Communicating about opioids for chronic pain: A qualitative study of patient attributions and the influence of the patient–physician relationship	USA	Audio Recordings of primary care visits and Semi-structured interviews after clinic visit. N=30 26M/4F	Immersion/crystallization approach	*"...to advance understanding about communication about opioids by directly capturing clinical communication about opioids, as well as interviewing patients to gain insight into communication patterns and their broader relationships with their physicians, and how these relationships shape communication about opioids."*	None reported	18/20	R
**15 McCrorie et al 2015**[31] Understanding long-term opioid prescribing for non-cancer pain in primary care: a qualitative study	UK	Semi structured interviews with patients (focus groups with	Grounded approach for thematic analysis. Constant comparison	*"... to understand the processes which bring about and perpetuate long-term prescribing of opioids for chronic, non-cancer pain. We interviewed patients and GPs about their experiences, beliefs and expectations of analgesia prescribing, to improve understanding of how*	None reported	17/20	R

For peer review only - http://bmjopen.bmj.com/site/about/guidelines.xhtml

		GPs) N=23 6M/17F		*problematic long-term opioid prescribing becomes established".*			
**16 Mueller et al 2016** [32] Attitudes Toward Naloxone Prescribing in Clinical Settings: A Qualitative Study of Patients Prescribed High Dose Opioids for Chronic Non-Cancer Pain	USA	Semi-stuctured interview N=24 8M/16F	Ethnographic iterative methods. Inductive and deductive approaches	*"This study assessed the knowledge and attitudes toward naloxone prescribing among non-cancer patients prescribed opioids in primary care."*	Inclusion criteria ≥100mg MED	16/20	I
**17 Paterson et al 2016** [33] Resisting Prescribed Opioids: A Qualitative Study of Decision Making in Patients Taking Opioids for Chronic Noncancer Pain	Australia	Qualitative individual interviews N=20	Constant comparison and decision making explored in depth and with a thematic analysis utilizing a published "Model of medicine-taking"	*"…to identify the varying influences on patients' decisions about their use of prescribed long-term opioids. The research questions are: 1) does this conceptual Model of medicine-taking apply to people taking prescribed opioids, 2) is the concept of "resistance" to medication useful in this context, and 3) does this concept lead to new insights which may improve communication and shared decision making between"*	None reported	18/20	P
**18 Penney et al 2016** [34] Provider and patient perspectives on opioids	USA	11 Focus groups N=80 and	Thematic coding ref	*"…to identify the practical issues patients and providers face when accessing alternatives to*	None reported	13/20	P

For peer review only - http://bmjopen.bmj.com/site/about/guidelines.xhtml

and alternative treatments for managing chronic pain: a qualitative study		individual interviews N=10 27M/63F	Ryan, Bernard 2003	*opioids, and how multiple parties view these issues."*			
**19 Rieb et al 2016** [35] Withdrawal-associated injury site pain (WISP): a descriptive case series of an opioid cessation phenomenon	Canada	In person semi structured interviews N=21 14M/7F	Deductive and inductive approaches. Use of survey and emergent interview themes	*".... to document the existence and characteristics of this pain phenomenon that we have named withdrawal-associated injury site pain (WISP)."*	Recalled dose before WISP	18/20	P
**20 Simmonds et al 2015** [36] A Qualitative Study of Veterans on Long-Term Opioid Analgesics: Barriers and Facilitators to Multimodality Pain Management	USA	Semi structured focus groups x 3 N=25 17M/8F	Grounded theory-informed approach. Framework provided by the theory of planned behaviour	*"...to examine barriers and facilitators to multimodality chronic pain care among veterans on high-dose opioid analgesics for chronic non-cancer pain."*	Inclusion criteria at least 50 mg MED	16/20	P
**21 St Marie et al 2016** [37] Primary care experiences of people who live with chronic pain and receive opioids to manage pain: A qualitative methodology	USA	Semistructured interviews. N=12 6M/6F	Thematic and interpretive analyses	*"...to provide a deeper understanding of the experiences, issues, and challenges that people face who live with chronic pain and received opioids to help manage their pain in primary care. The following research questions were addressed: (a) What are the experiences of individuals who live with chronic pain and receive opioid pain medications to manage their pain in*	None reported	19/20	R

For peer review only - http://bmjopen.bmj.com/site/about/guidelines.xhtml

				primary care? (b) What have been their healthcare experiences as they strive to manage their pain?"			
**22 Vallerand et al 1 2009** [38] Chronic Opioid Therapy for Nonmalignant Pain: The Patient's Perspective. Part I— Life Before and After Opioid Therapy	USA	In depth serial interviews N=22 6M/16F	Phenomenologic study / Constant comparative method	*"…to examine the lived experience of adults receiving opioid therapy for relief of chronic non-malignant pain through the examination of data obtained through serial taper recorded interviews."*	Range 22.5 to 3,200	14/20	R
**23 Vallerand et al 2 2010** [39] Chronic Opioid Therapy for Non-malignant Pain: The Patient's Perspective. Part II— Barriers to Chronic Opioid Therapy	USA	In depth serial narrative interviews N=22 6M/16F	Phenomenologic study / Constant comparative method	*"…to elucidate the essence of the lived experience of patients receiving chronic opioid therapy for chronic non-malignant pain through examining their life narratives."*	None reported	13/20	R
**24 Wallace et al 2014** [40] Voices that may not otherwise be heard: a qualitative exploration into the perspectives of primary care patients living with chronic pain	USA	Photovoice photos, Interviews 1st N=31 2nd N=25 , and focus groups N=19	Grounded theory	*"…this study examined the utility of a combination of qualitative methods (Photovoice, one-on-one interviews, and focus groups) in examining the daily experiences of primary care patients living with chronic pain".*	None reported	16/20	R
**25 Warms et al 2005** [41] There are a few things you did not ask about my pain: writing on the margins of a survey	USA	Comments written in the margins of a questionnaire N=797	Content analysis	*"…to determine the characteristics of those who wrote comments* [written in the margin of survey questionnaire] *and to understand what was being communicated in their comments."*	None reported	16/20	P

For peer review only - http://bmjopen.bmj.com/site/about/guidelines.xhtml

				*that comments were written in the margin is a potential limitation.			
**26 Zgierska et al 2016** [42] Mindfulness Meditation-Based Intervention Is Feasible, Acceptable, and Safe for Chronic Low Back Pain Requiring Long-Term Daily Opioid Therapy	USA	Qual data on treatment satisfaction and experience N= 17	Qualitative analysis methods. Grounded theory	*"... to determine feasibility, acceptability, and safety of an MM-based intervention in patients with CLBP requiring daily opioid therapy."*	Inclusion criteria ≥30mg MED<br><br>Mean ± SD 166.9 ± 153.7	18/20	I
**27 Zheng et al 2013** [43]Chaos to Hope: A Narrative of Healing	Australi a	In depth qual interviews (N=20) 10M/10F	Illness narratives. Thematic analysis	*"...to investigate the progression of the illness and opioid journeys of people who are taking opioids for chronic non-cancer pain".*	None reported	17/20	P
**Rerun of search**							
**28 Al Achkar et al 2017** [44] Exploring perceptions and experiences of patients who have chronic pain as state prescription opioid policies change: a qualitative study in Indiana	USA	N=9 3M/6F	Inductive emergent thematic analysis	*"...to evaluate the impact of Indiana's opioid prescription legislation on the patient experiences around pain management."*	None reported	18/20	R

For peer review only - http://bmjopen.bmj.com/site/about/guidelines.xhtml

**29 Matthias et al 2017** [45] "I'm Not Gonna Pull the Rug out From Under You": Patient-Provider Communication About Opioid Tapering	USA	N=37 12M/25F	Inductive approach, constant comparison	*"…to understand communication processes related to opioid tapering."*	None reported	18/20	R
**30 Matthias et al 2018** [46] "I Was a Little Surprised": Qualitative Insights From Patients Enrolled in a 12-Month Trial Comparing Opioids With Nonopioid Medications for Chronic Musculoskeletal Pain	USA	N=34 28M/6F	Inductive approach, constant comparison	*"…to understand patients' experiences with the SPACE trial including their views and anticipated benefits of opioids, versus non -opioids, experiences with the intervention and to what extent expectations were met after completing the study."*	None reported	19/20	R
**31 Smith et al 2018** [47] Seeking Chronic Pain Relief: A Hermeneutic Exploration	USA	N=15 4M/11F	Concurrent embedded mixed methods design, Heideggerian hermeneutic phenomenological approach	*"This research presents an interpretation of the experience of seeking pain relief for a group of people taking opioid pain medications whose pain is not adequately controlled"*	None reported	20/20	P

Legend: GRADE-CERQual Relevance component: R= Relevant, P= Partial relevance, I= Indirect relevance, U=Uncertain relevance

IQR = Interquartile Range, SD = Standard Deviation

For peer review only - http://bmjopen.bmj.com/site/about/guidelines.xhtml

Step 5 Translating studies into each other

Patterns and associations between categories were explored and all researchers felt that a line of argument approach as defined by Noblit and Hare [9] would be the most useful method to interpret the data.

Step 6 Synthesising Translations

Agreement was reached by clearly defining the over-arching or 3rd order concepts arising from the data. A third order concept is the reviewers' interpretation of second order concepts.

Step 7 Expressing the synthesis

We developed a conceptual model to show how the themes related to each other in a line of argument. (see figure 1)

Insert figure 1 about here.

Patient and Public Involvement

We did not involve patients or the public in our work.

Results

Two reviewers VN and ST screened 2994 titles or abstracts (after the removal of duplicates from the 5064 citations retrieved) and identified 153 full texts of interest. Two reviewers VN and KS read these and 122 were excluded. Reasons are given in the PRISMA flowchart, see figure 2. The reviewers agreed to include 31 studies. The included studies were from US (23), Canada (4) UK (2), and Australia (2) and used a range of qualitative methods.

We report the 4 facets of GRADE-CERQual for all papers. 1) Methodological 2) Confidence 3) Relevance 4) Adequacy of data - See table 2 and 3 below.

For peer review only - http://bmjopen.bmj.com/site/about/guidelines.xhtml

Table 2 Confidence in review findings: GRADE-CERQual assessment

Review findings	Studies contributing (see table 1 column 1 for study number)	Methodological limitations (study number)	Relevance (See table 1 end column)	Coherence	Adequacy of data
Reluctant users with little choice	1,3,4,5,6,7,17,18,21,22,26,27,30 (13 studies)	11 no concerns<br>2 minor concerns (18,22)	5 Relevant<br>6 Partial<br>2 Indirect	No concerns	No concerns
Understanding opioids: the good the bad	1,3,7,9,10,11,15, 16,17, 23,25, 27,29. (13 studies)	12 no concerns<br>1 minor concerns (23)	6 Relevant<br>6 Partial<br>1 Indirect	No concerns	No concerns
A therapeutic alliance: not always on the same page	1,2,3,4,5,7,9,10, 11,13,14,15,16, 17,18,19,20,21, 22,23,24,25,26, 28,29,31 (26 studies)	23 no concerns<br>3 minor concerns (18,22,23)	12 Relevant<br>11 Partial<br>3 Indirect	No concerns	No concerns
Stigma: feeling scared, and secretive but needing support	1,2,3,4,7,9,10,14,16,17,18,20,21, 22, 23, 24, 27, 28, 31 (19 studies)	16 no concerns<br>3 minor concerns (18,22,23)	10 Relevant<br>8 Partial<br>1 Indirect	No concerns	No concerns
The challenge of tapering/ withdrawal from opioids	7,10,18,19,30,31 (6 studies)	5 no concerns<br>1 minor concerns (18)	2 Relevant<br>4 Partial	Minor concerns	Minor concerns

Table 3 GRADE-CERQual component scoring

Methodological limitations<br>Coherence<br>Adequacy of data	No or very minor concerns<br>Minor concerns<br>Moderate concerns<br>Serious concerns
Relevance	Relevant<br>Partial<br>Indirect<br>Uncertain

Synthesis of Findings

We abstracted five themes from the 2nd order concepts. Table 4 below shows how each study contributed to each theme. We have illustrated each concept with exemplary quotations.

Table 4 Themes apparent in each study

Author date	RU	U	TA	S	TW

For peer review only - http://bmjopen.bmj.com/site/about/guidelines.xhtml

1 Arnaert and Ciccotosto 2006	X	X	X	X	
2 Bergman, Matthias et al 2013			X	X	
3 Blake, Ruel et al 2007	X	X	X	X	
4 Brooks, Unruh et al 2015	X		X	X	
5 Buchbinder, Wilbur et al 2014	X		X		
6 Chang Y-P, Wray et al 2011	X				
7 Chang,F and Ibrahim,S 2017	X	X	X	X	X
8 Coyne, Currie et al 2015					
9 Esquibel and Borkan 2014		X	X	X	
10 Frank, Levy et al 2016		X	X	X	X
11 Green, Case et al 2017		X	X		
12 Hooten, Vickers et al 2011					
13 Krebs, Bergman et al 2014			X		
14 Matthias,Krebs et al 2013			X	X	
15 McCrorie, Closs et al 2015		X	X		
16 Mueller, Koester et al 2016		X	X	X	
17 Paterson, Ledgerwood 2016	X	X	X	X	
18 Penney, Ritenbaugh et al 2016	X		X	X	X
19 Rieb, Norman et al 2016			X		X
20 Simmonds, Finley et al 2015			X	X	
21 St Marie et al 2015	X		X	X	
22 Vallerand et al 1 2009	X		X	X	
23 Vallerand et al 2 2010		X	X	X	
24 Wallace et al 2014			X	X	
25 Warms et al 2005 o		X	X		
26 Zgierska et al 2016	X		X		
27 Zheng et al 2013	X	X		X	
**Studies from search rerun**	**RU**	**U**	**TA**	**S**	**TW**
28 Al Achkar et al 2017			X	X	X
29 Matthias et al 2017		X	X		X
30 Matthias et al 2018	X				
31 Smith et al 2018			X	X	

Legend:
RU = Reluctant users with little choice
U = Understanding about opioids: the good and the bad
TA = A therapeutic alliance: not always on the same page
S = Stigma: feeling scared, and secretive but needing support
TW = The challenges of tapering or withdrawal
X = theme present in paper

For peer review only

1) Reluctant users with little choice

This describes a resistance or hesitancy to take opioids mainly due to concerns about side effects or addiction, although they felt there were no other options available.

For peer review only - http://bmjopen.bmj.com/site/about/guidelines.xhtml

"I don't want to become addicted, if I'm going to become addicted then as far as I'm concerned I'm a druggie, so I might as well not be here anyway, so I don't want to become addicted…" Blake et al Pg103

"I just didn't want to go on them because I mean once you get on them that's it, you're sort of stuck on them. I didn't want to take morphine at first because there was a girl that I went through one of the courses with and she always seemed really dopey and drugged up so it took them a long while to talk me to into taking the morphine because I didn't want to be like that. Zheng et al pg 1832

Some spoke of underusing or were keen to reduce their medications when possible. There was a dislike of being on long term medication and some thought that it would not relieve their pain.

"I don't want to do that [take more morphine]. *I want to stay on as little as I possibly can because there might come a time when I need more and I don't want to be on high doses. I've always tried to keep it at a minimum amount of tablets each day…"* Blake et al Pg 105

Even though some were reluctant there were other instances of dramatic improvement in people's lives. This then weighted their choice to stay on the opioids.

*"I mean it is just like a miracle as far as I am concerned. It is like knowing it [*the pain*] is there but you have the instruments to prevent it from getting out and [be]coming a roaring demon."* Vallerand et al 1pg 170

"But opiates, that's my way of life. There would be no life if I didn't have this. And I thank God for them because without them I'd be…well I wouldn't be. I just couldn't go on. I would have committed suicide a long time ago. And I say that truthfully cause you could not live like that, with that constant, constant

For peer review only - http://bmjopen.bmj.com/site/about/guidelines.xhtml

pain. But, with the opiates it's made it possible to be able to have a part of a life, you know." Brooks et al pg 20

2) Understanding opioids: the good and the bad

This describes patients' knowledge or understanding about opioids which had generally been acquired ad hoc and slowly over time, from pharmacists, patient package inserts in their medication, leaflets, the internet, television programmes and from doctors, especially doctors at the pain clinic.

"When you see it in the media, when you see it on the television, you think if you're taking regular morphine you must be in a pretty bad way, you know." Blake et al Pg103

"I always ask before I go on a medication, what are the side effects, I was told I may experience constipation; nothing else was explained to me." Paterson et al pg 721

There was often poor knowledge about using opioids for chronic pain, and about addiction, overdose risk and side effects.

"There's not too much education about it [overdose]… When I first started taking it [the opioid medication], no one told me about OD[overdose]or anything about that. Because I was taking it not [as] prescribed…I was just like when I felt pain I would just take like five or six of them or whatever. Then at the end, I'd run out." Mueller et al pg 279

Patients often had to defend their usage and this added to their stress especially when they felt their healthcare professionals lacked an understanding of the place for opioids in the treatment of CNMP or were cautious about using them.

For peer review only - http://bmjopen.bmj.com/site/about/guidelines.xhtml

"The concern is that if they increase my opioid dosage, I could stop breathing. It's ridiculous." Frank et al Pg1841

''There are still a lot of doctors out there that are against it. They think it is bad. Bad medicine. Bad practice.'' Vallerand et al 2 pg 129

In contrast, some people felt well informed which either produced more concern or gave the patients confidence in their opioid regime.

"… and from what I've read up, because I like to, sort of, keep on top of things, that it's an opium based drug, so you will build up some tolerance and you will build up [becomes tearful] And you will potentially become sort of addicted to it, if you like." McCrorie et al pg 3

"Under Dr A [pain clinic] I've learnt more. And my concern has been, well it was initially the possibility of addiction, but she has assured me that I'm not showing no signs of addiction at all. I may have some withdrawal problems" Paterson et al Pg 721

3) A therapeutic alliance: not always on the same page

This describes a therapeutic alliance or the relationship between patients and their health care providers which was considered important.

Overall there was a feeling that HCPs and patients were often 'not on the same page' about opioid usage.

"My family doctor…does not want me to be dependent on heavy pain meds, so I am intensely miserable 99% of the time." Warms et al pg 252

Some patients felt they were not listened to and were frustrated by a lack of empathy from physicians regarding their pain experience.

"I frequently have difficulty with the residents (doctors in training) explaining why these drugs, this many drugs…Finally Dr. [family physician] wrote a note

For peer review only - http://bmjopen.bmj.com/site/about/guidelines.xhtml

in my file – stop harassing [participant's name]. This is what she gets and why she gets it. And they did stop but it was inconvenient. For instance, they would not prescribe me three months at a time. I would be dispensed one month at a time. And for someone who had been taking the same drugs for 10 years I found that condescending." Brooks et al pg 18

A reluctance to prescribe from GPs and pharmacists and the use of opioid contracts or a restriction of medication were often considered punitive.

"It kind of made me feel like I was doing something wrong, which I wasn't, but I signed a contract. You know, what would I be without my meds?" Krebs et al pg 1152

"And I told my doctor that, that I wanted so I could sleep through the night. And now he, well, I'll give you 10, but it's got to last. Like he treats me like a drug addict." Penney et al pg 6

The healthcare system often worked against a therapeutic alliance with lack of continuity or care or frequent visits which fed into mistrust. Patients complained that provider turnover affected their ability to receive individualised care; conversations about pain and treatment options often had to be started over again from scratch.

"*I don't have the same doctor long enough to know*". Bergman et al pg 1693

However having blood or urine tests for levels of opioids and regular checks were seen by some as being cared for.

"*I would say, 'I have this agreement and you don't have to sign it if you don't want to, but I would like to go over it with you. These are suggestions because this medication is addictive, it is dangerous, and I just want to make sure you're aware.' I think if you really want to make it where people are not*

For peer review only - http://bmjopen.bmj.com/site/about/guidelines.xhtml

hostile, say they have to have a urine test every 6 months, everybody, and that 'it's a policy because we care about all of you.'" Krebs et al pg 1152

Some talked of the need for good relationships built on trust, shared decision making and knowledgeable specialists who communicate well.

"I wouldn't say I researched it to that depth, you know, I read a little bit about, and asked a lot of questions at my doctor, and then we decided." Paterson pg 722

4) Stigma: feeling scared and secretive but needing support

This describes feelings of stigma and fear which people expressed directly in relation to their opioid usage. This includes peoples' negative attitudes from family, medical professionals and work colleagues which lead to them feeling stigmatised and judged for taking opioids.

"So I'm constantly trying to clean up because I think people are going to judge me. 'Oh, because she's on all this medication, ooh, she can't look after her children.'" Paterson et al pg 724

"As soon as you mention to someone that you are on pain medication it's, 'Oh my god, you've got to get off it.' It is viewed as weak. Somehow I am weak for being on this medication." Vallerand et al 2 pg 128

To protect themselves some chose to keep their opioids a secret.

"But you know, after 2 years of pain, you are physically exhausted, mentally exhausted and depressed. So, I take my medication and I hide it at the bottom of my drawer. It's my secret life. It's always a secret, and I've got to hide it and not tell anyone." Vallerand et al 1 pg 169

For peer review only - http://bmjopen.bmj.com/site/about/guidelines.xhtml

Some people made a conscious decision about who they could tell and who they couldn't due to negative reactions. Relationships suffered when patients felt unsupported.

> *"My son told me I was a drug addict. He did. He really did. He was to the point, he didn't know what he could do for me. It really was that bad."* Vallerand et al 2 pg 128

> *"I had originally told my sister and she was very concerned. Then she said, 'As long as you don't stay on them.' She thought it was OK if I did it for a while but as long as I didn't stay on them. So I just sort of never told her. And she never asked."* Vallerand et al 2 pg 128

Although some seemed confident in using opioids, mostly people spoke about fears such as; addiction and uncontrolled pain. Feeling supported validated their choices and experiences and lessened some of their fears and concerns.

> *"And at the end, my partner says—we sat down there and he goes 'Stay on them.' …I've always spoke to my partner, and if he's been unsure— we've both been unsure, we've both gone into the doctor together to ask questions."* Zheng et al pg 1834

> *"my wife wanted me to take this medication. She was like: let's go for it."* Arnaert et al pg 26

5) The challenge of tapering/withdrawal from opioids

Four papers [23, 26 44, 45] explore patients' experiences of tapering or withdrawing as their main content. Two further papers [34, 35] addressed it as a more peripheral issue (see CERQual ratings in table 2). This describes the challenges and profound effects of tapering or withdrawing from opioids.

Tapering and withdrawing from opioids could be challenging and provoke anxiety.

For peer review only - http://bmjopen.bmj.com/site/about/guidelines.xhtml

"I have a tremendous fear in a doctor saying I want you to taper off the methadone and get totally off the methadone with no alternative whatsoever. I think that would be an irrational decision by a doctor, and I probably wouldn't take that advice." Frank et al pg1842

This anxiety could be alleviated by support from a trusted health care provider or other person.

"The best thing about it was that nobody acted like I was a bad person because I was on these medications and was having to be going through this really slow process of coming down off of them." Frank et al pg1843

Successful tapering was described as a collaborative agreement between HCP and patient.

''She put me down to 2 and a half [pills per day]. Then she said, okay, we'll go down to half a pill. I told her I didn't think that just 2 a day would do it, and she said okay, we'll try 2 and a half, are you agreeable with that? I said that's fine. I mean, we can discuss stuff. It doesn't have to be a disagreement because we can talk about it. It's not an argument. We're 2 adults having a conversation, figuring out what to do.'' Matthias et al 2017 pg 1369

However, not all people experienced joint decision making when tapering

''I just don't feel that he's understanding. he don't seem to care what I'm saying, because he's lowering it down anyway, even though I've told him…that I didn't agree with it being lowered.'' Matthias et al 2017 pg 1369

For those in the USA, prescribing policies, advising clinicians to monitor and decrease opioid use, and the legislation to enforce these policies made those taking opioids feel as if they were 'a public health problem'. This could have a negative

For peer review only - http://bmjopen.bmj.com/site/about/guidelines.xhtml

effect on the doctor patient relationship and leave the patient feeling disempowered. This was compounded when opioids had been withdrawn by legislation.[23, 44]

> *"I have to struggle, suffer, to make the next the next time that I can get my medicine. And I don't think that's fair to me because if I can take my medicine a little more regularly , I would be able to do more……I don't think that the law, people, politicians, or anybody should be able to tell anybody that's in pain what type of medicine they can take."* Al Achkar et al pg 7

> *"That kinda got me mad, cause I thought well you know. . .they're taking it off the market because of people abusing it. . .It's not fair to us, you know. . . . I think the government was wrong to. . . pull them off the market, you know, because of people abusing them, no like they weren't looking at the people that need them. . .But I think it's really unfair that people that really do need them can't get them."* Chang and Ibrahim 2017 pg 3

Overarching theme: Constantly balancing

After considering the fives themes, an overarching theme emerged - 'Constant balancing'. The theme *Reluctant users with little choice* describes the need to balance the pros and cons of starting opioids and the need to balance having pain with their hesitancy to use opioids.

> *"I don't really like being on a lot of tablets, I've never been a tablet person, um. . . but I mean I can't have the pain either so it's one evil outdoing the other evil.* Paterson et al pg 723

Studies describe balancing the dose for pain management with their side effects to allow them to function. Participants constantly weighed up the effects on their life; dealing with an internal conflict of unresolved pain versus necessary medication,

For peer review only - http://bmjopen.bmj.com/site/about/guidelines.xhtml

being opioid free versus having uncontrolled pain and balancing other stressors against opioid dose changes.

> *"If you're going to be able to walk, and you take one pain pill so you can walk and live life, you're going to do it, even though you may not like it."* Penney et al pg 6

The theme *Stigma feeling scared and secretive but needing support*, describes the need to balance their hopes for relief with fear of side effects, and also to balance whether or not to disclose their opioid use with the risk of being labelled a 'drug seeker' versus having unrelieved pain.

> "*I do it for my own protection by not telling them because I see how they react by reading something in the paper…and it's just their ignorance. And I don't have time. Well they know what's going on but they don't get it to this day. So you have to pick your battles…"* Brooks et al pg 19

The theme *Understanding opioids; the good and the bad*, showed people had different levels of understanding but weighed up their decisions and trade-offs against their pain relief.

> *"It's, it's got a good and bad side, morphine. ……When I take it, it works really, really well but it makes you feel rather sick, umm, rather spaced out and thinking wise, umm, it outcomes more on the other, do I want to be sick or do I want to cry with pain? So I'd rather be sick but it is a very, very good painkiller. "* Blake et al pg 105

The therapeutic alliance theme showed that often it was evident that they were 'not on the same page' with them balancing the advice from their doctors with what they wanted.

For peer review only - http://bmjopen.bmj.com/site/about/guidelines.xhtml

"[My provider] said you could die any time, and my husband and I said, well, we realize that, but because of the pain, you know, we were willing to take that risk that I would die from the narcotic medication." Frank et al pg 1841

It also meant that there were multiple barriers to the process of decreasing opioids due to this constant balancing act which is described in the theme *the challenge of tapering/withdrawal from opioids.*

"I will tell her, if I do come off this medication, there are going to be consequences. I can't walk as often, I can't stand as long, I just can't do it…." Vallerand et al 1 pg 169

Discussion

Our five themes were; 1**)** Reluctant users with little choice; 2) Understanding opioids: the good the bad; 3) A therapeutic alliance: not always on the same page; 4**)** Stigma: feeling scared and secretive but needing support; and 5) The challenge of tapering or withdrawal. An overarching theme of 'constantly balancing' emerged from the data. These themes all had positive and negative aspects although the negative were more prevalent by far.

We present a line of argument of how complex it is for the patient to balance decisions at every stage of their journey. First their reluctance to start taking opioids but feeling they had no option. Patients are given opioids for CNMP often as a last resort when all other treatment has failed and their lives are so profoundly affected that they talk of a desperation, that they would literally 'try anything'. Patients spoke about not being given any detailed information about opioids and that they had learned more about them over time from different sources. This varied understanding about opioids and their side effects can affect the decisions that people make. Patients reported the need to keep the dosage of opioids as low as possible and often that they

For peer review only - http://bmjopen.bmj.com/site/about/guidelines.xhtml

were not at risk of addiction or overdose if they were taking them as prescribed. Even those who felt they may be addicted sometimes viewed this as an acceptable trade-off for pain relief. Our findings indicate that patient desperation combined with inadequate information from healthcare professionals could trigger the prescription of opioids. It may be that delivering accurate information about the potential side effects and limited efficacy of opioids for chronic pain management would reduce the use of opioids.

Our findings demonstrate that the stigma surrounding how patients feel about being on opioids can be compounded by the judgements of others. Although patients often describe themselves in terms of 'reluctant users', if they experienced the benefits of opioids through decreased pain and thus increased function they are often too scared to reduce opioids and return to a life of potentially unmanaged pain.

Our findings suggest that clinicians and patients with chronic pain are not always 'on the same page'. The theme *a therapeutic alliance* captures the positives, but also the tensions and mismatches of perceptions held by healthcare providers who are attempting to limit dose escalation, and patients who may view constant dose escalation as an acceptable trade-off for reducing relentless pain. The therapeutic alliance is a robust theme supported by 26 of the 31 studies included. This is not surprising as patients rely on their health care professionals to prescribe opioids. This finding resonates with qualitative evidence syntheses (QES) exploring the experience of patients [13] and healthcare professionals [48] It seems clear that joint decision -making is important for appropriate healthcare; however, our findings suggests that there are instances of mistrust on both sides. A QES exploring clinicians experience of prescribing opioids for chronic pain demonstrate that the process of prescribing

For peer review only - http://bmjopen.bmj.com/site/about/guidelines.xhtml

opioids is not straightforward for clinicians who face a complex decision - 'Should I shouldn't I' prescribe opioids for chronic non-malignant pain [48]. They also demonstrate that clinicians must walk a fine line to balance the pros and cons of opioids whilst also maintaining patient trust. This suggests that both patients and HCPs find dealing with prescribing/taking opioids for CNMP is complex and involves balancing and trade-offs.

Current guidance from Royal College of Anaesthetists in the UK and The Centers for Disease Control and Prevention in the US advocate a preference for non-opioid therapies in the treatment of CNMP[49]. If a clinician feels that opioids are indicated, then they recommend a low dose for a short duration which should be assessed for effectiveness and regularly evaluated for benefits and harms. All but four studies in this review are between 2005 and 2017, prior to these guidelines. Opioid contracts in some areas of the USA and Canada can make patients feel stigmatised and judged, this effect can be moderated by a good therapeutic relationship, and reframing these as agreements rather than contracts[50]. Some physicians may view contracts/agreements as necessary to guard against uncontrolled dose escalation, repeated demands for replacement of lost or misplaced medication, subversion and illicit opioid intake. This finding resonates with Toye et al (2017) who describe the moral boundary work and social guardianship that clinicians associate with opioid prescription. Our findings suggest that this role does may not contribute to an effective therapeutic partnership.

Limitations of this study

A majority of the studies are from the United States and the findings need to be taken in the context of its health and social care systems. Most of the articles in this qualitative synthesis were published or the research was conducted, before the impact

For peer review only - http://bmjopen.bmj.com/site/about/guidelines.xhtml

of the opioid epidemic became clear to regulators and the medical profession. Some papers discuss using opioids as a last resort, although the opioid epidemic, especially in the US indicates that the threshold for prescribing opioids was low until recent initiatives to discourage prescribing long-term opioids for chronic pain[51]. Not all studies gave morphine equivalent data so we cannot determine what proportion were taking high, medium or low doses. We acknowledge that our interpretation of the data might have been influenced by the current, much more critical perception of opioid use for chronic non-malignant pain. Further evidence is needed to find out if these themes are universal for developed countries or whether there are important differences.

Our conceptual framework highlights patients need to constantly balance and to consider the pros and cons of taking opioids. This can have a profound effect on peoples' relationships with their family, friends and health care providers and their perceived standing in the community which is reflected in their careful balancing of disclosure. The therapeutic alliance and having a clear understanding of all the positive and negative aspects of opioids were important factors that underpinned their ability to maintain this fragile balance. This balance might also affect a person's desire or ability to taper or withdraw from opioids.

The GRADE-CERQual ratings (table 2) revealed we had confidence in the findings with only a few minor concerns and no moderate or serious concerns.

Conclusions and recommendations for future research

The first meta-ethnography on this topic revealed a constant balancing and a life in flux in an effort to maintain participation in life and relationships. These are important features of opioid use for CNMP. To maintain this delicate balance they often need support from family or clinicians, however this balance can be upset by the feeling of

For peer review only - http://bmjopen.bmj.com/site/about/guidelines.xhtml

being judged by this same potential support system or peers and society at large through the media. The therapeutic alliance with healthcare professionals, the extent of people's understanding as well as the stigma attached to opioid use need to be navigated by people who are often reluctant to be on opioids in the first place.

Authors Contribution

VN and KS contributed to the review concept and design as part of the I-WOTCH process evaluation team. HS, SE, MU and KS were involved in the design of the IWOTCH study. VN, KS and FT screened search results or extracted data, conducted the analysis and synthesis. All authors contributed to data interpretation, revised the final manuscript critically for important intellectual content and appraised the final manuscript. VN prepared the final manuscript and will be the corresponding author.

Funding

This project was funded by the National Institute for Health Research, Health Technology Assessment (project number 14/224/04). The views and opinions expressed therein are those of the authors and do not necessarily reflect those of the HTA, NIHR, NHS or the Department of Health. Ethics approval was provided by Yorkshire and the Humber South Yorkshire Research Ethics committee on 13-9-16.

Declaration of competing interests

KS has undertaken other meta-ethnographies and was on the NIHR HS&DR Board until January 2018.

SE is investigator on a number of NIHR and industry sponsored studies. He received travel expenses for speaking at conferences from the professional organisations. SE consults for Medtronic, Abbott, Boston Scientific and Mainstay Medical, none in

For peer review only - http://bmjopen.bmj.com/site/about/guidelines.xhtml

relation to opioids. SE is chair of the BPS Science and Research Committee. SE is deputy Chair of the NIHR CRN Anaesthesia Pain and Perioperative Medicine National Specialty Group. SE's department has received fellowship funding from Medtronic as well as nurse funding from Abbott.

HS is director of Health Psychology Services Ltd, providing psychological services for a range of health related conditions.

MU was Chair of the NICE accreditation advisory committee until March 2017 for which he received a fee. He is chief investigator or co-investigator on multiple previous and current research grants from the UK National Institute for Health Research, Arthritis Research UK and is a co-investigator on grants funded by the Australian NHMRC. He is an NIHR Senior Investigator. He has received travel expenses for speaking at conferences from the professional organisations hosting the conferences. He is a director and shareholder of Clinvivo Ltd that provides electronic data collection for health services research. He is part of an academic partnership with Serco Ltd related to return to work initiatives. He is a co-investigator on a study receiving support in kind from Stryker Ltd. He has accepted honoraria for teaching from CARTA He is an editor of the NIHR journal series, and a member of the NIHR Journal Editors Group, for which he receives a fee.

Data Availability Statement: Data is available on reasonable request.

Acknowledgements

We would like to thank Samantha Johnson an academic librarian who helped with the electronic searches and Dr Stephanie Tierney who helped to screen the citations.

References

For peer review only - http://bmjopen.bmj.com/site/about/guidelines.xhtml

1. Breivik H, Collett B, Ventafridda V, et al. Survey of chronic pain in Europe: prevalence, impact on daily life, and treatment. *Eur J Pain*2006;10:287-333.
2. Nahin RL. Estimates of pain prevalence and severity in adults: United States, 2012. *J Pain*2015;16:769-80.
3. Els C, Jackson TD, Kunyk D, et al. Adverse events associated with medium- and long-term use of opioids for chronic non-cancer pain: an overview of Cochrane Reviews. *Cochrane Database of Systematic Reviews*2017.
4. Gomes T, Tadrous M, Mamdani MM, et al. The burden of opioid-related mortality in the united states. *JAMA Network Open*2018;1:e180217.
5. Furlan AD, Sandoval JA, Mailis-Gagnon A, et al. Opioids for chronic noncancer pain: a meta-analysis of effectiveness and side effects. *CMAJ*2006;174:1589-94.
6. Busse JW, Wang L, Kamaleldin M, et al. Opioids for Chronic Noncancer Pain: A Systematic Review and Meta-analysis. *JAMA*2018;320:2448-60.
7. Sandhu HK, Abraham C, Alleyne S, et al. Testing a support programme for opioid reduction for people with chronic non-malignant pain: the I-WOTCH randomised controlled trial protocol. *BMJ Open*2019;9:e028937.
8. Nichols VP, Abraham C, Eldabe S, et al. Process evaluation protocol for the I-WOTCH study: an opioid tapering support programme for people with chronic non-malignant pain. *BMJ Open*2019;9:e028998.
9. Noblit GW and Hare RD. Meta-ethnography: Synthesizing qualitative studies. sage, 1988.
10. France EF, Cunningham M, Ring N, et al. Improving reporting of meta-ethnography: The eMERGe reporting guidance. *J Adv Nurs*2019;75:1126-39.
11. Manchikanti L, Helm S, 2nd, Fellows B, et al. Opioid epidemic in the United States. *Pain Physician*2012;15:ES9-38.
12. Critical Appraisal Skills Programme (CASP). Oxford: Public Health Resource Unit, 2006.
13. Toye F, Seers K and Barker KL. Meta-ethnography to understand healthcare professionals' experience of treating adults with chronic non-malignant pain. *BMJ Open*2017;7:e018411.
14. Lewin S, Booth A, Glenton C, et al. Applying GRADE-CERQual to qualitative evidence synthesis findings: introduction to the series. *Implement Sci*2018;13:2.
15. Lewin S, Bohren M, Rashidian A, et al. Applying GRADE-CERQual to qualitative evidence synthesis findings-paper 2: how to make an overall CERQual assessment of confidence and create a Summary of Qualitative Findings table. *Implement Sci*2018;13:10.
16. Schutz AJCP. Collected Papers. Vol. 1. The Hague: Martinus Nijhoff.. 1964. 1962;2.
17. Arnaert A and Ciccotosto G. Response phases in methadone treatment for chronic nonmalignant pain. *Pain Management Nursing*2006;7:23-30.
18. Bergman AA, Matthias MS, Coffing JM, et al. Contrasting tensions between patients and PCPs in chronic pain management: a qualitative study. *Pain Medicine*2013;14:1689-97.
19. Blake S, Ruel B, Seamark C, et al. Experiences of patients requiring strong opioid drugs for chronic non-cancer pain: a patient-initiated study. *British Journal of General Practice*2007;57:101-8.

For peer review only - http://bmjopen.bmj.com/site/about/guidelines.xhtml

20. Brooks EA, Unruh A and Lynch ME. Exploring the lived experience of adults using prescription opioids to manage chronic noncancer pain. *Pain Research & Management*2015;20:15-22.
21. Buchbinder M, Wilbur R, McLean S, et al. "Is there any way I can get something for my pain?" Patient strategies for requesting analgesics. *Patient Education and Counseling*2015;98:137-43.
22. Chang Y-P, Wray LO, Sessanna L, et al. Use of prescription opioid medication among community-dwelling older adults with noncancer chronic pain. *Journal of Addictions Nursing (Taylor & Francis Ltd)*2011;22:19-24.
23. Chang F and Ibrahim S. Perceptions of community-dwelling patients and their physicians on OxyContin ® discontinuation and the impact on chronic pain management. *Pain Research & Management*2017;2017:11.
24. Coyne KS, Currie BM, Holmes WC, et al. Assessment of a Stool Symptom Screener and Understanding the Opioid-Induced Constipation Symptom Experience. *Patient*2015;8:317-27.
25. Esquibel AY and Borkan J. Doctors and patients in pain: Conflict and collaboration in opioid prescription in primary care. *Pain*2014;155:2575-82.
26. Frank JW, Levy C, Matlock DD, et al. Patients' Perspectives on Tapering of Chronic Opioid Therapy: A Qualitative Study. *Pain Medicine*2016;17:1838-47.
27. Green TC, Case P, Fiske H, et al. Perpetuating stigma or reducing risk ? Perspectives from naloxone consumers and pharmacists on pharmacy-based naloxone in 2 states. *Journal of the American Pharmacists Association*2017;57:S19-+.
28. Hooten WM, Vickers KS, Shi Y, et al. Smoking cessation and chronic pain: patient and pain medicine physician attitudes. *Pain Practice*2011;11:552-63.
29. Krebs EE, Bergman AA, Coffing JM, et al. Barriers to guideline-concordant opioid management in primary care-a qualitative study. *Journal of Pain*2014;15:1148-55.
30. Matthias MS, Krebs EE, Bergman AA, et al. Communicating about opioids for chronic pain: A qualitative study of patient attributions and the influence of the patient–physician relationship. *European Journal of Pain*2014;18:835-43.
31. McCrorie C, Closs SJ, House A, et al. Understanding long-term opioid prescribing for non-cancer pain in primary care: a qualitative study. *BMC Family Practice*2015;16:121.
32. Mueller SR, Koester S, Glanz JM, et al. Attitudes Toward Naloxone Prescribing in Clinical Settings: A Qualitative Study of Patients Prescribed High Dose Opioids for Chronic Non-Cancer Pain. *Journal of General Internal Medicine*2017;32:277-83.
33. Paterson C, Ledgerwood K, Arnold C, et al. Resisting Prescribed Opioids: A Qualitative Study of Decision Making in Patients Taking Opioids for Chronic Noncancer Pain. *Pain Medicine*2016;17:717-27.
34. Penney LS, Ritenbaugh C, DeBar LL, et al. Provider and patient perspectives on opioids and alternative treatments for managing chronic pain: a qualitative study. *BMC Family Practice*2017;17.
35. Rieb LM, Norman WV, Martin RE, et al. Withdrawal-associated injury site pain (WISP): A descriptive case series of an opioid cessation phenomenon. *Pain*2016;157:2865-74.
36. Simmonds MJ, Finley EP, Vale S, et al. A qualitative study of veterans on long-term opioid analgesics: barriers and facilitators to multimodality pain management. *Pain Medicine*2015;16:726-32.

For peer review only

37. St Marie B. Primary care experiences of people who live with chronic pain and receive opioids to manage pain: A qualitative methodology. *Journal of the American Association of Nurse Practitioners*2016;28:429-35.
38. Vallerand A and Nowak L. Chronic Opioid Therapy for Nonmalignant Pain: The Patient's Perspective. Part I-Life Before and After Opioid Therapy. *Pain Management Nursing*2009;10:165-72.
39. Vallerand A and Nowak L. Chronic opioid therapy for nonmalignant pain: the patient's perspective. Part II--Barriers to chronic opioid therapy. *Pain Management Nursing*2010;11:126-31.
40. Wallace LS, Wexler RK, McDougle L, et al. Voices that may not otherwise be heard: A qualitative exploration into the perspectives of primary care patients living with chronic pain. *Journal of Pain Research*2014;7:291-99.
41. Warms CA, Marshall HM, Hoffman AJ, et al. There are a few things you did not ask about my pain: writing on the margins of a survey questionnaire. *Rehabilitation Nursing*2005;30:248-56.
42. Zgierska AE, Burzinski CA, Cox J, et al. Mindfulness Meditation-Based Intervention Is Feasible, Acceptable, and Safe for Chronic Low Back Pain Requiring Long-Term Daily Opioid Therapy. *Journal of Alternative & Complementary Medicine*2016;22:610-20.
43. Zheng Z, Paterson C, Ledgerwood K, et al. Chaos to hope: a narrative of healing. *Pain Medicine*2013;14:1826-38.
44. Al Achkar M, Revere D, Dennis B, et al. Exploring perceptions and experiences of patients who have chronic pain as state prescription opioid policies change: a qualitative study in Indiana. *BMJ open*2017;7:e015083.
45. Matthias MS, Johnson NL, Shields CG, et al. "I'm Not Gonna Pull the Rug out From Under You": Patient-Provider Communication About Opioid Tapering. *Journal of Pain*2017;18:1365-73.
46. Matthias MS, Donaldson MT, Jensen AC, et al. "I Was a Little Surprised": Qualitative Insights From Patients Enrolled in a 12-Month Trial Comparing Opioids With Nonopioid Medications for Chronic Musculoskeletal Pain. *Journal of Pain*2018;19:1082-90.
47. Smith CL, Severtsen B, Vandermause R, et al. Seeking Chronic Pain Relief: A Hermeneutic Exploration. *Pain Management Nursing*2018.
48. Toye F, Seers K, Tierney S, et al. A qualitative evidence synthesis to explore healthcare professionals' experience of prescribing opioids to adults with chronic non-malignant pain. *BMC Fam Pract*2017;18:94.
49. CDC. CDC Guideline for Prescribing Opioids for Chronic Pain: (2019, accessed 12-4-19).
50. Tobin DG, Keough Forte K and Johnson McGee S. Breaking the pain contract: A better controlled-substance agreement for patients on chronic opioid therapy. *Cleve Clin J Med*2016;83:827-35.
51. Schieber LZ, Guy GP, Jr., Seth P, et al. Trends and Patterns of Geographic Variation in Opioid Prescribing Practices by State, United States, 2006-2017. *JAMA Netw Open*2019;2:e190665.

Figure 1 Concept model
Figure 2 PRISMA flow diagram

For peer review only - http://bmjopen.bmj.com/site/about/guidelines.xhtml

Figure 1 Concept model of the experiences of people taking opioid medication for chronic non-malignant pain

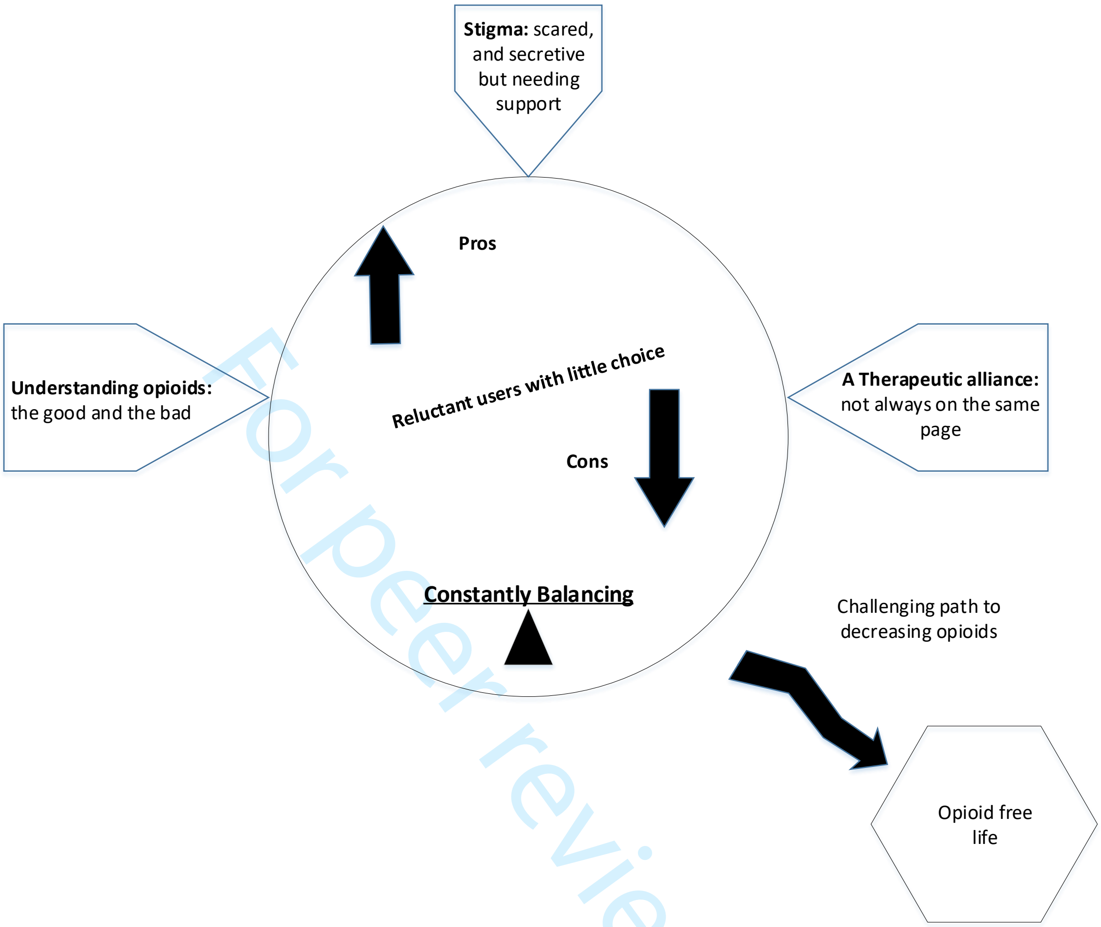

For peer review only

Figure 2: PRISMA Flow Diagram

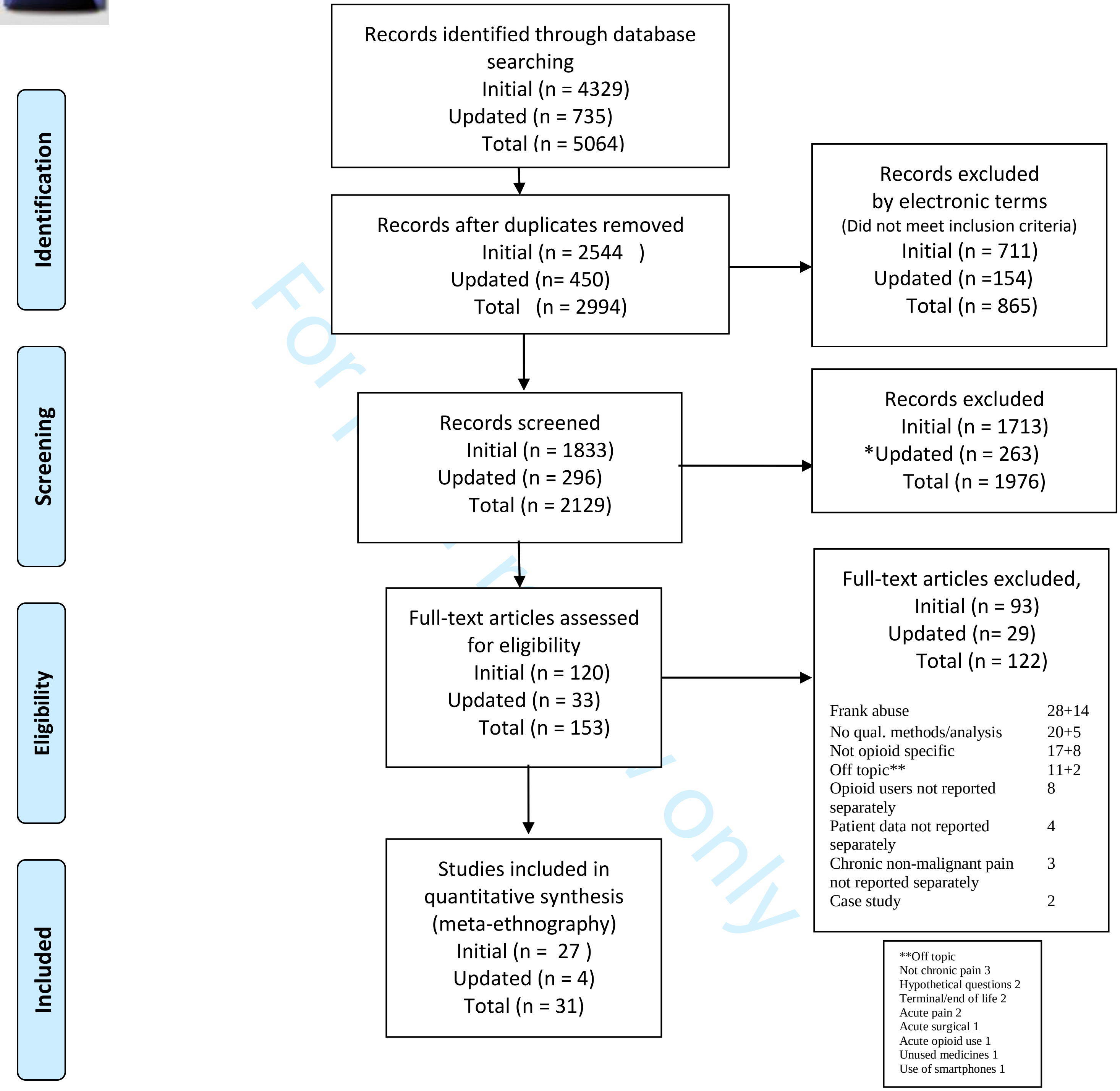

Initial search June 2017 and updated search Sept 2018 reported.

The search dates for the rerun could be specified in some databases, but not all. Therefore some papers were found in both searches. These twice found duplicate papers were removed at this point*

From: Moher D, Liberati A, Tetzlaff J, Altman DG, The PRISMA Group (2009). *P*referred *R*eporting *I*tems for *S*ystematic Reviews and *M*eta-*A*nalyses: The PRISMA Statement. PLoS Med 6(7): e1000097. doi:10.1371/journal.pmed1000097

For peer review only - http://bmjopen.bmj.com/site/about/guidelines.xhtml

Appendix 1 The eMERGe meta-ethnography reporting guidance

Numbered criteria headings with explanatory reporting criteria	Page
**Phase 1—Selecting meta-ethnography and getting started**	
Introduction<br>1 Rationale and context for the meta-ethnography<br>Describe the gap in research or knowledge to be filled by the meta-ethnography, and the wider context of the meta-ethnography	4
2 Aim(s) of the meta-ethnography<br>Describe the meta-ethnography aim(s)	4
3 Focus of the meta-ethnography<br>Describe the meta-ethnography review question(s) (or objectives)	4
4 Rationale for using meta-ethnography<br>Explain why meta-ethnography was considered the most appropriate qualitative synthesis methodology	4
**Phase 2—Deciding what is relevant**	
Methods<br>5 Search strategy<br>Describe the rationale for the literature search strategy	4/5
6 Search processes<br>Describe how the literature searching was carried out and by whom	4/5
7 Selecting primary studies<br>Describe the process of study screening and selection, and who was involved	5
Findings<br>8 Outcome of study selection<br>Describe the results of study searches and screening	16
**Phase 3—Reading included studies**	
Methods<br>9 Reading and data extraction approach<br>Describe the reading and data extraction method and processes	6
Findings<br>10 Presenting characteristics of included studies<br>Describe characteristics of the included studies	Table1
**Phase 4—Determining how studies are related**	
Methods<br>11 Process for determining how studies are related<br>Describe the methods and processes for determining how the included studies are related: - Which aspects of studies were compared AND- How the studies were compared	6
Findings<br>12 Outcome of relating studies<br>Describe how studies relate to each other	17
**Phase 5—Translating studies into one another**	16
Methods<br>13 Process of translating studies<br>Describe the methods of translation: - Describe steps taken to preserve the context and meaning of the relationships between concepts within and across studies- Describe how the reciprocal and refutational translations were conducted- Describe how potential alternative interpretations or explanations were considered in the translations	16
Findings<br>14 Outcome of translation<br>Describe the interpretive findings of the translation.	17 to 28
**Phase 6—Synthesizing translations**	
Methods<br>15 Synthesis process<br>Describe the methods used to develop overarching concepts ("synthesised translations")Describe how potential alternative interpretations or explanations were considered in the synthesis	16
Findings<br>16 Outcome of synthesis process<br>Describe the new theory, conceptual framework, model, configuration, or interpretation of data developed from the synthesis	Figure 1
**Phase 7—Expressing the synthesis**	

For peer review only

Discussion<br>17 Summary of findings<br>Summarize the main interpretive findings of the translation and synthesis and compare them to existing literature	28 to 30
18 Strengths, limitations, and reflexivity<br>Reflect on and describe the strengths and limitations of the synthesis:<br>- Methodological aspects—for example,describe how the synthesis Findings were influenced by the nature of the included studies and how the meta-ethnography was conducted.<br>- Reflexivity—for example, the impact of the research team on the synthesis findings	30
19 Recommendations and conclusions<br>Describe the implications of the Synthesis	31

Reference: France et al. BMC Medical Research Methodology (2019) 19:25
https://doi.org/10.1186/s12874-018-0600-0

For peer review only

Appendix 2 – example of search terms

Scopus<br>((TITLE-ABS-KEY(buprenorphine or fentanyl or heroin or hydromorphone or methadone or morphine or opium or oxycodone or pentazocine or tramadol or opiate* or opioid*) OR TITLE-ABS-KEY(suboxone or bunavail or zubsolv or dipipanone or diconal or wellconal or diamorphine or duragesic or fentora or actiq or abstral or recivit or effentora or instanyl or pecfent or oxycontin or roxicodone or oramorph or papaveretum or omnopon or fortral)OR TITLE-ABS-KEY(sosegon or "talwin nx" or pethidine or meperidine or demerol or tapentadol or nucynta or palexia or tapal or ultram or zytram))) and ((TITLE-ABS-KEY(qualitative w/5 (theor* or study or studies or research or analysis)) OR TITLE-ABS-KEY(ethno* or emic or etic or phenomenolog* or hermeneutic* or heidegger* or husserl* or colaizzi* or giorgi* or glaser or strauss or (van and kaam*) or (van and manen) or ricoeur or spiegelberg* or merleau) OR TITLE-ABS-KEY(constant w/3 compar*) OR TITLE-ABS-KEY(focus w/3 group*) OR TITLE-ABS-KEY(grounded w/3 (theor* or study or studies or research or analysis)) OR TITLE-ABS-KEY(narrative w/3 analysis) OR TITLE-ABS-KEY(discourse w/3 analysis) OR TITLE-ABS-KEY((lived or life) w/3 experience*) OR TITLE-ABS-KEY((theoretical or purposive) w/3 sampl*) OR TITLE-ABS-KEY("field note*" or "field record*" or fieldnote*) OR TITLE-ABS-KEY(participant* w/3 observ*) OR TITLE-ABS-KEY("action research") OR TITLE-ABS-KEY("digital adj record*" or audiorecord* or taperecord* or videorecord* or videotap*) OR TITLE-ABS-KEY(cooperative and inquir*) OR TITLE-ABS-KEY(co and operative and inquir*) OR TITLE-ABS-KEY(co-operative and inquir*) OR TITLE-ABS-KEY(("semi-structured" or semistructured or unstructured or structured) w/3 interview*) OR TITLE-ABS-KEY((Informal or in-depth or indepth or "in depth") w/3 interview*) OR TITLE-ABS-KEY(("face-to-face" or "face to face") w/3 interview*) OR TITLE-ABS-KEY("ipa" or "interpretive phenomenological analysis") OR TITLE-ABS-KEY(social and construct*) OR TITLE-ABS-KEY("appreciative inquiry") OR TITLE-ABS-KEY(poststructural* or "post structural*" or post-structural*) OR TITLE-ABS-KEY(postmodern* or "post modern*" or post-modern*) OR TITLE-ABS-KEY(feminis*) OR TITLE-ABS-KEY(humanistic or existential or experiential))) and (TITLE-ABS-KEY(pain))

For peer review only

For peer review only

Appendix 3 Critical Appraisal Skills Programme (CASP) Quality appraisal tool scores

Study	Q1	Q2	Q3	Q4	Q5	Q6	Q7	Q8	Q9	Q10	Score
**Arnaert 2006**	Yes	Yes	Yes	Yes	?	No	Yes	Yes	Yes	Yes	17/20
**Bergman 2013**	Yes	Yes	Yes	Yes	Yes	No	Yes	?	Yes	Yes	17/20
**Blake 2007**	Yes	Yes	Yes	Yes	Yes	?	Yes	Yes	Yes	Yes	19/20
**Brooks 2015**	Yes	Yes	Yes	Yes	Yes	No	Yes	?	Yes	Yes	17/20
**Buchbinder 2015**	Yes	Yes	Yes	Yes	Yes	No	Yes	Yes	Yes	Yes	18/20
**Chang F 2017**	Yes	Yes	Yes	Yes	Yes	No	?	?	Yes	Yes	16/20
**Chang Y-P 2011**	Yes	Yes	Yes	Yes	Yes	No	Yes	?	?	Yes	16/20
**Coyne 2015**	Yes	Yes	Yes	Yes	Yes	No	Yes	?	Yes	Yes	17/20
**Esquibel 2014**	Yes	Yes	Yes	Yes	Yes	Yes	Yes	Yes	Yes	Yes	20/20
**Frank 2016**	Yes	Yes	Yes	Yes	Yes	Yes	Yes	Yes	Yes	Yes	20/20
**Green 2017**	Yes	Yes	Yes	Yes	Yes	No	Yes	Yes	Yes	Yes	18/20
**Hooten 2011**	Yes	Yes	Yes	Yes	Yes	No	Yes	Yes	Yes	Yes	18/20
**Krebs 2014**	Yes	Yes	Yes	Yes	Yes	?	Yes	Yes	Yes	Yes	19/20
**Matthias 2014**	Yes	Yes	Yes	Yes	Yes	No	Yes	Yes	Yes	Yes	18/20
**McCrorie 2015**	Yes	Yes	?	Yes	Yes	No	Yes	Yes	Yes	Yes	17/20
**Mueller 2017**	Yes	Yes	Yes	Yes	?	No	Yes	Yes	?	Yes	16/20
**Paterson 2016**	Yes	Yes	Yes	Yes	Yes	?	?	Yes	Yes	Yes	18/20
**Penney 2017**	Yes	Yes	?	?	?	?	Yes	?	?	?	13/20
**Rieb 2016**	Yes	Yes	Yes	Yes	Yes	?	Yes	?	Yes	Yes	18/20
**Simmonds 2015**	Yes	Yes	?	Yes	Yes	?	Yes	Yes	?	?	16/20
**St Marie 2016**	Yes	Yes	Yes	Yes	Yes	?	Yes	Yes	Yes	Yes	19/20
**Vallerand 1 2009**	Yes	Yes	Yes	Yes	Yes	No	No	Yes	?	Yes	15/20
**Vallerand 2 2010**	Yes	Yes	Yes	?	Yes	No	No	Yes	?	?	13/20
**Wallace 2014**	Yes	Yes	Yes	Yes	Yes	No	Yes	?	?	Yes	16/20
**Warms 2005**	Yes	Yes	Yes	Yes	Yes	No	No	Yes	Yes	Yes	16/20
**Zgierska 2016**	Yes	Yes	Yes	Yes	Yes	No	Yes	Yes	Yes	Yes	18/20
**Zheng 2013**	Yes	Yes	Yes	Yes	Yes	No	?	Yes	Yes	Yes	17/20
**Al Achkar 2017**	Yes	Yes	Yes	Yes	Yes	No	Yes	Yes	Yes	Yes	18/20
**Matthias 2017**	Yes	Yes	Yes	Yes	Yes	No	Yes	Yes	Yes	Yes	18/20
**Matthias 2018**	Yes	Yes	Yes	Yes	Yes	?	Yes	Yes	Yes	Yes	19/20
**Smith 2018**	Yes	Yes	Yes	Yes	Yes	Yes	Yes	Yes	Yes	Yes	20/20

Legend 1 Critical Appraisal Skills Programme (CASP) questions scoring: Yes =2 ? (Can't Tell) = 1 No = 0

Q1. Was there a clear statement of the aims of the research?
Q2. Is a qualitative methodology appropriate?
Q3. Was the research design appropriate to the aims of the research?
Q4. Was the recruitment strategy appropriate to the aims of the research?
Q5. Was the data collected in a way that addressed the research issue?
Q6. Has the relationship between researcher and participants been adequately considered?
Q7. Have ethical issues been taken into consideration?
Q8. Was the data analysis sufficiently rigorous?
Q9. Is there a clear statement of findings?
Q10. How valuable is the research?

For peer review only
